# Postoperative pain treatment after total knee arthroplasty: A systematic review

**DOI:** 10.1371/journal.pone.0173107

**Published:** 2017-03-08

**Authors:** Anders Peder Højer Karlsen, Mik Wetterslev, Signe Elisa Hansen, Morten Sejer Hansen, Ole Mathiesen, Jørgen B. Dahl

**Affiliations:** 1 Department of Anaesthesia, Bispebjerg and Frederiksberg Hospital, Copenhagen, Denmark; 2 Department of Anaesthesia, Zealand University Hospital, Koege, Denmark; 3 Department of Anaesthesia, Rigshospitalet, Copenhagen University Hospital, Copenhagen, Denmark; 4 Department of Anaesthesia, Slagelse Hospital, Slagelse, Denmark; 5 Department of Anaesthesia, 4231, Centre of head and Orthopaedics, Rigshospitalet, Copenhagen, Denmark; Massachusetts General Hospital, UNITED STATES

## Abstract

**Introduction:**

The aim of this systematic review was to document efficacy, safety and quality of evidence of analgesic interventions after total knee arthroplasty (TKA).

**Methods:**

This PRISMA-compliant and PROSPERO-registered review includes all-language randomized controlled trials of medication-based analgesic interventions after TKA. Bias was evaluated according to Cochrane methodology. Outcomes were opioid consumption (primary), pain scores at rest and during mobilization, adverse events, and length of stay. Interventions investigated in three or more trials were meta-analysed. Outcomes were evaluated using forest plots, Grading of Recommendations Assessment, Development and Evaluation (GRADE), L’Abbe Plots and trial sequential analysis.

**Results:**

The included 113 trials, investigating 37 different analgesic interventions, were characterized by unclear/high risk of bias, low assay sensitivity and considerable differences in pain assessment tools, basic analgesic regimens, and reporting of adverse events. In meta-analyses single and continuous femoral nerve block (FNB), intrathecal morphine, local infiltration analgesia, intraarticular injection of local anaesthetics, non-steroidal anti-inflammatory drugs, and gabapentinoids demonstrated significant analgesic effects. The 24-hour morphine-sparing effects ranged from 4.2 mg (CI: 1.3, 7.2; intraarticular local anaesthetics), to 16.6 mg (CI: 11.2, 22; single FNB). Pain relieving effects at rest at 6 hours ranged from 4 mm (CI: -10, 2; gabapentinoids), to 19 mm (CI: 8, 31; single FNB), and at 24 hours from 3 mm (CI: -2, 8; gabapentinoids), to 16 mm (CI: 8, 23; continuous FNB). GRADE-rated quality of evidence was generally low.

**Conclusion:**

A low quality of evidence, small sample sizes and heterogeneity of trial designs prohibit designation of an optimal procedure-specific analgesic regimen after TKA.

## Introduction

The primary goals of postoperative analgesic treatment are to reduce pain, opioid requirements and consequently opioid-related adverse events, in order to optimize rehabilitation. Enhancing these outcomes has potential beneficial influence on patient morbidity and satisfaction, the degree of required postoperative care, as well as economic perspectives. Total knee arthroplasty (TKA) is a frequently performed orthopedic procedure followed by moderate to severe pain. Therefore, an efficient postoperative analgesic treatment based on sound evidence from the published literature is important for this procedure [1]. Recent research on postoperative pain after total hip arthroplasty suggest, however, that it may be difficult to allow a designation of a “best proven intervention” from the available scientific evidence [2], and it is reasonable to believe that this applies for TKA as well.

The hypothesis of this review was, that no globally recognized, best proven, gold standard analgesic treatment or intervention exists for TKA. The aim of this systematic review of all randomized, controlled clinical trials (RCTs) considering postoperative pain treatment after TKA is therefore to document the evidence for postoperative analgesic interventions after TKA.

## Materials and methods

The review meets requirements of the Preferred Reporting Items for Systematic reviews and Meta-Analyses (PRISMA) statement [3]. Registration in the PROSPERO International prospective register of systematic reviews was completed on April 23, 2014, prior to initiation of the study (registration number: CRD42014014940). Updated searches were carried out on June 17, 2016, and September 19, 2016, and registered in the protocol as amendments.

Our methods are similar to those reported in a recent review of postoperative pain treatment after total hip arthroplasty (THA) published by our research group [2]. As the two reviews are associated the methods and results sections are reported in a similar way to secure uniformity.

### Literature search

Trials were sought in Pubmed, Embase and The Cochrane Library according to S1 Appendix. The last search date was September 9, 2016. The PROSPECT database [4] and reference lists were screened for eligible trials as well.

### Inclusion criteria

Inclusion criteria were randomized controlled trials of unilateral total knee arthroplasty that compared postoperative analgesic outcomes of a perioperative analgesic intervention against placebo in a control group. Basic analgesic regimens and rescue analgesics had to be administered under equal conditions in the intervention and control groups. Trials where different rescue analgesics were administered, e.g. morphine and acetaminophen p.n., were included for qualitative analyses, but not meta-analyses. We only included trials with interventions initiated in the immediate perioperative period that reported either opioid-sparing effect, pain at rest or pain during mobilization. Trials concerning knee fractures, trials including patients less than 18 years, and data published in summary clinical trials, editorials, letters, and comments were excluded.

### Outcomes

The primary outcome was 0–24 hours postoperative cumulated opioid consumption.

Secondary outcomes were pain both at rest and on mobilization at 6 and 24 hours postoperatively, opioid related and intervention associated adverse events, and length of hospital stay (LOS).

### Data extraction

We extracted the following data: Trial sample size; basic analgesic regimen (i.e. analgesics administered to both intervention- and control group as a fixed regimen); rescue analgesics and 24 hour cumulated dose; pain score at rest and during movement at 6 ± 2 hours and 24 ± 4 hours postoperatively; opioid-related adverse events (postoperative nausea or vomiting (PONV), sedation, dizziness, pruritus, urinary retention, constipation and respiratory depression); intervention-associated adverse events as reported; LOS; and documented and predefined discharge criteria.

Assay sensitivity (a trials ability to detect an absolute difference between groups if there is one) was deemed low if a control group demonstrated a pain score on a visual analogue scale (VAS 0–100 mm) below 30 mm and/or a 0–24 hour cumulated i.v. morphine consumption below 15 mg.

Data extraction and bias evaluation was carried out by two authors independently. Disagreements were solved during meetings with all authors.

### Missing data

For trials with unclear bias domains or missing information regarding primary outcomes, the corresponding author was contacted by email and if unresponsive, another inquiry was sent two weeks later. We used open questions as "Please describe all measures taken to secure random sequence allocation" to avoid false confirmation on suggested measures.

### Bias assessment

We used the Cochrane bias assessment tool [5] to evaluate the following domains: Random sequence allocation, allocation concealment, blinding of participants and personnel, blinding of outcome assessors, incomplete outcome data, selective outcome reporting, and other potential threats to validity (including conflict of interest). Domains were rated as low, high, or unclear risk of bias. If all domains were low the summarized risk of bias was rated low; if one or more domains were high the summarized risk was rated high; and if one or more domains were unclear with no high risk domains, the summarized risk was rated unclear.

In addition, we evaluated trial sample size as a contributor to bias. A cumulated trial sample size of < 50 patients was rated as high risk of bias, 50–199 as moderate risk of bias, 200–499 as low risk of bias, and > 499 as very low risk of bias based on Dechartres et al. [6].

### Data analysis

#### Handling of data

Meta-analyses were carried out in Review Manager 5^®^ [7] whenever three or more trials regarding a specific intervention reported a 0–24 hour opioid consumption. Opioids were converted to i.v. morphine equivalents according to S2 Appendix. Pain scores, side effects and LOS were analyzed when reported in three ore more trials. Visual analogue scale (VAS 0–10) and Numerical Rating Scale 0–10 (NRS 0–10), were converted to VAS 0–100. Median and interquartile range (IQR)/range was converted to mean and standard deviation according to The Cochrane Handbook 7.7.3.5 [8], or Hozo et al [9], as appropriate. For results presented only as mean, a standard deviation was calculated from the p-value according to The Cochrane Handbook 7.7.3.3 [8], and we used the conservative approach p = 0.05 if the p-value was expressed as p < 0.05. Some trials had more than one intervention group. In these cases we either merged intervention groups or split the control group, according to The Cochrane Handbook 7.7.a [8].

Forest plots were calculated with a 95% confidence interval (CI) mean difference for continuous data and risk ratio (RR) with a 95% CI for dichotomous data. Random effects model was used whenever I^2 was above 30%. For I^2 between 0 and 30% fixed and random effects models were compared and the most conservative approach (the model with the widest 95% CI) was used to take into account the heterogeneity of included trials. P-values of less than 0.05 were considered statistically significant.

#### Heterogeneity

L’Abbé plots were conducted for each meta-analysed intervention to describe the degree of heterogeneity for morphine consumption and pain scores [10].

#### Strength of evidence

In meta-analyses, low information size (number of patients included) and repeated significance testing increase the risk of type I and II errors (false positive and false negative results, respectively). This risk can be reduced by performing trial sequential analysis (TSA) [11]. A forest plot describes whether the tested intervention reaches significance through the classic p<0.05, whereas TSA accounts for interim analyses and the heterogeneity of the trials as well. In TSA, the normal stationary threshold for significance with a Z-score at 1.96 for p = 0.05 is penalized if the included trials demonstrates a high degree of heterogeneity. An intervention with a high degree of heterogeneity requires a higher information size to reach the threshold for significance compared to a forest plot analysis. This is calculated as the a priori estimated information size (APIS).

TSA was performed for morphine consumption and pain scores, for all interventions that were included in meta-analyses.

We used Trial Sequential Analysis Viewer 0.9 Beta (The Copenhagen Trial Unit (CTU)) and followed the CTU guidelines (an alpha-value of 0.05 and a beta-value of 0.9) [12]. The sensitivity to detect a mean difference was set to 10 mg i.v. morphine equivalents/24 hours and 15 mm on a VAS 0–100 mm scale [13, 14].

#### Summary of findings

Quality of evidence was assessed with The Grading of Recommendations Assessment, Development and Evaluation (GRADE). Five factors were evaluated for each outcome: Study limitations; publication bias; indirectness of evidence; inconsistent results; and imprecision (evaluation based on results in TSA) [15].

Outcome effects and quality of evidence were summarized according to GRADE using GRADEpro 3.6.

## Results

### Retrieved trials

Search on Pubmed, EMBASE and The Cochrane Library identified 5126, 5806 and 2646 citations, respectively. The first author removed 4952 duplicates. Two authors assessed the remaining 8626 citations individually, compared results and consequently 287 trials were downloaded in full-text, of which 22 were written in a non-English language. We managed to acquire 285 trials of which 172 met one or more exclusion criteria (S3 Appendix).

**Fig 1:**
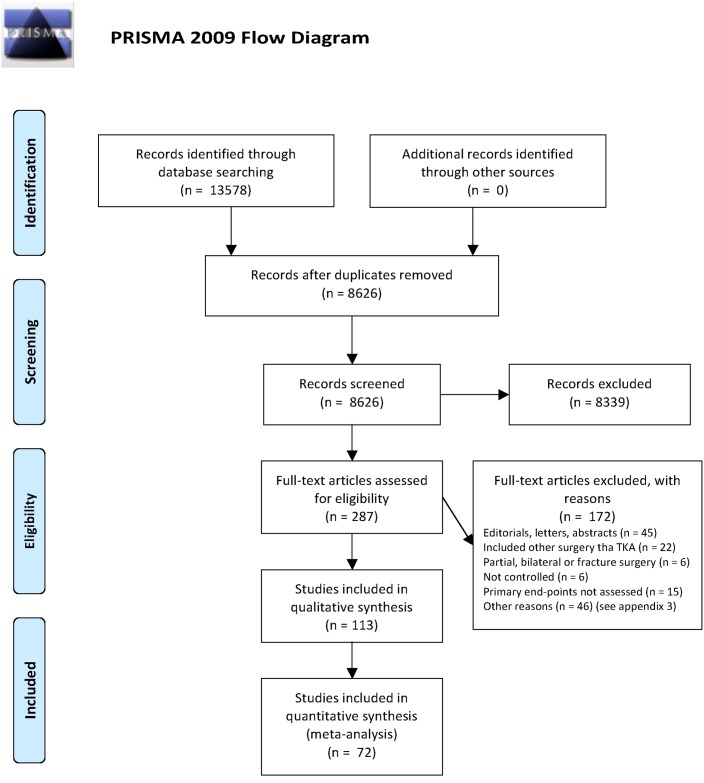
Flow chart of trial selection. *From*: Moher D, Liberati A, Tetzlaff J, Altman DG, The PRISMA Group (2009). *P*referred *R*eporting *I*tems for *S*ystematic Reviews and *M*eta-*A*nalyses: The PRISMA Statement. PLoS Med 6(7): e1000097. doi:10.1371/journal.pmed1000097. For more information, visit www.prisma-statement.org.

Thus, 113 randomized placebo-controlled trials concerning postoperative analgesic interventions after TKA were included for review (Fig 1 PRISMA flowchart). The total number of patients was 8407.

The included trials comprised 37 different treatment interventions. Interventions that qualified for meta-analyses, were single injection femoral nerve block (FNB), continuous FNB, intrathecal morphine, local infiltration analgesia (LIA), intraarticular injection with local anaesthetics, non-steroidal anti-inflammatory drugs (NSAIDs)/COX-2-inhibitors, and gabapentinoids.

Of all trials 36, 10, 3, and 1 had two, three, four and five separate intervention groups, respectively.

The follow-up period in the included trials was: 1 day in 20 trials, 2 days in 36 trials, 3 days in 16 trials, 4–7 days in 8 trials, ≥2 weeks in 22 trials, and unclear in 11 trials.

Detailed study information from the included trials is summarized in Table 1.

**Table 1:** Study information.

Author	Trial sample size intervention and control groups. Risk of trial sample size bias		Treatment intervention Group 1	Treatment intervention Group 2	Treatment intervention Control group	Basic analgesic regimen all groups	Type of supplemental analgesic	Assessment of pain scores				Length of stay assessment
								Rest 6 h 24 h		Movement 6h 24 h		
Allen, H. W. (1998) [16]	12/12/12	High (<50 patients)	FNB bupivacaine 0.25% 30 mL with epinephrine. Sham sciatic block	FNB and sciatic nerve block 30 mL 0.25% bupivacaine / epinephrine each	Sham sciatic and femoral nerve blocks	Ketorolac 15/30 mg i.v. x 4 (depending on age/weight).	PCA i.v. morphine	Yes	Yes	No	Yes	No
Chan, E. Y. (2013) [17]	69/66	Moderate (50–199 patients)	FNB bupivacaine 0.25% 20 mL with epinephrine	Continuous FNB, but different baseline treatment. Not controllable.	No block	None 0–24 h	PCA i.v. morphine	Yes	Yes	No	Yes	Yes
Chan, M. H. (2012) [18]	20/21/20/21	Moderate	FNB bupivacaine 0.375% 0.4 mL/kg with epinephrine after spinal anesthesia but before surgical procedure	FNB bupivacaine 0.375% 0.4 mL/kg with epinephrine after completion of surgical procedure	Group 3: Saline after spinal anesthesia but before the surgical procedure. Group 4:Saline after surgical procedure	None (author info)	PCA i.v. morphine	Yes	Yes	Yes	Yes	No
Good, R. P. (2007) [19]	22/20	High	FNB bupivacaine 0.5% 40 mL with epinephrine preoperative	-	Matching saline	-	PCA i.v. morphine	No	No	No	No	No
Hunt, K. J. (2009) [20]	33/24	Moderate	FNB bupivacaine 0.5% 10–15 mL	-	Matching saline	-	PCA i.v. morphine	No	No	No	No	No
Jeong, M. S. (2011) [21]	43/33	Moderate	FNB bupivacaine 0.5% 20 mL and lidocaine 1% 10 mL	-	No block	Periarticular injection of bupivacaine 40 mg / ketorolac 2 mg / morphine 8 mg / epinephrine. Oral celecoxib 200 mg x 2	PCA i.v. fentanyl / ketorolac / zofran and i.v. tramadol	Yes	Yes	No	No	No
Kardash, K. (2007) [22]	19/20/20	Moderate	FNB bupivacaine 0.5% 20 mL with epinephrine	Obturator nerve block bupivacaine 0.5% 20 mL with epinephrine	Sham block: no injection but bandage over the inguinal area	Oral acetaminophen 650 mg x 4. Oral celecoxib 100 mg x 2	PCA i.v. fentanyl and i.m. Ketorolac if VAS > 60	No	Yes	No	Yes	Yes
Lee, A. R. (2011) [23]	38/40	Moderate	FNB bolus levobupivacaine 0.25% 30 mL with epinephrine administered in the PACU	-	No block	-	PCEA ropivacaine / fentanyl and i.v. me-peridine if VAS > 50	Yes	No	Yes	No	No
Ng, H. P. (2001) [24]	12/12/12/12	High	3-in-1 FNB ropivacaine 0.25% 30 mL	Group 2: 3-in-1 FNB ropivacaine 0.5% 30 mL. Group 3: 3-in-1 FNB bupivacaine 0.25% 30 mL	Matching saline	None 0–24 h	PCA i.v. morphine	Yes	Yes	Yes	Yes	Yes
Ozen, M. (2006) [25]	15/15	High	3-in-1 FNB ropivacaine 0.375% 40 mL before general anaesthesia	-	No block	-	PCA i.v. morphine	Yes	Yes	No	No	No
Sahin, L. (2014) [26]	51/53	Moderate	FNB bupivacaine 0.5% 40 mL with epinephrine	-	Matching saline	Oral ibuprofen 600 mg x 3	PCA i.v. morphine	Yes	Yes	Yes	Yes	No
Tugay, N. (2006) [27]	8/7/8	High	FNB bupivacaine 0.25% 40 mL at the start intraoperative	FNB bupivacaine 0.25% 40 mL at the end intraoperative	No block	-	PCA i.v. morphine	Yes	Yes	No	No	Yes
Wang, H. (2002) [28]	15/15	High	FNB bupivacaine 0.25% 40 mL with epinephrine intraoperative	-	Matching saline	-	PCA i.v. morphine	No	Yes	No	Yes	Yes
YaDeau, J. T. (2005) [29]	41/39	Moderate	FNB bupivacaine 0.375% 30 mL with epinephrine intraoperative	-	No block	None 0–24 h	PCEA bupivacaine / hydromorphone	No	No	No	No	No
Hirst, G. C. (1996) [30]	11/11/11	High	Single FNB bupivacaine 0.5% 20 mL with epinephrine	FNB bupivacaine 0.5% 20 mL with epinephrine. Continuous bupivacaine 0.125% 6 mL/h for 48 h	Mock FNB and sham continuous infusion	-	PCA i.v. morphine	No	Yes	No	Yes	No
Edwards, N. D. and E. M. Wright (1992) [31]	19/18	High	3-in-1 FNB bupivacaine 0.25% 30 mL and continuous bupivacaine 0.125% 6mL/h for 24 h	-	No block	-	I.m. papaveretum	Yes	Yes	No	No	No
Ganapathy, S. (1999) [32]	20/20/22	Moderate	FNB bupivacaine 0.1% 30 mL bolus and continuous 10 mL/h for 48 h	FNB bupivacaine 0.2% 30 mL bolus and continuous 10 mL/h for 48 h	Matching saline	Rectal indomethacin 100 mg x 2	PCA i.v. morphine	No	No	No	No	No
Kaloul, I. (2004) [33]	20/20/20	Moderate	FNB ropivacaine 0.5% with epinephrine 30 mL and continuous ropivacaine 0.2% 12 mL/h for 48 h	Psoas compartment nerve block ropivacaine 0.5% with epinephrine 30 mL and continuous ropivacaine 0.2% 12 mL/h for 48 h	No block	Rectal indomethacin 100 mg x 2	PCA i.v. morphine	Yes	Yes	No	No	No
Park, C. K. (2010) [34]	20/20/20/20	Moderate	FNB bupivacaine 0.25% 20 mL with epinephrine and continuous bupivacaine 0.125% 2 mL/h	FNB bupivacaine 0.25% 20 mL with epinephrine and continuous bupivacaine 0.125%: Group 2: 4 mL/h. Group 3: 6 mL/h	FNB bupivacaine 0.25% 20 mL with epinephrine. No continuous FNB	-	PCA i.v. morphine / ketorolac	No	No	No	Yes	No
Seet, E. (2006) [35]	17/18/20	Moderate	Continuous 3-in-1 FNB ropivacaine 0.15% 10 mL/h for 24 h and then 5 mL/h for the next 24 h	Continuous 3-in-1 FNB ropivacaine 0.2% 10 mL/h for 24 h and then 5 mL/h for the next 24 h	No block	Oral acetaminophen 1 g x 4. Oral rofecoxib 50 mg x 1	PCA i.v. morphine	Yes	Yes	Yes	Yes	Yes
Serpell, M. G. (1991) [36]	13/16	High	Continuous lumbar plexus block bupivacaine 0.5% with epinephrine. Bolus 0.3 mL/kg at 6–8 h intervals for 48 h	-	No block	-	PCA i.v. morphine. i.m. morphine, oral paracetamol and NSAIDs p.n.	No	Yes	No	No	No
Watson, M. W. (2005) [37]	16/16	High	Lumbar plexus block levo-bupivacaine 0.1% 15 mL and continuous 10 mL/h for 48 h	-	Matching saline	Sciatic nerve block levobupivacaine 0.5% 15 mL. Lumbar blexus block levobupivacaine 0.5% 25 mL. Oral codeine 16 mg/acetaminophen 1 g x 4. Oral diclofenac 50 mg x 3	PCA i.v. morphine	Yes	Yes	Yes	Yes	Yes
Wyatt, M. C. (2015) [38]	42/42	Moderate	FNB bupivacaine 0.25% 15 mL and continuous bupivacaine 0.125% 0–10 mL/h for 48 h	-	FNB bupivacaine 0.25% 15 mL. Matching saline for continuous FNB	Oral acetaminophen 1 g x 4	I.v. morphine equivalents	Yes	Yes	No	No	Yes
Olive, D. J. (2015) [39]	26/28/27	Moderate	FNB ropivacaine 0.75% 20 mL with epinephrine and continuous 8–14 mL/h until POD 2 morning. Intrathecal morphine 0.175 mg	FNB ropivacaine 0.75% 20 mL with epinephrine and continuous 8–14 mL/h until POD 2 morning. No intrathecal morphine	Intrathecal morphine 0.175 mg. No FNB	Oral acetaminophen 1 g x 4. Oral celecoxib 200 mg x 2	PCA i.v. morphine	Yes	Yes	No	No	No
Barrington, JW. (2016) [40]	41/38	Moderate	Intrathecal morphine 0.2–0.25 mg adjuncts to spinal anesthesia with bupivacaine 0.75% 9 mg	-	Spinal anesthesia with bupivacaine 0.75% 9 mg	I.v. acetaminophen 1 g on induction. Oral Celebrex 200 mg x 1. Preoperative oral oxycontin 20 mg. I.v. dexamethasone 10 mg on induction. Periarticular ropivacaine 0.5% 50 mL, ketorolac 30 mg and epinephrine	Opioids calculated as morphine equivalents (total consumption throughout the study)	Yes	Yes	No	No	Yes
Cole, P. J. (2000) [41]	18/18	High	Spinal morphine 0.3 mg adjunct to bupivacaine	-	Placebo adjunct to bupivacaine	Oral diclofenac 50 mg x 3	PCA i.v. morphine	No	No	Yes	Yes	No
Hur, M. J. (2007) [42]	16/18/20	Moderate	Intrathecal morphine 50 microgram	Intrathecal morphine 100 microgram	No intrathecal treatment	Not described	PCEA levobupi-vacaine / fentanyl and oral ketorolac if VAS > 30	Yes	Yes	Yes	Yes	No
Jacobson, L. (1989) [43]	7/7/7/7/7	High	Intrathecal diamorphine 0.25 mg adjunct to spinal bupivacaine 0.5% 15 mg	Adjunct to spinal bupivacaine 0.5% 15 mg: Intrathecal diamorphine. Group 2: 0.75 mg. Group 3: 1.5 mg. Group 4: 2.5 mg	Spinal bupivacaine 0.5% 15 mg	-	I.m. morphine p.n.	Yes	Yes	No	No	No
Kunopart, M. (2014) [44]	15/15/15/15	Moderate	Intrathecal morphine sulfate 0.1 mg adjuncts to spinal anesthesia with bupivacaine 0.5% 15 mg	Adjunct to spinal anesthesia with bupivacaine 0.5% 15 mg: Intrathecal morphine. Group 2: 0.2 mg. Group 3: 0.3 mg	Spinal bupivacaine 0.5% 15 mg	-	PCA i.v. morphine	Yes	Yes	No	No	No
Lauretti, G. R. (2013) [45]	19/20/19/18	Moderate	Intrathecal morphine 0.2 mg and bupivacaine 15 mg	Group 2: Intrathecal ketorolac 2 mg adjunct to bupivacaine 15 mg. Group 3: Intrathecal morphine 0.2 mg and ketorolac 2 mg adjunct to bupivacaine 15 mg	Intrathecal bupivacaine 15 mg	-	I.v. ketoprofen p.n. and tramadol i.v.	No	No	No	Yes	No
Park, C. K. (2009) [46]	20/20/20/20/ 20	Moderate	Intrathecal 0.05 mg morphine adjunct to bupivacaine 0.5% 6–13 mg with epinephrine	Group 2: Intrathecal morphine 0.1 mg. Group 3: Intrathecal morphine 0.15 mg adjunct to bupivacaine 0.5% 6–13 mg with epinephrine	Intrathecal bupivacaine 0.5% 6–13 mg with epinephrine	3-in-1 femoral nerve block bupivacaine 0.25% / epinephrine 20 mL and continuous bupivacaine 0.125% 2 mL/h. I.m. diclofenac 90 mg x 2	PCA continuous FNB, i.m. diclofenac and i.m. butorphanol if VAS > 50	Yes	Yes	Yes	Yes	No
Tan, P. H. (2001) [47]	20/20/20	Moderate	Intrathecal morphine 0.3 mg and bupivacaine 0.5% 3 mL	Intrathecal bupivacaine 0.5% 3 mL and neostigmine 50 microgram	Intrathecal bupvacaine 0.5% 3 mL and saline	-	I.m. diclofenac p.n. if VAS > 4i	Yes	Yes	No	No	No
Busch, C. A. (2006) [48]	32/32	Moderate	LIA ropivacaine 400 mg, ketorolac 30 mg, epimorphine 5 mg and epinephrine	-	No injection	No description	PCA i.v. morphine	No	No	Yes	Yes	Yes
Chinachoti, T. (2012) [49]	50/49	Moderate	Intraoperative LIA bupivacaine 0.25% 20 ml	-	Placebo	Oral acetaminophen 1 g x 4. I.v. parecoxib 40 mg x 2. I.v. etoricoxib 120 mg x 1. Femoral nerve block bupivacaine 0.25% 20 mL	PCA i.v. morphine	Yes	Yes	No	No	No
Choi, H. G. (2006) [50]	20/20	High	LIA bupivacaine 150 mg and morphine 10 mg	-	Matching saline	Continuous epidural infusion of bupivacaine 150 mg / morphine 5 mg 0–48 h	I.m. diclofenac p.n.	Yes	Yes	No	No	No
Essving, P. (2010) [51]	24/23	High	LIA ropivacaine 400 mg, ketorolac 30 mg and epinephrine 0.5 mg during operation. At 21 h: intraarticular ropivacaine 200 mg, ketorolac 30 mg and epinephrine 0.1 mg during operation.	-	No LIA during surgery. At 21 h: saline injection intraarticularly	Oral acetaminophen 1 g x 4	PCA i.v. morphine mg	Yes	Yes	Yes	Yes	Yes
Fu, P. (2009) [52]	40/40	Moderate	LIA bupivacaine 30 mg, morphine 5 mg and betamethasone 1 mL	-	Saline	Celecoxib 200 mg	PCA i.v. morphine mg	Yes	Yes	No	Yes	No
Kazak Bengisun, Z. (2010) [53]	20/20/20	Moderate	Intraoperative LIA bupivacaine 200 mg and epinephrine 0.5 mg in 150 mL. And 120 mg bupivacaine with epinephrine bolus at 10 and 22 h	Intraoperative LIA levobupivacaine 200 mg and epinephrine 0.5 mg in 150 mL. And 120 mg levobupivacaine with epinephrine bolus at 10 and 22 h	Matching saline	-	PCA tramadol and diclofenac if VAS > 50	Yes	Yes	Yes	Yes	Yes
Kim, T. W. (2015) [54]	43/43/42/43/43/42	Low (200–499 patients)	LIA ropivacaine 180 mg	LIA ropivacaine 180 mg and morphine 5 mg	Matching saline	I.v. ketorolac 30 mg x 3. Fentanyl patch 25 mikrogram / third day	PCA i.v. fentanyl or i.m pethidine	Yes	Yes	No	No	No
Leownorasate, M. (2014) [55]	21/21	High	LIA levopubivacaine 100 mg, diclofenac 75 mg, morphine 5 mg and epinephrine	-	No injection	None (author info)	PCA i.v. morphine	Yes	Yes	No	No	Yes
Lu, H. H. (2014) [56]	15/15	High	LIA ropivacaine 300 mg, morphine 5 mg and epinephrine 10 microgram	-	Matching saline	Epidural lidocaine 0.1% 5 mL/h. Oral celebrex 200 mg x 2 or if oral intake not possible pericoxib i.v. 40 mg x 2	-	Yes	Yes	Yes	Yes	No
Milani, P. (2015) [57]	32/30	Moderate	LIA ropivacaine 1% 20 mL	-	Matching saline	Etoricoxib 90 mg x 1. Oxycodone/naloxone 20/10 mg x 2	I.m. ketorolac 10 p.n. if VAS > 4	Yes	No	No	No	No
Niemelainen, M. (2014) [58]	27/29	High	LIA levopubivacaine 150 mg, ketorolac 30 mg and epinephrine 0.5 mg	-	Matching saline	Oral acetaminophen 1 g x 4. Oral meloxicam 15 mg x 1 (2 h after surgery)	PCA i.v. oxycodone	No	No	No	No	No
Ong, J. C. (2010) [59]	16/21/17	Moderate	Continuous LIA bupivacaine 0.25% 4 mL	LIA bupivacaine 100 mg, morphine 10 mg and ketorolac 1 mL. Continuous LIA bupivacaine 0.25% 4 mL	No injection	None (author info)	PCA i.v. morphine	Yes	Yes	No	No	Yes
Vaishya, R. (2016) [60]	40/40	Moderate	LIA bupivacaine 0.25% 20 mL, morphine 15 mg, ketorolac 30 mg, epinephrine. Total dose 75 mL	-	Placebo	I.v. paracetamol 1 g x 3. I.v. diclofenac 75 mg x 2	PCA i.v. Morphine	Yes	Yes	Yes	Yes	Yes
Vendittoli, P. A. (2006) [61]	22/20	High	LIA ropivacaine 275 mg, ketorolac 30 mg and epinephrine continued by ropivacaine 125 mg at wound closure	-	No injection	Acetaminophen 500 mg x 4. Celecoxib 200 mg x 2	PCA i.v. morphine	No	No	No	No	Yes
Yuenyongviwat, V. (2012) [62]	30/30	Moderate	LIA bupivacaine 0.25% 20 mL before wound closure	-	Matching saline	Oral acetaminophen 1 g x 4. Oral meloxicam 7.5 mg x 2	PCA i.v. morphine	No	No	No	No	Yes
Zhang, J. (2007) [63]	30/30	Moderate	LIA bupivacaine 0.5% 30 mL, morphine 10 mg and epinephrine	-	No injection	I.v. lornoxicam 0.3 mg/h for 48 h	I.m. morphine p.n. PCA tramadol 500 mg	Yes	Yes	Yes	Yes	No
Zhang, S. (2011) [64]	27/27/26	Moderate	Single-injection LIA ropivacaine 300 mg and ketorolac 30 mg. Continuous saline	Single-injection LIA ropivacaine 300 mg and ketorolac 30 mg. Continuous ropivacaine 8 mg/h and ketorolac 1.25 mg/h for 48 h	Matching saline	Oral celecoxib 200 mg x 2	PCA i.v. morphine	Yes	Yes	Yes	Yes	No
Nakai, T. (2013) [65]	21/19/20	Moderate	Intraarticular injection bupivacaine 0.5% 20 mL, morphine 10 mg and epinephrine 0.3 mg	LIA ropivacaine 0.75% 30 mL, morphine 10 mg, betamethasone 4 mg and epinephrine 0.25 mg	No injection	No description	Suppository diclofenac	Yes	Yes	No	No	No
Badner, N. H. (1996) [66]	28/27/27	Moderate	Intraarticular injection bupivacaine 0.5% 30 mL with epinephrine before skin incision. Intraarticular saline after wound closure.	Intraarticular saline before skin incision. Intraarticular bupivacaine 0.5% 30 mL with epinephrine after wound closure.	Intraarticular saline before skin incision. Intraarticular saline after wound closure.	No description	PCA i.v. morphine	No	Yes	No	No	No
Browne, C. (2004) [67]	30/30	Moderate	Intraarticular injection bupivacaine 0.5% 20 mL with epinephrine after closure.	-	Intraarticular injection saline 20 mL with epinephrine after closure.	No description	Opioids calculated as morphine equivalents	No	No	No	No	No
Mauerhan, D. R. (1997) [68]	26/24/28/27	Moderate	Intraarticular injection morphine 5 mg	Intraarticular injection: Group 2: Bupivacaine 50 mg. Group 3: Bupivacaine 50 mg and morphine 5 mg	Matching saline	None (author info)	PCA i.v. morphine or meperidine	Yes	Yes	No	No	No
Rosen, A. S. (2010) [69]	24/24	High	Intraarticular ropivacaine 0.2% 100 mL after closure	-	Matching saline	-	PCA i.v. morphine and other narcotics converted to i.v. morphine equivalents	Yes	Yes	No	No	No
Safa, B. (2014) [70]	33/32/35	Moderate	Sciatic nerve block with ropivacaine 0.5% 20 mL preoperatively. Posterior capsule injection of saline at the end of surgery.	Sciatic nerve block with saline. Posterior capsule injection with ropivacaine 0.2% 20 mL at the end of surgery.	Sciatic nerve block with saline. Posterior capsule injection with saline at the end of surgery.	FNB ropivacaine 0.5% 20 mL. Oral acetaminophen 1 g x 4. Oral celecoxib 200 mg x 2. Oral gabapentin 200 mg x 3	PCA i.v. hydromorphone	Yes	Yes	Yes	Yes	Yes
Shen, S. J. (2015) [71]	20/16	High	Intraarticular bupivacaine 0.5% 60 mL	-	Matching saline	I.v. parecoxib 40 mg preoperative. None postoperative	I.m. meperidine p.n.	Yes	Yes	No	No	No
Feng, Y. (2004) [72]	15/15	High	Oral rofecoxib 25 mg 1 h prior to surgery	-	Matching placebo	Not described	PCEA morphine / bupivacaine / droperidol	No	No	No	No	No
Huang, Y. M. (2008) [73]	40/40	Moderate	Oral celecoxib 400 mg 1 h preoperative and 200 mg x 2 daily	-	No capsules	-	PCA i.v. morphine	Yes	Yes	No	Yes	No
Hubbard, R. C. (2003) [74]	61/65/63	Moderate	I.v. parecoxib 20 mg x 2	I.v. parecoxib 40 mg x 2	Matching placebo	-	PCA i.v. morphine	Yes	Yes	No	No	No
Inan, N. (2007) [75]	20/20	High	I.v. lornoxicam 16 mg before surgery and 8 mg x 2 daily	-	Matching saline	-	PCA i.v. morphine	Yes	Yes	No	No	No
Rawal, N. (2013) [76]	222/230/223/98	Very low (>499 patients)	Oral etoricoxib 90 mg x 1 and matching placebo x 2	Group 2: Oral etoricoxib 120 mg x 1 and matching placebo x 2. Group 3: Oral ibuprofen 600 mg x 3	Matching placebo	-	PCA i.v. morphine	No	No	No	No	No
Sarridou, D. G. (2015) [77]	45/45	Moderate	I.v. parecoxib 40 mg x 2	-	Matching placebo	FNB ropivacaine 0.75% 20 mL and continuous 0.2% 10 mL/h	PCA i.v. morphine	Yes	Yes	No	No	No
Silvanto, M. (2002) [78]	24/24/16	Moderate	I.v. diclofenac 75 mg in PACU and oral diclofenac 50 mg x 3 daily	I.v. ketoprofen 100 mg in PACU and oral keotoprofen 100 mg x 3 daily	Matching placebo	-	PCA i.v. oxycodone	Yes	No	No	No	No
Zhu, Y. (2014) [79]	50/50	Moderate	I.v. parecoxib 40 mg x 2	-	Matching saline	Periarticular injection morphine 4 mg / ropivacaine 35 mg / epinephrine. Postoperative not described	I.v. morphine p.n. if VAS > 40	Yes	Yes	No	Yes	No
Zhu, YZ. (2016) [80]	60/62	Moderate	I.v. parecoxib 40 mg x 2	-	Placebo	No description	PCA fentanyl	Yes	Yes	No	No	Yes
Niruthisard, S. (2013) [81]	25/22/22/25	Moderate	Oral pregabalin 150 mg and placebo the morning of surgery	At the morning of surgery: Group 2: Oral celecoxib 400 mg and placebo. Group 3: Oral pregabalin 150 mg and celecoxib 400 mg	Matching placebo	-	PCA i.v. morphine	Yes	Yes	Yes	Yes	No
Clarke, H.A. (2009) [82]	7/8/7/7/7	High	Oral gabapentin 600 mg preoperative and placebo postoperative	Group 2: Oral gabapentin 600 mg preoperative and 100 mg postoperative. Group 3: Oral gabapentin 600 mg preoperative and 200 mg postoperative. Group 4: Oral gabapentin 600 mg preoperative and 300 mg postoperative	Matching placebo	Femoral and sciatic nerve block ropivacaine 0.5% 20 mL each preoperative. Oral celecoxib 200 mg x 2	PCA i.v. morphine	No	Yes	No	No	No
Clarke, H. A. (2014) [83]	88/77	Moderate	Oral gabapentin 600 mg 2 h before surgery and 200 mg x 3 postoperative	-	Matching placebo	Femoral and sciatic nerve block ropivacaine 0.5% 20 mL each preoperative. Oral celecoxib 200 mg x 2	PCA i.v. morphine	No	No	No	Yes	No
Lee, J. K. (2014) [84]	21/20	High	Oral pregabalin 150 mg 1 h before operation	-	None	Periarticular injection bupivacaine 0.5% 10 mL / morphine 5 mg / epinephrine / methylprednisolone 1 mL. Oral celecoxib 200 mg x 2	PCA i.v. fentanyl and i.m. tramadol if VAS > 40	Yes	Yes	Yes	Yes	No
Lunn, T. H. (2015) [85]	91/92/91	Low	Oral gabapentin cumulated 1300 mg daily divided in 4 doses	Oral gabapentin cumulated 900 mg daily divided in 3 doses + 1 placebo	Matching placebo	LIA ropivacaine 0.2% with epinephrine. Oral slow release acetaminophen 2 g x 2. Oral celecoxib 200 mg x 2	Oral morphine equivalents	Yes	Yes	Yes	Yes	Yes
Paul, J. E. (2013) [86]	52/49	Moderate	Oral gabapentin 600 mg preoperatively and 200 mg x 3 postoperative	-	Matching placebo	Oral acetaminophen 1 g x 4. Oral ketorolac 15 mg x 4	PCA i.v. morphine	No	No	No	No	Yes
YaDeau, J. T. (2015) [87]	28/29/29/29	Moderate	Oral pregabalin 50 mg two capsules before operation and 1 capsule x 2 postoperative	Oral pregabalin two capsules before operation and 1 capsule x 2 postoperative. Group 2: 100 mg capsules. Group 3: 150 mg capsules	Matching placebo	FNB bupivacaine 0.25% 30 mL / epinephrine. Oral dexamethasone 6 mg preoperative. Oral meloxicam 7.5–15 mg	PCEA bupivacaine / hydromorphine. Oral oxycodone-paracetamol 5 mg / 325 mg p.n.	No	Yes	No	No	Yes
Andersen, H. L. (2013) [88]	20/20	High	Saphenous nerve block ropivacaine 0.75% 15 mL administered 3 times: in the PACU, 8:00 PM and 8:00 AM	-	Matching saline	LIA single-dose 100mL ropivacaine 2 mg/mL and epinephrine. Dexamethasone 8 mg preoperative. Acetaminophen 1 g x 4. Oral extended release morphine 10 mg x 2	PCA i.v. morphine and FNB boluses of ropivacaine	No	Yes	No	No	Yes
Jenstrup, M. T. (2012) [89]	34/37	Moderate	Adductor canal block ropivacaine 0.75% 30 ml immediately postoperative and additional 15 ml at 6, 12 and 18 h postoperatively.	-	Matching saline	Oral acetaminophen 1 g x 4. Oral ibuprofen 400 mg x 4	PCA i.v. morphine	Yes	Yes	Yes	Yes	No
Krishnan, SH. (2016) [90]	48/49	Moderate	Adductor canal block bupivacaine 30 mL 0.25% and buprenorphine 0.2 mg	-	Adductor canal block bupivacaine 30 mL 0.25%	No description	Oral hydrocodone equivalents	No	No	No	No	No
Shah, N. A. (2015) [91]	46/39	Moderate	Adductor canal block ropivacaine 0.75% 30 mL and continuous ropivacaine 0.25% 30 mL every 4 h 0–24 h	-	Adductor canal block ropivacaine 0.75% 30 mL and continuous matching saline	Intraarticular infiltration sensorcaine 0.25% 20 mL. Oral acetaminophen 500 mg x 4. I.v. diclofenac 75 mg x 3	I.m. tramadol 50 mg for breakthrough pain	Yes	Yes	No	No	Yes
Casati, A. (2005) [92]	20/19/19	Moderate	FNB ropivacaine 0.75% 25 mL and clonidine 1 microgram/kg. Continuous ropivacaine 0.2%	FNB bolus ropivacaine 0.75% 25 mL and clonidine 1 microgram/kg. Continuous ropivacaine 0.2% and clonidine 1 microgram/mL	FNB ropivacaine 0.75% 25 mL. Continuous ropivacaine 0.2%	Sciatic nerve block 0.75% 15 mL. Ketoprofen 10 mg i.v. x 3	PCA continuous FNB and i.v. fentanyl	Yes	Yes	Yes	Yes	No
Ekmekci, P. (2010) [93]	20/20/20	Moderate	Continuous FNB ropivacaine 0.2% and tramadol 1 mg/mL 0.1 mL/h for 48 h	Continuous FNB with ropivacaine 0.2% and tramadol 2 mg/mL 0.1 mL/h for 48 h	Continuous FNB with ropivacaine 0.2% 0.1 mL/h for 48 h	FNB ropivacaine 0.5% 0.3 mL/kg. I.m. meperidine 25 mg preoperative	I.m. diclofenac if VAS > 40	Yes	Yes	No	No	No
Elmawgoud, A. A. (2008) [94]	20/20/20	Moderate	FNB ropivacaine 0.2% 30 mL with fentanyl 4 mikrog/mL and continuous 6 mL/h for 24 h	FNB ropivacaine 0.2% 30 mL and magnesium sulphate 50 mg/mL and continuous 6 mL/h for 24 h	FNB ropivacaine 0.2% 30 mL and continuous 6 mL/h for 24 h	-	PCA i.v. morphine	Yes	Yes	No	No	No
Kosel, J. (2015) [95]	28/20	High	FNB bupivacaine 0.25% with epinephrine 0.5 mL/kg and buprenorphine 0.3 mg	-	FNB bupivacaine 0.25% with epinephrine 0.5 mL/kg	None	I.m. morphine p.n., tramadol p.n., acet-aminophen p.n. and ketoprophene p.n.	No	Yes	No	No	Yes
McNamee, D. A. (2001) [96]	25/25/24	Moderate	Combined femoral and sciatic block with bupivacaine 2 mg/kg divided equally between femoral and sciatic nerves	Combined femoral and sciatic block with ropivacaine 2 mg/kg divided equally between femoral and sciatic nerves	No peripheral nerve block but area prepared and dressing applied to the appropriate sites	-	PCA i.v. morphine	Yes	Yes	No	No	No
Abdallah, F. W. (2014) [97]	17/18/18	Moderate	Proximal sciatic nerve block at infragluteal level of 2:1 bupivacaine 0.5% and lidocaine 2% 30 mL with epinephrine. Distal sham 1 mL saline	Distal sciatic nerve block at popliteal level of 2:1 bupivacaine 0.5% and lidocaine 2%30 mL with epinephrine. Proximal sham 1 mL	Sham injection 1 mL each location	Continuous FNB ropivacaine 0.2% bolus with epinephrine 20 mL and infusion at 5 mL/h. Acetaminophen 1 g x 4. Celecoxib 200 mg x 2. Oxycodone-controlled release 10 mg x 3	PCA continuous FNB i.v. fentanyl p.n., oral oxycodone p.n., PCA i.v. hydro-morphine if NRS > 60	Yes	Yes	Yes	Yes	No
Cappelleri, G. (2011) [98]	19/18	High	Continuous sciatic nerve block levobupivacaine 0.06% 0.1 mL/kg. Continuous lumbar plexus block levobupivacaine 0.125% 8 mL/h	-	Continuous sciatic nerve block saline 0.1 mL/kg. Continuous lumbar plexus block levobupivacaine 0.125% 8 mL/h	I.v. ketorolac 30 mg x 3	PCA i.v. morphine	Yes	Yes	Yes	Yes	No
Martinez Navas, A. and M. Echevarria Moreno (2006) [99]	7/10	High	Sciatic nerve block ropivacaine 0.5% 20 mL. Continuous ropivacaine 0.2% 5 mL/h	-	Sciatic nerve block ropivacaine 0.5% 20 mL	FNB Ropivacaine 0.2% 0,4 ml/kg and continuous 5 ml/h + PCA boluses. I.v. acetaminophen 1 g x 4. I.m. diclofenac 50 mg x 2	S.c. morphine-chloride p.n.	Yes	Yes	Yes	Yes	No
Sato, K. (2014) [100]	30/30	Moderate	Sciatic nerve block ropivacaine 0.2% 20 mL. Continuous ropivacaine 0.2% 5 mL/h	-	Continuous sciatic nerve block ropivacaine 0.2% 20 mL. Continuous saline	FNB ropivacaine 0.5% 20 mL and continuous ropivacaine 0.2% 5 mL/h. Oral loxoprofen 60 mg x 3	PCA i.v. morphine	Yes	Yes	No	No	Yes
McNamee, D. A. (2002) [101]	24/27	Moderate	Obturator nerve block ropivacaine 0.75% 5 mL. Femoral and sciatic nerve block ropivacaine 0.75%15 mL to each nerve	-	Femoral and sciatic nerve block ropivacaine 0.75% 15 mL to each nerve	Ranitidine 150 mg 1 h preoperative. None postoperative	PCA i.v. morphine	No	No	Yes	Yes	No
Runge, C. (2016) [102]	23/26	High	Obturator nerve block bupivacaine 46 mg, clonidine 0.0375 mg, dexamethasone 2 mg, epinephrine	-	No block	Femoral triangle block bupivacaine 46 mg, clonidine 0.0375 mg, dexamethasone 2 mg, epinephrine	PCA i.v. morphine	Yes	Yes	Yes	Yes	Yes
Frassanito, L. (2009) [103]	22/22	High	Single lumbar plexus block ropivacaine 0.6% 30 mL. Single sciatic block ropivacaine 0.6% 15 mL. Continuous lumbar plexus infusion of ropivacaine 0.2% 10 mL/h for 48 h	-	Single lumbar plexus block ropivacaine 0.6% 30 mL. Single sciatic nerve block ropivacaine 0.6% 15 mL	Fentanyl i.v. 50 mikrog preoperative. I.v. acetaminophen 1 g x 4	I.v. tramadol p.n. if VAS > 40 mm	Yes	Yes	No	No	No
Badner, N. H. (1997) [104]	25/26/24	Moderate	Intraarticular bupivacaine 0.5% 30 mL and morphine 1 mg with epinephrine	Intraarticular saline 30 mL and morphine 1 mg with epinephrine	Intraarticular bupivacaine 0.5% 30 mL with epinephrine and 1 mL saline	No description	PCA i.v. morphine	Yes	Yes	No	No	No
Garcia, J. B. (2010) [105]	25/25	Moderate	Intraarticular 10 mg morphine in 20 mL	-	Matching saline	-	S.c. morphine p.n.	Yes	Yes	No	No	No
Guara Sobrinho, H. (2012) [106]	19/17/20	Moderate	Intraarticular ketamine 0.25 mg/kg in 20 mL just before complete closure of the skin	Intraarticular ketamine 0.5 mg/kg in 20 mL	Intraarticular saline 20 mL	-	I.v. morphine pn	Yes	Yes	No	No	No
Schotanus, M. G. (2015) [107]	25/25	Moderate	Intracapsular LIA ropivacaine 2% 150 mL. 100 mL of these with epinephrine 0.01%	-	Intracapsular LIA ropivacaine 2% 150 mL	Oral acetaminophen 1 g x 2. Oral etoricoxib 90 mg x 1. Oral gabapentin 300 mg x 1	Tramadol p.n.	Yes	Yes	No	No	Yes
Ali, A. (2015) [108]	97/95	Moderate	Continuous intraarticular infusion of ropivacaine 15 mg/h for 48 h	-	Matching saline	Periarticular injection ropivacaine 300 mg / ketorolac 30 mg / epinephrine. Oral acetaminophen 1 g x 4. Oral diclofenac 25 mg x 3. Patch buprenorphine 10 mikrogram/h	Oxycodone p.n.	Yes	Yes	No	No	Yes
Gomez-Cardero, P. and E. C. Rodriguez-Merchan (2010) [109]	25/25	Moderate	Continuous intraarticular infusion of ropivacaine 0.2% 5 mL/h, cumulated 300 mL	-	Matching saline	Oral acetaminophen 1 g x 4. I.v. ketorolac 10 mg x 3	I.v. morphine or s.c. pethidine p.n.	No	Yes	No	No	Yes
Williams, D. (2013) [110]	24/25	High	Continuous intraarticular infusion of bupivacaine 0.5% 2 mL/h for 48 h	-	Matching saline	Oral acetaminophen 650 mg x 6. I.v. ketorolac 15 mg x 4. Gabapentin 300 mg x 2. Oxycodone 10 mg x 2	PCA i.v. morphine	Yes	Yes	No	No	Yes
Andersen, K. V. (2013) [111]	30/30	Moderate	Intraoperative LIA ropivacaine 300 mg and ketorolac 30 mg. Postoperative intraarticular ropivacaine 100 mg and ketorolac 15 mg every 6h	-	Intraoperative LIA ropivacaine 300 mg and saline. Postoperative intraarticular ropivacaine 100 mg and ketorolac 15 mg every 6h	Oral acetaminophen 1 g x 4	PCA i.v. morphine	Yes	Yes	Yes	Yes	Yes
Sean, V. W. (2011) [112]	50/50	Moderate	Periarticular bupivacaine 0.5% 0.5 mL/kg with epinephrine half in deep tissue and half in skin at closure. In deep tissue triamcinolone acetonide (corticosteroid) 40 mg was added	-	Periarticular bupivacaine 0.5% 0.5 mL/kg with epinephrine half in deep tissue and half in skin at closure	Oral naproxen, unclear dose	PCA i.v. morphine	Yes	Yes	No	No	Yes
Tsukada, S. (2016) [113]	38/37	Moderate	Periarticular ropivacaine 300 mg with morphine 8 mg, ketoprofen 50 mg, epinephrine and methylprednisolone 40 mg	-	Periarticular ropivacaine 300 mg with morphine 8 mg, ketoprofen 50 mg, epinephrine	I.v. Flurbiprofen 50 mg four h after spinal anaesthesia.	Suppository diclofenac	Yes	Yes	No	No	No
Yue, D. B. (2013) [114]	36/36	Moderate	Periarticular ropivacaine 0.75% 30 mL with epinephrine and betamethasone 1 mL	-	Periarticular ropivacaine 0.75% 30 mL with epinephrine	Oral celecoxib 200 mg regularly	PCA i.v. morphine	Yes	No	No	No	No
Axelsson, K. (2005) [115]	15/15/15	High	Low dose: Epidural initiated in the PACU: ropivacaine 1.25 mg/mL + morphine 0.02 mg/mL, 8 mL/h	High dose: Epidural initiated in the PACU: ropivacaine 2 mg/mL + morphine 0.02 mg/mL, 8 mL/h	Matching saline	Oral acetaminophen 1 g preoperative. I.m. oxycodon 0.1 mg/kg preoperative. No description of postoperative	PCA i.v. morphine	Yes	Yes	No	Yes	No
Daabiss, M. A. and A. Kandil (2013) [116]	40/40/40	Moderate	Epidural bolus bupivacaine 0.5% 1 mL and magnesium sulphate 50 mg. And intraoperative epidural infusion magnesium sulphate 10 mg/h	Epidural bolus bupivacaine 0.5% 1 mL and midazolam 0.05 mg/kg. And intraoperative epidural saline infusion	Epidural bolus bupivacaine 0.5% 1 mL and saline. And intraoperative epidural saline infusion	-	PCEA fentanyl and i.m. pethidine if VAS > 30	Yes	Yes	No	No	No
Hendolin, H. (1996) [117]	10/10/10/11	High	I.m. morphine 0.14 mg/kg 1 h preoperative. Epidural morphine 4 mg at 0 h and 3 mg at 10 h postoperative	Group 2: I.m. saline 1 h preoperative. Epidural morphine 4 mg at 0 h and 3 mg at 10 h postoperative. Group 3: I.m. morphine 0.14 mg/kg 1 h preoperative. Epidural saline 0 and 10 h postoperative	Matching i.m. and epidural saline	None (author info)	PCA i.v. fentanyl	Yes	Yes	No	No	No
Abrisham, S. M. (2014) [118]	20/20	High	Transdermal fentanyl patch 4.2 mg/patch	-	Placebo patches	None	PCA i.v. morphine	Yes	Yes	No	No	No
Sathitkarnmanee, T. (2014) [119]	20/20	High	Transdermal fentanyl patch 50 microgram/h constituted 10–12 h before surgery	-	Placebo patch	None (author info)	PCA i.v. morphine	Yes	Yes	Yes	No	No
Stiller, C. O. (2007) [120]	22/28	Moderate	I.v. tramadol 100 mg x 4	-	Matching saline	Oral acetaminophen 1 g x 4	PCA i.v. morphine	Yes	No	No	No	No
Aveline, C. (2009) [121]	24/25/24	Moderate	I.v. nefopam 0.2 mg/kg bolus after anaesthetic induction and 0.12 mg/kg/h until the end of surgery followed by 0.06 mg/kg/h until POD2	I.v. ketamine 0.2 mg/kg bolus after anaesthetic induction and 0.12 mg/kg/h until the end of surgery followed by 0.06 mg/kg/h until POD2	Placebo	None	PCA i.v. morphine	Yes	Yes	Yes	Yes	Yes
Adam, F. (2005) [122]	20/20	High	Ketamine 0.5 mg/kg bolus followed by 0.003 mg/kg/min during surgery and 0.0015 mg/kg/min after surgery	-	Placebo	FNB ropivacaine 0.75% 0.3 mL/kg and continuous ropivacaine 0.2% 0.1 mL/kg/h	PCA i.v. morphine	Yes	Yes	No	No	Yes
Cengiz, P. (2014) [123]	30/30	Moderate	Intraoperative i.v. ketamine 6 microgram/kg/minute until closure	-	Placebo	I.v. acetaminophen 1 g x 3	PCA i.v. morphine	Yes	Yes	No	No	No
Casey, G. (2006) [124]	20/20	High	Oral nimodipine 90 mg 1 h preoperative and 30 mg x 4 postoperative	-	Placebo	Oral acetaminophen x 4 unknown dose	PCA i.v. morphine	Yes	Yes	Yes	Yes	No
Chan IA. (2016) [125]	20/20	High	I.v. dexmedetomidine 0.5 microg/kg bolus and 0.5 microg/kg/h infusion during surgery	-	Placebo	None	PCA i.v. morphine	Yes	Yes	No	No	No
Ho, K. Y. (2010) [126]	23/24	High	Oral duloxetine 60 mg 2 h before surgery and the morning of POD1	-	Placebo	Acetaminophen 1 g x 4	PCA i.v. morphine	Yes	Yes	Yes	Yes	No
Lunn, T. H. (2011) [127]	24/24	High	I.v. solu-medrol 125 mg just before spinal anesthesia	-	Matching saline	Oral slow-release acetaminophen 2 g x 2. Oral celecoxib 200 mg x 2. Oral gabapentin 300 mg morning + 600 mg evening	I.v. sufentanil and oral oxycodone p.n.	Yes	Yes	Yes	Yes	Yes
Frassanito, L. (2015) [128]	20/20	High	I.v. magnesium 40 mg/kg bolus and 10 mg/kg/h during surgery	-	Placebo	I.v. fentanyl 50 microgram preoperative. I.v. acetaminophen 1 g x 4. I.v. ketorolac 30 mg x 2	PCA i.v. morphine	No	No	No	No	No

FNB: femoral nerve block. LIA: local infiltration analgesia. PACU: postanaesthetic care unit. PCA: patient controlled analgesia. POD1: postoperative day 1. PONV: postoperative nausea and vomiting.

### Risk of bias in included trials

105 trials contained at least one unclear domain (a total of 350 unclear domains). We contacted the corresponding authors by email. Email addresses were either irretrievable or permanently out of use in 22 trials. Corresponding authors for the remaining 83 trials were contacted. Forty authors replied regarding 119 unclear domains and 74 were resolved (5 high and 69 low). Forty-four domains remained unclear.

**Fig 2:**
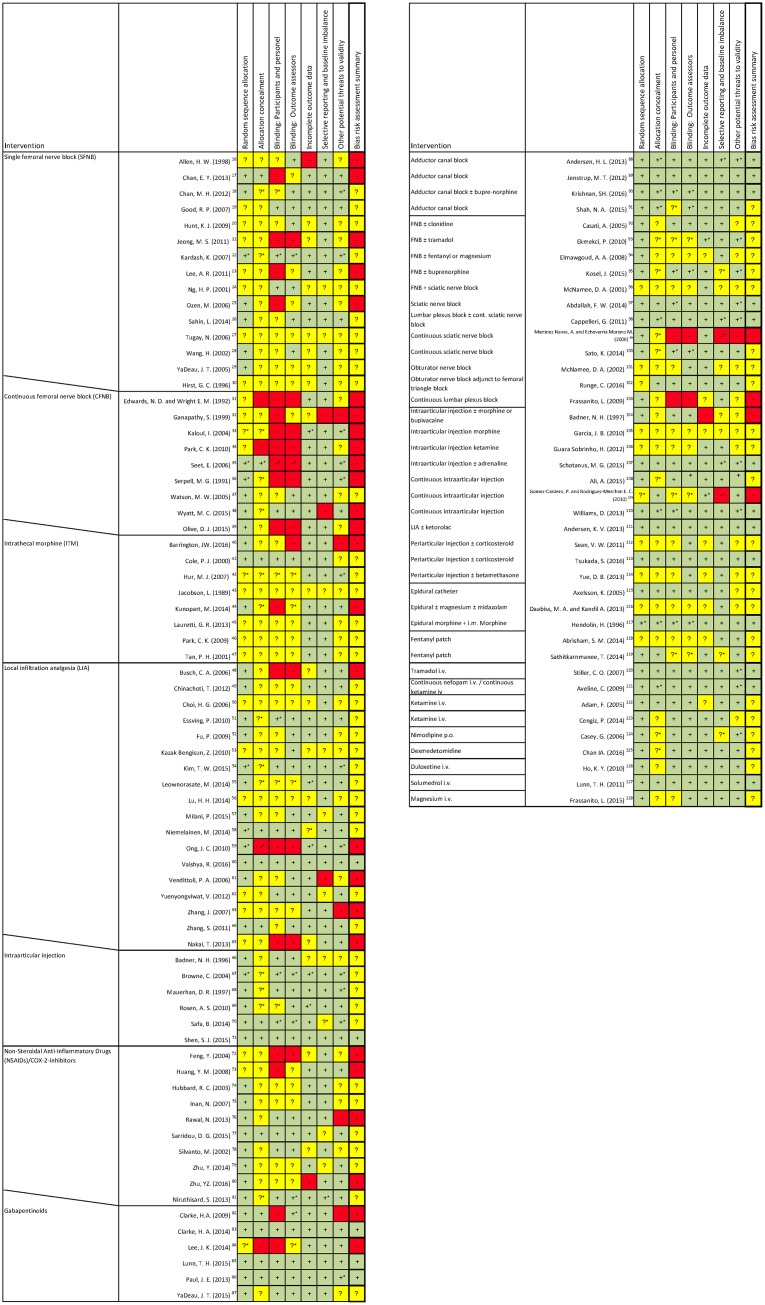
Risk of bias in included studies. Green plus is low risk, yellow question mark is unclear risk, and red minus is high risk of bias. Slanted lines indicate that the trial is part of both surrounding subgroups. * Indicates that information regarding the bias domain has been reevaluated after obtaining an elaboration from the corresponding author of the trial.

The summarized risk of bias was low in 18 trials, unclear in 65 and high in 30 (Fig 2). Further, the trial sample size bias was high in 41 trials, moderate in 69, and low or lower in three.

### Supplemental and basic analgesic regimens

Sixty trials administered i.v. morphine patient-controlled analgesia (PCA) as rescue medication, and reported a 0–24 hours cumulated consumption, while the remaining 53 trials administered i.v./i.m. fentanyl, oxycodone, hydromorphone, meperidine, papaveretum (a mixture of morphine, papaverine, and codeine), sufentanil or NSAIDs; patient-controlled continuous FNB; or epidural local anaesthetics/opioids. Eighty-nine trials reported cumulated opioid consumption over 20–72 hours postoperatively, seven of these also administered a second non-opioid rescue analgesic. In five trials included in meta-analyses, other types of opioids were converted to i.v. morphine equivalents. Postoperative 0–24 hours morphine consumption in the control groups for trials included in the meta-analyses ranged from 5.5–116 mg with a corrected mean of 33.1 mg per patient.

A supplemental opioid with no underlying basic analgesic regimen, was administered in 37 trials. Sixty-three trials administered a basic analgesic regimen in addition to supplemental rescue analgesics; seven trials administered acetaminophen, 13 trials NSAIDs, 12 trials acetaminophen + NSAID, seven trials local injection + other analgesics, 15 trials nerve blocks + other analgesics, and 11 trials administered different combinations of analgesics (Table 1).

### Pain scores

Pain score was reported as VAS 0–100 in 42 trials; as VAS 0–10 in 52 trials; and as either numerical rating scale 0–10 (NRS 0–10), WOMAC pain scale 0–10, or verbal pain scale (VPS) 0–3 in 18 trials (S4 Appendix). After conversion to VAS 0–100 mm equivalents values in control groups ranged from 0–80 mm and 0–82 mm at rest and during mobilization, respectively. Mean pain scores in control groups for trials included in the meta-analyses were 38 mm at 6 hours rest, 33 mm at 24 hours rest, 50 mm at 6 hour movement, and 53 mm at 24 hours movement.

Pain scores at rest at 6 hours postoperatively were reported in 84 trials, and at 24 hours postoperatively in 89 trials. Pain during mobilization was reported in 33 trials at 6 hours postoperatively, and in 42 trials at 24 hours postoperatively (S4 Appendix).

### Other outcomes

Ninety trials reported PONV, 24 sedation, 16 dizziness, and 43 pruritus (S4 Appendix).

LOS was reported in 36 trials of which 15 described clearly predefined discharge criteria. No trials before 2001 reported LOS. Of the 36 trials six demonstrated a statistically significant reduction in LOS.

Nineteen trials demonstrated low assay sensitivity for pain score (i.e. pain scores below 30 mm in control groups at 6 or 24h postoperatively). Thirteen trials demonstrated low assay sensitivity for morphine consumption (i.e. no morphine consumption above 15 mg i.v. morphine equivalents 0–24 hours postoperatively in control groups).

### Results related to specific interventions

Seven meta-analyses were carried out. Forest plots for primary and secondary outcomes are presented in Figs 3–5 and S5–S11 Appendices, L’Abbé plots and TSA are presented in S12–S25 Appendices.

**Fig 3:**
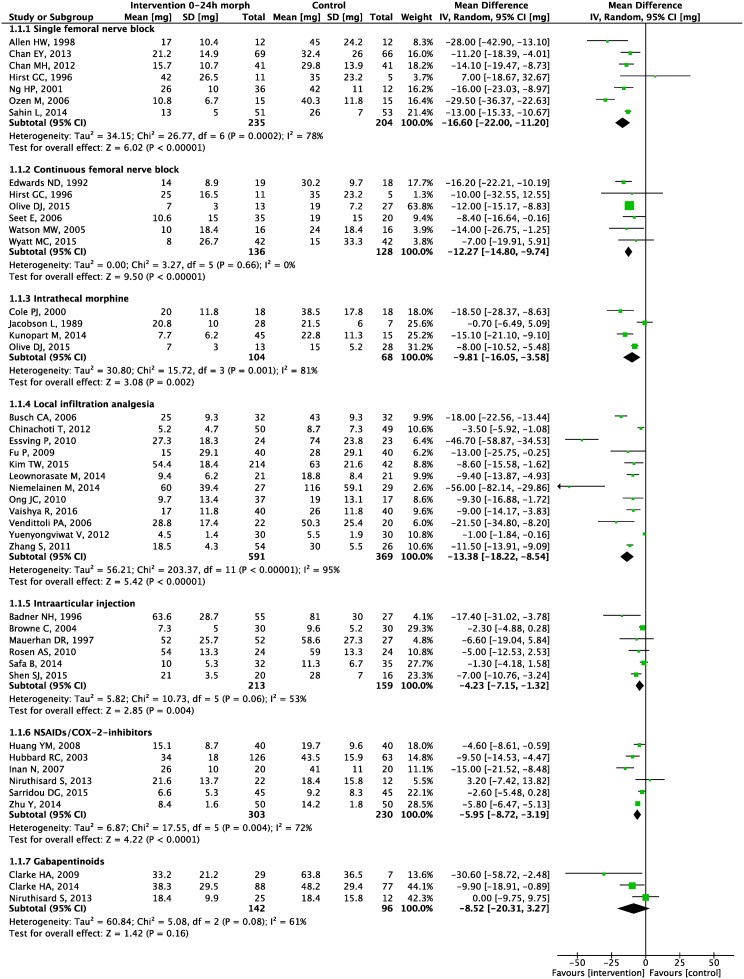
0–24 hour morphine consumption. Forest plot displaying mean difference in 0–24 hour morphine consumption for each meta-analyzed intervention. Green squares with horizontal lines represent mean differences and 95% confidence intervals for each trial. Black tiles represent the mean difference of each intervention.

**Fig 4:**
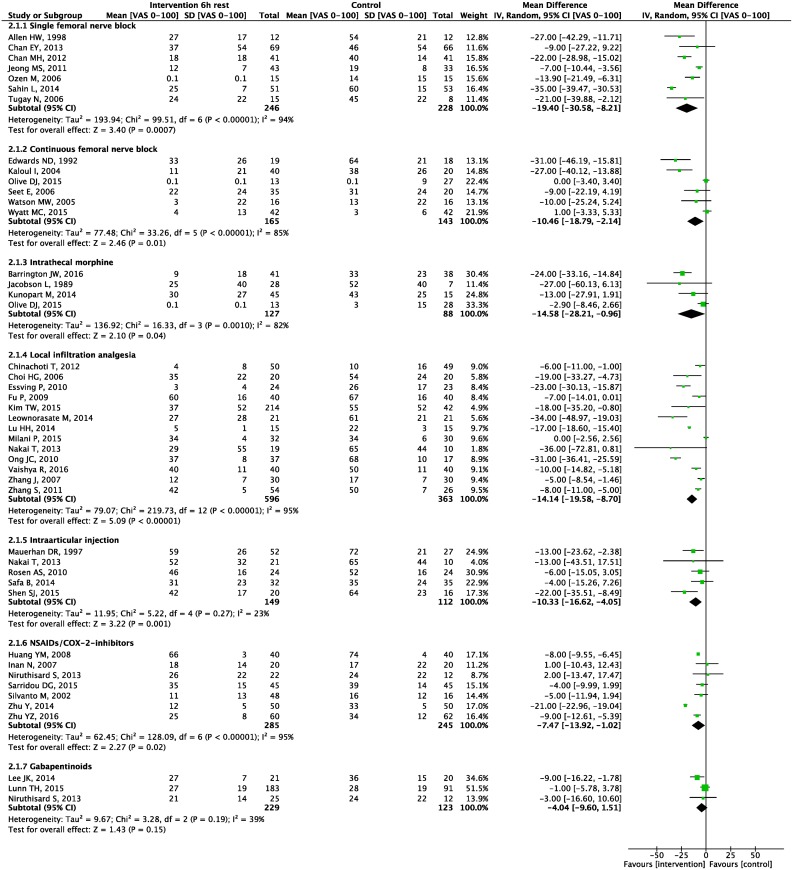
6 hours pain scores. Forest plot displaying mean difference in pain scores 6 hours postoperative at rest for each meta-analyzed intervention. Green squares with horizontal lines represent mean differences and 95% confidence intervals for each trial. Black tiles represent the mean difference of each intervention.

**Fig 5:**
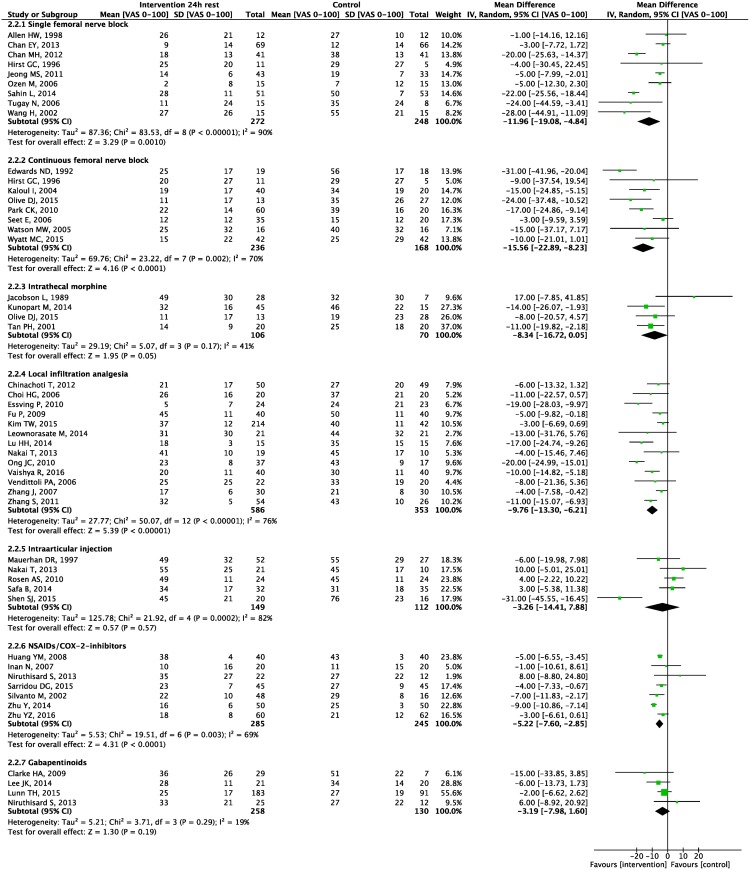
24 hours pain scores. Forest plot displaying mean difference in pain scores 24 hours postoperative at rest for each meta-analyzed intervention. Green squares with horizontal lines represent mean differences and 95% confidence intervals for each trial. Black tiles represent the mean difference of each intervention.

**Fig 6:**
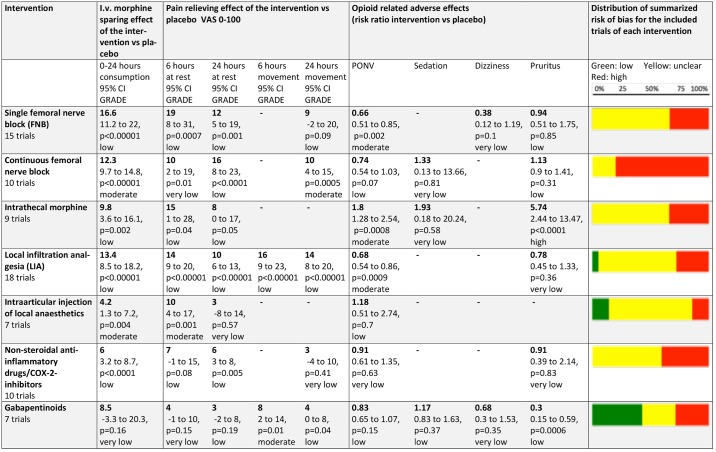
Efficacy, quality of evidence and risk of bias. A summary of each meta-analyzed intervention regarding the effect on each outcome (opioid sparing effect in i.v. morphine equivalents mg, pain scores, and side effects), the GRADE-rated quality of evidence for each outcome and the estimated risk of bias of the included trials. The bold numbers are mean reductions for the relevant outcome, below each bold number is the 95% confidence interval, the p-value and the quality of evidence. Below each intervention the number of trials investigating the specific intervention is depicted. The colored bars to the right depict the distribution of summarized risk of bias for the included trials. Not all trials investigated all relevant outcomes. GRADE: The Grading of Recommandations Assessment, Development and Evaluation.

Fig 6 presents a summary of all the meta-analysed subgroups regarding outcomes, GRADE-rated quality of evidence and the estimated risk of bias of the included trials.

#### Single injection femoral nerve block

Fifteen trials tested single FNB as an intervention [16–30]. Four of these trials tested the intervention in addition to a basic analgesic regimen.

The summarized risk of bias was low in zero, unclear in 10, and high in five trials (Fig 2), and the trial sample size implicated a high risk of bias in seven trials, and a moderate risk in eight trials. L’Abbé plots demonstrated a lower degree of heterogeneity for pain score at rest and moderate degrees for morphine consumption and pain during movement (S12 Appendix).

Meta-analyses demonstrated a statistically significant 0–24 hour postoperative morphine sparing effect of 16.6 mg (95% CI: 11 to 22; p<0.00001) (Fig 3), and a reduction in postoperative pain scores at 6 hours at rest of 19 mm (8 to 31; P = 0.0007), at 24 hours at rest of 12 mm (5 to 19; P = 0.001), and at 24 hours during movement of 9 mm (-2 to 20; P = 0.09) (Figs 4 and 5, S6 Appendix).

In TSA, reductions in both morphine consumption and pain scores at rest at 6 and 24 hours were above the threshold for significance. Morphine consumption and 24 hours pain score at rest reached APIS concluding that single FNB has a positive effect on these outcomes (S13 Appendix).

In meta-analyses, RR for nausea and vomiting was 0.66 (0.51 to 0.85; P = 0.002), for dizziness 0.38 (0.12 to 1.19; P = 0.1) and for pruritus 0.94 (0.51 to 1.75; P = 0.85) (S7, S9 and S10 Appendices).

Urinary retention was registered in four trials [17, 19, 21, 22], deep venous thrombosis (DVT) in two [17, 21], soreness/pain in the back in two [18, 23], hypotension in one [25], numbness around the knee in one [16], and infection around the site of injection in one [26]. No significant differences between active and control groups were reported.

Quality of evidence (GRADE) was moderate for PONV; low for the opioid sparing effect and pain scores and pruritus; and very low for dizziness. Results are summarized in Table 2.

**Table 2:** Summarized outcomes in Grading of Recommendations Assessment, Development and Evaluation (GRADE) for each major intervention.

Table 2 summary of findings:					
**Single femoral nerve block compared to Placebo or no intervention for pain after TKA**					
**Patient or population**: pain after TKA. **Setting**: The immediate postoperative period. **Intervention**: Single femoral nerve block. **Comparison**: Placebo or no intervention					
Outcomes	Anticipated absolute effects* (95% CI)		Relative effect (95% CI)	No of participants (studies)	Quality of the evidence (GRADE)
	**Risk with Placebo or no intervention**	**Risk with Single femoral nerve block**			
Morphine consumption assessed with: 0–24 hour postoperative	The mean morphine consumption was **32.2** mg	The mean morphine consumption in the intervention group was 16.6 mg lower (11.2 lower to 22 lower)	-	439 (7 RCTs)	⨁⨁◯◯ LOW ^a^^,^^b^
Pain score 6 h postoperative at rest assessed with: VAS 0–100	The mean pain score 6 h postoperative at rest was **43** mm	The mean pain score 6 h postoperative at rest in the intervention group was 19 mm lower (8 lower to 31 lower)	-	474 (7 RCTs)	⨁⨁◯◯ LOW ^a^^,^^b^
Pain score 24 h postoperative at rest assessed with: VAS 0–100	The mean pain score 24 h postoperative at rest was **29** mm	The mean pain score 24 h postoperative at rest in the intervention group was 12 mm lower (5 lower to 19 lower)	-	520 (9 RCTs)	⨁⨁◯◯ LOW ^a^^,^^b^
Pain score 24 h postoperative at movement assessed with: VAS 0–100	The mean pain score 24 h postoperative at movement was **52** mm	The mean pain score 24 h postoperative at movement in the intervention group was 9 mm lower (20 lower to 2 higher)	-	391 (6 RCTs)	⨁⨁◯◯ LOW ^a^^,^^b^^,^^c^^,^^d^
Postoperative nausea and vomiting (PONV) assessed with: Number of events	279 per 1,000	**184 per 1,000** (142 to 237)	**RR 0.66** (0.51 to 0.85)	706 (11 RCTs)	⨁⨁⨁◯ MODERATE ^a^
Dizziness assessed with: Number of events	234 per 1,000	**89 per 1,000** (28 to 278)	**RR 0.38** (0.12 to 1.19)	340 (4 RCTs)	⨁◯◯◯ VERY LOW ^a^^,^^b^^,^^d^^,^^e^
Pruritus assessed with: Number of events	113 per 1,000	**106 per 1,000** (57 to 197)	**RR 0.94** (0.51 to 1.75)	464 (6 RCTs)	⨁⨁◯◯ LOW ^a^^,^^d^
Length of stay (LOS)	The mean length of stay was **5.5** days	The mean length of stay in the intervention group was 0.3 days lower (0.9 lower to 0.3 higher)	-	332 (5 RCTs)	⨁◯◯◯ VERY LOW ^a^^,^^b^^,^^d^^,^^e^
*The risk in the intervention group (and its 95% confidence interval) is based on the assumed risk in the comparison group and the **relative effect** of the intervention (and its 95% CI). **CI:** Confidence interval; **MD:** Mean difference; **RR:** Risk ratio					
**GRADE Working Group grades of evidence: High quality:** We are very confident that the true effect lies close to that of the estimate of the effect **Moderate quality:** We are moderately confident in the effect estimate: The true effect is likely to be close to the estimate of the effect, but there is a possibility that it is substantially different **Low quality:** Our confidence in the effect estimate is limited: The true effect may be substantially different from the estimate of the effect **Very low quality:** We have very little confidence in the effect estimate: The true effect is likely to be substantially different from the estimate of effect					

^**a**.^ There were studies of unclear and high summarized risk of bias.

^**b**.^ There was heterogeneity as noted by I^2.

^**c**.^ The intervention did not reach either threshold for significance or a priori estimated information size in trial sequential analysis.

^**d**.^ 95% confidence interval includes 'no effect'.

^**e**.^ There were less than 400 participants in total.

Various local anaesthetics +/- epinephrine were administered in the trials (Table 1). The evidence did not provide information about optimal drug-, and dose-regimens.

### Continuous femoral nerve block

Ten trials tested continuous FNB as an intervention [30–39]. Six of these trials tested the intervention in addition to a basic analgesic regimen.

The summarized risk of bias was low in zero, unclear in two, and high in eight trials (Fig 2), and the trial sample size implicated a high risk of bias in four trials and a moderate risk in six. L’Abbé plots demonstrated homogeneity for morphine consumption and pain scores (S14 Appendix).

Meta-analyses demonstrated a statistically significant 0–24 hour postoperative morphine sparing effect of 12.3 mg (95% CI: 9.7 to 14.8; P<0.00001) (Fig 3), and a reduction in pain scores at rest at 6 hours postoperatively of 10 mm (2 to 19; P = 0.01), at 24 hours at rest of 16 mm (8 to 23; P<0.00001) and at 24 hours during movement of 10 mm (4 to 15; P = 0.0005) (Figs 4 and 5, S6 Appendix).

In TSA, reductions in both morphine consumption and pain scores at rest at 6 hours and 24 hours, and pain scores during movement at 24 hours, were above the threshold for significance and reached APIS, concluding that continuous femoral nerve block has a positive effect on these outcomes (S15 Appendix).

In meta-analyses, RR for nausea and vomiting was 0.74 (0.54 to 1.03, P = 0.07), for sedation 1.33 (0.13 to 13.66; P = 0.81) and for pruritus 1.13 (0.9 to 1.41; P = 0.31) (S7, S8 and S10 Appendices). One study demonstrated a significant increase in obturator motor blockade at 6 hours postoperatively [33]. Urinary retention was registered in one trial [35], cardiac events in one [38] and hypotension in two [35, 37]. No significant differences between active and control groups were reported.

Quality of evidence (GRADE) was moderate for reduction in morphine consumption and pain score at 24 hours during movement; low for 24 hours pain score at rest, PONV and pruritus; and very low for 6 hours pain score at rest and sedation. Results are summarized in Table 3.

**Table 3:** Summarized outcomes in Grading of Recommendations Assessment, Development and Evaluation (GRADE) for each major intervention.

Table 3 summary of findings:					
**Continuous femoral nerve block compared to Placebo or no intervention for pain after TKA**					
**Patient or population**: pain after TKA. **Setting**: The immediate postoperative setting. **Intervention**: Continuous femoral nerve block. **Comparison**: Placebo or no intervention					
Outcomes	Anticipated absolute effects* (95% CI)		Relative effect (95% CI)	No of participants (studies)	Quality of the evidence (GRADE)
	**Risk with Placebo or no intervention**	**Risk with Continuous femoral nerve block**			
Morphine consumption assessed with: 0–24 hour postoperative	The mean morphine consumption was **20.5** mg	The mean morphine consumption in the intervention group was 12.3 mg lower (9.7 lower to 14.8 lower)	-	264 (6 RCTs)	⨁⨁⨁◯ MODERATE ^a^
Pain score 6 h postoperative at rest assessed with: VAS 0–100	The mean pain score 6 h postoperative at rest was **20** mm	The mean pain score 6 h postoperative at rest in the intervention group was 10 mm lower (2 lower to 19 lower)	-	308 (6 RCTs)	⨁◯◯◯ VERY LOW ^a^^,^^b^^,^^c^^,^^d^
Pain score 24 h postoperative at rest assessed with: VAS 0–100	The mean pain score 24 h postoperative at rest was **33** mm	The mean pain score 24 h postoperative at rest in the intervention group was 16 mm lower (8 lower to 23 lower)	-	404 (8 RCTs)	⨁⨁◯◯ LOW ^a^^,^^b^^,^^e^
Pain score 24 h postoperative at movement assessed with: VAS 0–100	The mean pain score 24 h postoperative at movement was **64** mm	The mean pain score 24 h postoperative at movement in the intervention group was 10 mm lower (4 lower to 15 lower)	-	183 (4 RCTs)	⨁⨁⨁◯ MODERATE ^a^
Postoperative nausea and vomiting (PONV) assessed with: Number of events	580 per 1,000	**429 per 1,000** (313 to 597)	**RR 0.74** (0.54 to 1.03)	220 (5 RCTs)	⨁⨁◯◯ LOW ^a^^,^^c^^,^^f^
Sedation assessed with: Number of events	286 per 1,000	**380 per 1,000** (37 to 1,000)	**RR 1.33** (0.13 to 13.66)	167 (3 RCTs)	⨁◯◯◯ VERY LOW ^a^^,^^b^^,^^c^^,^^f^
Pruritus assessed with: Number of events	507 per 1,000	**573 per 1,000** (457 to 716)	**RR 1.13** (0.90 to 1.41)	175 (3 RCTs)	⨁⨁◯◯ LOW ^a^^,^^c^^,^^f^
Length of stay (LOS)	The mean length of stay was **6.4** days	The mean length of stay in the intervention group was 0.3 days lower (0.8 lower to 0.2 higher)	-	171 (3 RCTs)	⨁⨁◯◯ LOW ^a^^,^^c^^,^^f^
*The risk in the intervention group (and its 95% confidence interval) is based on the assumed risk in the comparison group and the **relative effect** of the intervention (and its 95% CI). **CI:** Confidence interval; **MD:** Mean difference; **RR:** Risk ratio					
**GRADE Working Group grades of evidence: High quality:** We are very confident that the true effect lies close to that of the estimate of the effect. **Moderate quality:** We are moderately confident in the effect estimate: The true effect is likely to be close to the estimate of the effect, but there is a possibility that it is substantially different. **Low quality:** Our confidence in the effect estimate is limited: The true effect may be substantially different from the estimate of the effect. **Very low quality:** We have very little confidence in the effect estimate: The true effect is likely to be substantially different from the estimate of effect.					

^**a**.^ There were studies of unclear and high summarized risk of bias.

^**b**.^ There was heterogeneity as noted by I^2.

^**c**.^ There were less than 400 participants in total.

^**d**.^ The intervention did not reach either threshold for significance or a priori estimated information size in trial sequential analysis.

^**e**.^ Low assay sensitivity in Seet et al explains the heterogeneity.

^**f**.^ 95% confidence interval includes 'no effect'.

Various local anaesthetics +/- epinephrine were administered in all trials (Table 1). The evidence did not allow designation of optimal drug-, and dose-regimens.

### Intrathecal morphine adjunct to local anaesthetics

Nine trials tested intrathecal morphine as an intervention [39–47]. Four of these trials tested the intervention in addition to a basic analgesic regimen.

The summarized risk of bias was low in zero, unclear in six, and high in three trials (Fig 2), and the trial sample size implicated a high risk of bias in two trials and a moderate risk in seven. L’Abbé plots demonstrated moderate degrees of heterogeneity for morphine consumption and pain scores (S16 Appendix).

Meta-analyses demonstrated a statistically significant 0–24 hour postoperative morphine sparing effect of 9.8 mg (95% CI: 3.6 to 16.1, P = 0.002) (Fig 3), a reduction in pain scores at rest at 6 hours postoperatively of 15 mm (1 to 28, P = 0.04), and at 24 hours at rest of 8 mm (0 to 17; P = 0.05) (Figs 4 and 5).

In TSA, morphine consumption reached the threshold for significance but not APIS. Pain score at 24 hours rest reached the boundary for futility and APIS concluding that there is no reason for further investigation of this outcome (S17 Appendix).

In meta-analyses, RR for nausea and vomiting was 1.8 (1.28 to 2.54; P = 0.0008), for sedation 1.93 (0.18 to 20.24; P = 0.58) and for pruritus 5.74 (2.44 to 13.47; P<0.0001) (S7, S8 and S10 Appendices). Hypoxemia was registered in one trial [41], respiratory depression in two [41, 47], urinary retention in three [42, 43, 45] and anxiety in one [47]. No significant differences between active and control groups were reported.

Quality of evidence (GRADE) was high for the increase in pruritus; moderate for increase in PONV; low for opioid sparing effect and reduction in pain score at 6 and 24 hours at rest; and very low for the increase in sedation. Results are summarized in Table 4.

**Table 4:** Summarized outcomes in Grading of Recommendations Assessment, Development and Evaluation (GRADE) for each major intervention.

Table 4 summary of findings:					
**Intrathecal morphine compared to Placebo or no intervention for pain after TKA**					
**Patient or population**: pain after TKA. **Setting**: The immediate postoperative setting. **Intervention**: Intrathecal morphine. **Comparison**: Placebo or no intervention.					
Outcomes	Anticipated absolute effects* (95% CI)		Relative effect (95% CI)	No of participants (studies)	Quality of the evidence (GRADE)
	**Risk with Placebo or no intervention**	**Risk with Intrathecal morphine**			
Morphine consumption assessed with: 0–24 hour postoperative	The mean morphine consumption was **23.6** mg	The mean morphine consumption in the intervention group was 9.8 mg lower (3.6 lower to 16.1 lower)	-	172 (4 RCTs)	⨁⨁◯◯ LOW ^a^^,^^b^
Pain score 6 h postoperative at rest assessed with: VAS 0–100	The mean pain score 6 h postoperative at rest was **27** mm	The mean pain score 6 h postoperative at rest in the intervention group was 15 mm lower (28 lower to 1 lower)	-	215 (4 RCTs)	⨁⨁◯◯ LOW ^a^^,^^b^^,^^c^^,^^d^^,^^e^
Pain score 24 h postoperative at rest assessed with: VAS 0–100	The mean pain score 24 h postoperative at rest was **28** mm	The mean pain score 24 h postoperative at rest in the intervention group was 8 mm lower (17 lower to 0)	-	176 (4 RCTs)	⨁⨁◯◯ LOW ^a^^,^^b^^,^^e^
Postoperative nausea and vomiting (PONV) assessed with: Number of events	165 per 1,000	**297 per 1,000** (209 to 419)	**RR 1.80** (1.27 to 2.54)	496 (9 RCTs)	⨁⨁⨁◯ MODERATE ^a^
Sedation assessed with: Number of events	18 per 1,000	**35 per 1,000** (3 to 368)	**RR 1.93** (0.18 to 20.24)	214 (3 RCTs)	⨁◯◯◯ VERY LOW ^a^^,^^b^^,^^e^^,^^f^
Pruritus assessed with: Number of events	67 per 1,000	**383 per 1,000** (163 to 898)	**RR 5.74** (2.44 to 13.47)	494 (9 RCTs)	⨁⨁⨁⨁ HIGH ^a^^,^^b^^,^ ^h^
*The risk in the intervention group (and its 95% confidence interval) is based on the assumed risk in the comparison group and the **relative effect** of the intervention (and its 95% CI). **CI:** Confidence interval; **MD:** Mean difference; **RR:** Risk ratio					
**GRADE Working Group grades of evidence**: **High quality:** We are very confident that the true effect lies close to that of the estimate of the effect. **Moderate quality:** We are moderately confident in the effect estimate: The true effect is likely to be close to the estimate of the effect, but there is a possibility that it is substantially different. **Low quality:** Our confidence in the effect estimate is limited: The true effect may be substantially different from the estimate of the effect. **Very low quality:** We have very little confidence in the effect estimate: The true effect is likely to be substantially different from the estimate of effect					

^**a**.^ There were studies of unclear and high summarized risk of bias.

^**b**.^ There was heterogeneity as noted by I^2.

^**c**.^ Low assay sensitivity in Olive et al explains the heterogeneity.

^**d**.^ The intervention did not reach either threshold for significance or a priori estimated information size in trial sequential analysis.

^**e**.^ 95% confidence interval includes 'no effect'.

^**f**.^ There were less than 400 participants in total.

^**h**.^ RR above five.

Diamorphine was administered in one trial and morphine in the others. Due to heterogeneity amongst trials there were no dose-response relationship and the evidence did not provide information regarding optimal dosages.

### Local Infiltration Analgesia (LIA)

Eighteen trials tested LIA as an intervention [48–65]. Thirteen of these trials tested the intervention in addition to a basic analgesic regimen.

The summarized risk of bias was low in one, unclear in 12, and high in five trials (Fig 2), and the trial sample size implicated a high risk of bias in six trials, a moderate risk in 11 and a low risk in one. L’Abbé plots demonstrated low degrees of heterogeneity for morphine consumption and pain scores at rest. Moderate degrees were present for pain scores during movement (S18 Appendix).

Meta-analyses demonstrated a statistically significant 0–24 hour postoperative morphine sparing effect of 13.4 mg (95% CI: 8.5 to 18.2; P<0.00001) (Fig 3), and a reduction in pain scores at rest at 6 hours postoperatively of 14 mm (9 to 20; P<0.00001), at 24 hours rest of 10 mm (6 to 13; P<0.00001), at 6 hours during movement of 16 mm (9 to 23; P<0.00001) and at 24 hours during movement of 14 mm (8 to 20; P<0.00001) (Figs 4 and 5, S5 and S6 Appendices).

In TSA, threshold for significance and APIS were reached for all outcomes, concluding that LIA has a positive effect on these outcomes (S19 Appendix).

In meta-analyses RR for nausea and vomiting was 0.68 (0.54 to 0.86; P = 0.0009) and for pruritus 0.78 (0.45 to 1.33; P = 0.36) (S7 and S10 Appendices). One study demonstrated a significant reduction in blood loss [55] and one demonstrated a significant increase in skin blisters due to cannula [59]. Hypotension was registered in one trial [50], respiratory distress/depression in two [50, 64], headache in one [50], positive cultures from the catheter tips in one [51], rash in one [52], urinary retention in seven [52, 54, 59–62, 64], DVT in four [52, 61, 63, 64], incision complications in three [52, 54, 55], cardiac or CNS events in two [55, 63], slight numbness in one [56] and constipation in one [62]. No significant differences between active and control groups were reported.

Quality of evidence (GRADE) was moderate for PONV; low for the opioid sparing effect and reduction in pain scores, and very low for pruritus. Results are summarized in Table 5.

**Table 5:** Summarized outcomes in Grading of Recommendations Assessment, Development and Evaluation (GRADE) for each major intervention.

Table 5 summary of findings:					
**Local infiltration analgesia compared to Placebo or no intervention for pain after TKA**					
**Patient or population**: pain after TKA. **Setting**: The immediate postoperative setting. **Intervention**: Local infiltration analgesia. **Comparison**: Placebo or no intervention					
Outcomes	Anticipated absolute effects* (95% CI)		Relative effect (95% CI)	No of participants (studies)	Quality of the evidence (GRADE)
	**Risk with Placebo or no intervention**	**Risk with Local infiltration analgesia**			
Morphine consumption assessed with: 0–24 hour postoperative	The mean morphine consumption was **38.9** mg	The mean morphine consumption in the intervention group was 13.4 mg lower (8.5 lower to 18.2 lower)	-	960 (12 RCTs)	⨁⨁◯◯ LOW ^a^^,^^b^
Pain score 6 h postoperative at rest assessed with: VAS 0–100	The mean pain score 6 h postoperative at rest was **42** mm	The mean pain score 6 h postoperative at rest in the intervention group was 14 mm lower (9 lower to 20 lower)	-	959 (13 RCTs)	⨁⨁◯◯ LOW ^a^^,^^b^
Pain score 24 h postoperative at rest assessed with: VAS 0–100	The mean pain score 24 h postoperative at rest was **36** mm	The mean pain score 24 h postoperative at rest in the intervention group was 10 mm lower (6 lower to 13 lower)	-	939 (13 RCTs)	⨁⨁◯◯ LOW ^a^^,^^b^
Pain score 6 h postoperative at movement assessed with: VAS 0–100	The mean pain score 6 h postoperative at movement was **50** mm	The mean pain score 6 h postoperative at movement in the intervention group was 16 mm lower (9 lower to 23 lower)	-	361 (6 RCTs)	⨁⨁◯◯ LOW ^a^^,^^b^
Pain score 24 h postoperative at movement assessed with: VAS 0–100	The mean pain score 24 h postoperative at movement was **59** mm	The mean pain score 24 h postoperative at movement in the intervention group was 14 mm lower (8 lower to 20 lower)	-	377 (6 RCTs)	⨁⨁◯◯ LOW ^a^^,^^b^
Postoperative nausea and vomiting (PONV) assessed with: Number of events	273 per 1,000	**186 per 1,000** (147 to 235)	**RR 0.68** (0.54 to 0.86)	795 (10 RCTs)	⨁⨁⨁◯ MODERATE ^a^
Pruritus assessed with: Number of events	314 per 1,000	**245 per 1,000** (141 to 417)	**RR 0.78** (0.45 to 1.33)	368 (6 RCTs)	⨁◯◯◯ VERY LOW ^a^^,^^b^^,^^c^^,^^d^
Length of stay (LOS)	The mean length of stay was **5.6** days	The mean length of stay in the intervention group was 1 days lower (1.9 lower to 0.2 lower)	-	449 (8 RCTs)	⨁⨁◯◯ LOW ^a^^,^^b^
*The risk in the intervention group (and its 95% confidence interval) is based on the assumed risk in the comparison group and the **relative effect** of the intervention (and its 95% CI). **CI:** Confidence interval; **MD:** Mean difference; **RR:** Risk ratio					
**GRADE Working Group grades of evidence**: **High quality:** We are very confident that the true effect lies close to that of the estimate of the effect. **Moderate quality:** We are moderately confident in the effect estimate: The true effect is likely to be close to the estimate of the effect, but there is a possibility that it is substantially different. **Low quality:** Our confidence in the effect estimate is limited: The true effect may be substantially different from the estimate of the effect. **Very low quality:** We have very little confidence in the effect estimate: The true effect is likely to be substantially different from the estimate of effect					

^**a**.^ There were studies of unclear and high summarized risk of bias.

^**b**.^ There was heterogeneity as noted by I^2.

^**c**.^ There were less than 400 participants in total.

^**d**.^ 95% confidence interval includes 'no effect'.

Trials were too heterogeneous (administration of different combinations of local anaesthetics, morphine, NSAIDs, steroids and epinephrine, Table 1) to provide information about optimal drug-, and dose-regimens.

### Intraarticular injection of local anaesthetics

Seven trials tested intraarticular injection of local anaesthetics as an intervention [65–71]. Two of these trials tested the intervention in addition to a basic analgesic regimen.

The summarized risk of bias was low in one, unclear in five, and high in one trial (Fig 2), and the trial sample size implicated a high risk of bias in two trials and a moderate risk in five. L’Abbé plots demonstrated homogeneity for morphine consumption and pain scores at rest at 6 hours and higher degrees of heterogeneity at 24 hours (S20 Appendix).

Meta-analyses demonstrated a statistically significant 0–24 hour postoperative morphine sparing effect of 4.2 mg (95% CI: 1.3 to 7.2; P = 0.004) (Fig 3), and a reduction in pain scores at rest at 6 hours postoperatively of 10 mm (4 to 17; 0.001) and at 24 hours at rest of 3 mm (-8 to 14; P = 0.57) (Figs 4 and 5).

In TSA, reductions in both morphine consumption and pain scores at rest at 6 hours were above the threshold for significance and reached APIS concluding that intraarticular injection has a positive effect on these outcomes (S21 Appendix).

In meta-analyses RR for nausea and vomiting was 1.18 (0.51 to 2.74; P = 0.70) (S7 Appendix). Respiratory depression was registered in two trials [67, 69], sinus tachycardia in one [67], DVT in one [69], wound healing complications in one [69]. No significant differences between active and control groups were reported.

Quality of evidence (GRADE) was moderate for opioid sparing effect and 6 hours pain score; low for increase in PONV; and very low for 24 hours pain score. Results are summarized in Table 6.

**Table 6:** Summarized outcomes in Grading of Recommendations Assessment, Development and Evaluation (GRADE) for each major intervention.

Table 6 summary of findings:					
**Intraarticular injection compared to Placebo or no intervention for pain after TKA**					
**Patient or population**: pain after TKA. **Setting**: The immediate postoperative setting. **Intervention**: Intraarticular injection. **Comparison**: Placebo or no intervention					
Outcomes	Anticipated absolute effects* (95% CI)		Relative effect (95% CI)	No of participants (studies)	Quality of the evidence (GRADE)
	**Risk with Placebo or no intervention**	**Risk with Intraarticular injection**			
Morphine consumption assessed with: 0–24 hour postoperative	The mean morphine consumption was **39.7** mg	The mean morphine consumption in the intervention group was 4.2 mg lower (1.3 lower to 7.2 lower)	-	372 (6 RCTs)	⨁⨁⨁◯ MODERATE ^a^^,^^b^
Pain score 6 h postoperative at rest assessed with: VAS 0–100	The mean pain score 6 h postoperative at rest was **54** mm	The mean pain score 6 h postoperative at rest in the intervention group was 10 mm lower (4 lower to 17 lower)	-	261 (5 RCTs)	⨁⨁⨁◯ MODERATE ^c^
Pain score 24 h postoperative at rest assessed with: VAS 0–100	The mean pain score 24 h postoperative at rest was **47** mm	The mean pain score 24 h postoperative at rest in the intervention group was 3 mm lower (14 lower to 8 higher)	-	261 (5 RCTs)	⨁◯◯◯ VERY LOW ^a^^,^^c^^,^^d^^,^^e^
Postoperative nausea and vomiting (PONV) assessed with: Number of events	219 per 1,000	**258 per 1,000** (112 to 599)	**RR 1.18** (0.51 to 2.74)	139 (3 RCTs)	⨁⨁◯◯ LOW ^c^^,^^e^^,^^f^
*The risk in the intervention group (and its 95% confidence interval) is based on the assumed risk in the comparison group and the **relative effect** of the intervention (and its 95% CI). **CI:** Confidence interval; **MD:** Mean difference; **RR:** Risk ratio					
**GRADE Working Group grades of evidence**: **High quality:** We are very confident that the true effect lies close to that of the estimate of the effect. **Moderate quality:** We are moderately confident in the effect estimate: The true effect is likely to be close to the estimate of the effect, but there is a possibility that it is substantially different. **Low quality:** Our confidence in the effect estimate is limited: The true effect may be substantially different from the estimate of the effect. **Very low quality:** We have very little confidence in the effect estimate: The true effect is likely to be substantially different from the estimate of effect					

^**a**.^ There was heterogeneity as noted by I^2.

^**b**.^ Low assay sensitivity for Browne et al and Safa et al explains heterogeneity however confidence intervals for separate trials are not convincing.

^**c**.^ There were studies of unclear and high summarized risk of bias.

^**d**.^ The intervention did not reach either threshold for significance or a priori estimated information size in trial sequential analysis.

^**e**.^ 95% confidence interval includes 'no effect'.

^**f**.^ There were less than 400 participants in total.

Trials were too heterogeneous (administration of different combinations of local anaesthetics, morphine, steroids and epinephrine, Table 1) to provide information about optimal drug-, and dose-regimens.

### NSAIDs/COX-2-inhibitors

Ten trials tested NSAIDs/COX-2-inhibitors as an intervention [72–81]. Two of these trials tested the intervention in addition to a basic analgesic regimen.

The summarized risk of bias was low in zero, unclear in six, and high in four trials (Fig 2) and the trial sample size implicated a high risk of bias in two trials, a moderate risk in seven and a very low risk in one. L’Abbé plots demonstrated low degrees of heterogeneity for morphine consumption and moderate degrees for pain scores (S22 Appendix).

Meta-analyses demonstrated a statistically significant 0–24 hour postoperative morphine sparing effect of 6 mg (95% CI: 3.2 to 8.7; P<0.0001) (Fig 3), and a reduction in pain scores at rest at 6 hours postoperatively of 7 mm (1 to 14; P = 0.02), at 24 hours at rest of 5 mm (3 to 8; P<0.0001), and at 24 hours during movement of 3 mm (-4 to 10; P = 0.41) (Figs 4 and 5, S6 Appendix).

In TSA, threshold for significance and APIS were reached for morphine consumption and pain at 6 and 24 hours rest concluding that NSAIDs and COX-2-inhibitors have a positive effect on these outcomes. The reduction in pain scores during movement at 24 hours reached the threshold for futility and APIS (S23 Appendix).

In meta-analyses RR for nausea and vomiting was 0.91 (0.61 to 1.35 P = 0.63) and for pruritus 0.91 (0.39 to 2.14; P = 0.83) (S7 and S10 Appendices).

Bleeding was registered in one trial [73], hypo/hypertension in one [74], anemia in two [74, 76], urinary retention in three [74, 76, 78], dry mouth in one [75], gastric pain in one [78] and constipation, hyperhidrosis, pyrexia, headache and confusion in one [76]. No significant differences between active and control groups were reported.

Quality of evidence (GRADE) was low for the opioid sparing effect and reduction in pain score at 6 and 24 hours at rest, and very low for remaining outcomes. Results are summarized in Table 7.

**Table 7:** Summarized outcomes in Grading of Recommendations Assessment, Development and Evaluation (GRADE) for each major intervention.

Table 7 summary of findings:					
**NSAIDs or COX-2-inhibitors compared to Placebo or no intervention for pain after TKA**					
**Patient or population**: pain after TKA. **Setting**: The immediate postoperative setting. **Intervention**: NSAIDs or COX-2-inhibitors. **Comparison**: Placebo or no intervention					
Outcomes	Anticipated absolute effects* (95% CI)		Relative effect (95% CI)	No of participants (studies)	Quality of the evidence (GRADE)
	**Risk with Placebo or no intervention**	**Risk with NSAIDs or COX-2-inhibitors**			
Morphine consumption assessed with: 0–24 hour postoperative	The mean morphine consumption was **24.8** mg	The mean morphine consumption in the intervention group was 6 mg lower (3.2 lower to 8.7 lower)	-	533 (6 RCTs)	⨁⨁◯◯ LOW ^a^^,^^b^
Pain score 6 h postoperative at rest assessed with: VAS 0–100	The mean pain score 6 h postoperative at rest was **23** mm	The mean pain score 6 h postoperative at rest in the intervention group was 7 mm lower (15 lower to 1 higher)	-	408 (6 RCTs)	⨁⨁◯◯ LOW ^a^^,^^b^^,^^c^
Pain score 24 h postoperative at rest assessed with: VAS 0–100	The mean pain score 24 h postoperative at rest was **28** mm	The mean pain score 24 h postoperative at rest in the intervention group was 6 mm lower (3 lower to 8 lower)	-	408 (6 RCTs)	⨁⨁◯◯ LOW ^a^^,^^b^
Pain score 24 h postoperative at movement assessed with: VAS 0–100	The mean pain score 24 h postoperative at movement was **46** mm	The mean pain score 24 h postoperative at movement in the intervention group was 3 mm lower (10 lower to 4 higher)	-	214 (3 RCTs)	⨁◯◯◯ VERY LOW ^a^^,^^b^^,^^c^
Postoperative nausea and vomiting (PONV) assessed with: Number of events	234 per 1,000	**213 per 1,000** (143 to 316)	**RR 0.91** (0.61 to 1.35)	1218 (7 RCTs)	⨁◯◯◯ VERY LOW ^a^^,^^b^^,^^c^
Pruritus assessed with: Number of events	115 per 1,000	**104 per 1,000** (45 to 245)	**RR 0.91** (0.39 to 2.14)	849 (3 RCTs)	⨁◯◯◯ VERY LOW ^a^^,^^b^^,^^c^
*The risk in the intervention group (and its 95% confidence interval) is based on the assumed risk in the comparison group and the **relative effect** of the intervention (and its 95% CI). **CI:** Confidence interval; **MD:** Mean difference; **RR:** Risk ratio					
**GRADE Working Group grades of evidence**:**High quality:** We are very confident that the true effect lies close to that of the estimate of the effect. **Moderate quality:** We are moderately confident in the effect estimate: The true effect is likely to be close to the estimate of the effect, but there is a possibility that it is substantially different. **Low quality:** Our confidence in the effect estimate is limited: The true effect may be substantially different from the estimate of the effect. **Very low quality:** We have very little confidence in the effect estimate: The true effect is likely to be substantially different from the estimate of effect					

^**a**.^ There were studies of unclear and high summarized risk of bias.

^**b**.^ There was heterogeneity as noted by I^2.

^**c**.^ 95% confidence interval includes 'no effect'.

Trials were too heterogeneous (time of administration, specific drugs, oral/i.v. administration) to provide information about optimal drug-, and dose-regimens.

### Gabapentinoids

Seven trials tested gabapentinoids as an intervention [81–87]. Six of these trials tested the intervention in addition to a basic analgesic regimen.

The summarized risk of bias was low in three, unclear in two, and high in two trials (Fig 2) and the trial sample size implicated a high risk of bias in two trials, a moderate risk in four and a low risk in one. L’Abbé plots demonstrated moderate degrees heterogeneity for morphine consumption and pain scores (S24 Appendix).

Meta-analyses demonstrated non-significant reductions for 0–24 hour postoperative morphine sparing effect of 8.5 mg (95% CI: -3.3 to 20.3; P = 0.16) (Fig 3), and pain scores at rest at 6 hours postoperatively of 4 mm (-1 to 10; P = 0.15) and at 24 hours at rest of 3 mm (-2 to 8; P = 0.19). Significant reductions in pain scores at 6 and 24 hours during movement of 8 mm (2 to 14; P = 0.01) and 4 mm (0 to 8; P = 0.04), respectively, were demonstrated (Figs 4 and 5, S5 and S6 Appendices).

In TSA, threshold for significance and APIS were reached for pain at 6 and 24 hours during movement concluding that gabapentinoids have a positive effect on these outcomes. Threshold for futility and APIS were reached for pain at rest at 6 and 24 hours concluding that further testing of these outcomes is futile (S25 Appendix).

In meta-analyses RR for nausea and vomiting was 0.83 (0.65 to 1.07, P = 0.15), for sedation 1.17 (0.83 to 1.63, P = 0.37), for dizziness 0.68 (0.3 to 1.53, P = 0.35) and for pruritus 0.3 (0.15 to 0.59; P = 0.0006) (S7–S10 Appendices).

One study reported an accumulation of undesirable reactions due to the study drug in the intervention groups; lapse of memory function, impaired balance, hypotension, diplopia, sedation, dizziness and fatigue [85].

Quality of evidence (GRADE) was moderate for pain score at 6 hours at movement; low for pain scores at 24 hours at rest and during movement, PONV, sedation and pruritus; and very low for opioid sparing effect, reduction in pain score at 6 hours at rest and dizziness. Results are summarized in Table 8.

**Table 8:** Summarized outcomes in Grading of Recommendations Assessment, Development and Evaluation (GRADE) for each major intervention.

Table 8 summary of findings:					
**Gabapentinoids compared to Placebo or no intervention for pain after TKA**					
**Patient or population**: pain after TKA. **Setting:** The immediate postoperative setting. **Intervention**: Gabapentinoids. **Comparison**: Placebo or no intervention					
Outcomes	Anticipated absolute effects* (95% CI)		Relative effect (95% CI)	No of participants (studies)	Quality of the evidence (GRADE)
	**Risk with Placebo or no intervention**	**Risk with Gabapentinoids**			
Morphine consumption assessed with: 0–24 hour postoperative	The mean morphine consumption was **45.6** mg	The mean morphine consumption in the intervention group was 8.5 mg lower (20.3 lower to 3.3 higher)	-	238(3 RCTs)	⨁◯◯◯ VERY LOW ^a^^,^^b^^,^^c^^,^^d^
Pain score 6 h postoperative at rest assessed with: VAS 0–100	The mean pain score 6 h postoperative at rest was **29** mm	The mean pain score 6 h postoperative at rest in the intervention group was 4 mm lower (10 lower to 1 higher)	-	352 (3 RCTs)	⨁◯◯◯ VERY LOW ^a^^,^^b^^,^^d^
Pain score 24 h postoperative at rest assessed with: VAS 0–100	The mean pain score 24 h postoperative at rest was **29** mm	The mean pain score 24 h postoperative at rest in the intervention group was 3 mm lower (8 lower to 2 higher)	-	388 (4 RCTs)	⨁⨁◯◯ LOW ^a^^,^^d^
Pain score 6 h postoperative at movement assessed with: VAS 0–100	The mean pain score 6 h postoperative at movement was **45** mm	The mean pain score 6 h postoperative at movement in the intervention group was 8 mm lower (2 lower to 14 lower)	-	352 (3 RCTs)	⨁⨁⨁◯ MODERATE ^a^
Pain score 24 h postoperative at movement assessed with: VAS 0–100	The mean pain score 24 h postoperative at movement was **51** mm	The mean pain score 24 h postoperative at movement in the intervention group was 4 mm lower (0.1 lower to 8 lower)	-	517 (4 RCTs)	⨁⨁◯◯ LOW ^a^^,^^d^
Postoperative nausea and vomiting (PONV) assessed with: Number of events	374 per 1,000	**311 per 1,000** (243 to 401)	**RR 0.83** (0.65 to 1.07)	497 (6 RCTs)	⨁⨁◯◯ LOW ^a^^,^^d^
Sedation assessed with: Number of events	267 per 1,000	**312 per 1,000** (221 to 435)	**RR 1.17** (0.83 to 1.63)	305 (4 RCTs)	⨁⨁◯◯ LOW ^a^^,^^d^^,^^e^
Dizziness assessed with: Number of events	417 per 1,000	**283 per 1,000** (125 to 638)	**RR 0.68** (0.30 to 1.53)	179 (3 RCTs)	⨁◯◯◯ VERY LOW ^a^^,^^b^^,^^d^^,^^e^
Pruritus assessed with: Number of events	282 per 1,000	**84 per 1,000** (42 to 166)	**RR 0.30** (0.15 to 0.59)	382 (5 RCTs)	⨁⨁◯◯ LOW ^a^^,^^b^^,^^e^^,^^f^
Length of stay (LOS)	The mean length of stay was **2.9** days	The mean length of stay in the intervention group was 0.1 days higher (0.7 lower to 0.9 higher)	-	490 (3 RCTs)	⨁⨁◯◯ LOW ^b^^,^^d^
*The risk in the intervention group (and its 95% confidence interval) is based on the assumed risk in the comparison group and the **relative effect** of the intervention (and its 95% CI). **CI:** Confidence interval; **MD:** Mean difference; **RR:** Risk ratio					
**GRADE Working Group grades of evidence**: **High quality:** We are very confident that the true effect lies close to that of the estimate of the effect. **Moderate quality:** We are moderately confident in the effect estimate: The true effect is likely to be close to the estimate of the effect, but there is a possibility that it is substantially different. **Low quality:** Our confidence in the effect estimate is limited: The true effect may be substantially different from the estimate of the effect. **Very low quality:** We have very little confidence in the effect estimate: The true effect is likely to be substantially different from the estimate of effect					

^**a**^. There were studies of unclear and high summarized risk of bias.

^**b**^. There was heterogeneity as noted by I^2.

^**c**^. The intervention did not reach either threshold for significance or a priori estimated information size in trial sequential analysis.

^**d**^. 95% confidence interval includes 'no effect'.

^**e**^. There were less than 400 participants in total.

^**f**^. RR below 0.5.

Trials were too heterogeneous (time of administration and specific drugs) to provide information about optimal drug-, and dose-regimens.

### Qualitative analyses

Forty-one trials investigated other interventions: Adductor canal block [88–91]; clonidine, tramadol, fentanyl, magnesium or buprenorphine added to FNB [92–95]; single and continuous sciatic plexus nerve block [96–100]; obturator nerve block [101, 102]; continuous lumbar plexus block [103]; morphine, ketamine or epinephrine added to intraarticular injections of local anaesthetics [104–107]; continuous intraarticular injection of local anaesthetics [108–110]; ketorolac added to periarticular injection of local anaesthetic [111]; steroids added to LIA [112–114]; epidural analgesia with ropivacaine and morphine [115]; magnesium, midazolam or morphine added to epidural bupivacaine/ropivacaine [116, 117]; fentanyl patch [118, 119]; i.v. tramadol [120]; i.v. nefopam [121]; i.v. ketamine [121–123]; p.o. nimodipine [124]; i.v. dexmedetomidine [125]; i.v. duloxetine [126]; i.v. methyl-prednisolone [127]; and i.v. magnesium [128].

Twenty-four interventions were administered together with a basic analgesic regimen (S4 Appendix).

The risk of bias was low in 12 trials, unclear in 25, and high in four.

Nine trials did not demonstrate a significant effect on opioid consumption and/or pain scores: Clonidine, tramadol or buprenorphine added to FNB [92, 93, 95]; continuous lumbar plexus block [103]; morphine, ketamine or epinephrine added to intraarticular local anaesthetics [104, 106, 107]; betamethasone added to periarticular local anaesthetics [114]; and i.v. magnesium [128]. The remaining trials demonstrated statistically significant analgesic effects. Four trials demonstrated a statistically significant effect on opioid-related adverse events: Sciatic nerve block a reduction in PONV [97, 98], dexmedetomidine a reduction in PONV and pruritus [125], and epidural analgesia with ropivacaine and morphine an increase in pruritus [115] (Table 9).

**Table 9:** Qualitative analysis of other interventions. Treatment effects and adverse events are presented as results in control group → intervention group. FNB: femoral nerve block. PCA: patient controlled analgesia. LIA: local infiltration analgesia. PACU: postoperative care unit.

Author	Treatment intervention	Treatment effect on analgesic consumption	Highest pain level in a control group (VAS 0–100) (assay sensitivity)	Treatment effect on pain scores at 6 h postoperative	Treatment effect on pain scores at 24 h postoperative	Length of hospital stay in days	PONV, events registration time	Sedation, events registration time	Dizziness, events registration time	Pruritus, events registration time
Andersen, H. L. (2013) [88]	Saphenous nerve block ropivacaine 0.75% 15 mL administered 3 times: in the PACU, 8:00 PM and 8:00 AM	PCA i.v. morphine day of surgery 36 → 23 mg, *p = 0*.*28 NS* Total number of FNB catheter ropivacaine boluses 2 → 0 *NS*	40 mm at 6 h rest	40 → 20 mm at rest (8pm POD0) *p<0*.*05*	30 → 20 mm at rest (8pm POD0) *NS p>0*.*05*	3.1 → 3.1 *NS*	3/20 → 2/20 POD1 *NS*	-	-	-
Jenstrup, M. T. (2012) [89]	Adductor canal block ropivacaine 0.75% 30 ml immediately postoperative and additional 15 ml at 6, 12 and 18 h postoperatively.	PCA i.v. morphine 0–24 h 56 → 40 mg *p = 0*.*006*	70 mm at 6 h movement	42 → 38 mm at rest *NS p>0*.*05*. 70 → 66 mm at movement *p = 0*.*01*	21 → 13 mm at rest *p = 0*.*01*. 58 → 39 mm at movement *p = 0*.*01*	-	19/37 → 8/34 0–24 h *NS*	-	-	-
Krishnan, SH. (2016) [90]	Adductor canal block bupivacaine 30 mL 0.25% ± buprenorphine 0.2 mg	Oral hydrocodone equivalents 0–24 h, 35.8 → 25.3 mg, NS *p = 0*.*0076*	-	-	-	-	9/49 → 13/48 0–24 h *NS* p = 0.305	-	-	1/49 → 1/48 0–24 h *NS* p = 0.747
Shah, N. A. (2015) [91]	Adductor canal block ropivacaine 0.75% 30 mL ± continuous ropivacaine 0.25% 30 mL every 4 h 0–24 h	I.m. tramadol 0–24 h 5 → 0 mg *NS p>0*.*05*	27 mm at 24 h rest	27 → 21 mm at rest *p<0*.*001*	27 → 21 mm at rest *p<0*.*001*	3.2 → 3.1 *NS p = 0*.*157*	1/39 → 1/46 0–48 h *NS*	-	-	-
Casati, A. (2005) [92]	FNB ropivacaine 0.75% 25 mL ± clonidine 1 microgram/kg (group 1). Continuous ropivacaine 0.2% ± clonidine 1 microgram/mL (group 2)	PCA continuous FNB POD1: 170 → 169 (group 1) and 164 mL (group 2) *p = 0*.*51 NS*. Patients requiring i.v. fentanyl. 14 → 10 (both groups) *NS*	30 mm at 24 h movement	12 → 18 (group 1) and 26 mm (group 2) at rest *NS p>0*.*05*. 19 → 26 (group 1) and 26 mm (group 2) at movement *NS p>0*.*05*	15 → 26 (group 1) and 26 mm (group 2) at rest *NS p>0*.*05*. 30 → 38 (group 1) and 37 mm (group 2) at movement *NS p>0*.*05*	-	4/19 → 6/20 and 5/19 0–48 h *NS*	-	-	-
Ekmekci, P. (2010) [93]	Continuous FNB ropivacaine 0.2% ± tramadol 1–2 mg/mL 0.1 mL/h for 48 h	I.m. diclofenac 0–24 h *NS*	21 mm at 6 h rest	21 → 16 (group 1) and 18 mm (group 2) at rest *NS p>0*.*05*	17 → 14 (group 1) and 6 mm (group 2) at rest *NS p>0*.*05*	-	-	-	-	-
Elmawgoud, A. A. (2008) [94]	FNB ropivacaine 0.2% 30 mL ± fentanyl 4 mikrog/mL (group 1) ± magnesium (group 2)	PCA i.v. morphine 0–24 h 33.3 → 17.3 (group 1) and 16.5 mg (group 2) *p<0*.*05*	50 mm at 24 h rest	30 → 20 mm at rest (both groups) *p<0*.*05*	50 → 30 (group 1) and 20 mm (group 2) at rest *p<0*.*05*	-	-	-	-	-
Kosel, J. (2015) [95]	FNB bupivacaine 0.25% with epinephrine 0.5 mL/kg ± buprenorphine 0.3 mg	I.m. morphine 0–24 h 12.5 → 10 mg *NS p = 0*.*4*	40 mm at 24 h rest	-	40 → 36 mm at rest *NS p = 0*.*57*	10.3 → 10.7 *NS p = 0*.*61*	-	-	-	-
McNamee, D. A. (2001) [96]	Combined femoral and sciatic block with bupivacaine (group 1) / ropivacaine (group 2) 2 mg/kg divided equally between femoral and sciatic nerves	PCA i.v. morphine 0–24 h 36 → 14 (group 1) and 19 mg (group 2) *p<0*.*05*	29 mm at 24 h movement	15 → 0 (group 1) and 5 mm (group 2) at movement *p<0*.*05* (group 1) *p>0*.*05* (group 2)	29 → 17 (group 1) and 29 mm (group 2) at movement *p<0*.*05* (group 1) *p>0*.*05* (group 2)	-	-	-	-	-
Abdallah, F. W. (2014) [97]	Proximal (group 1) / distal (group 2) sciatic nerve block of 2:1 bupivacaine 0.5% and lidocaine 2% 30 mL with epinephrine	Oral morphine equivalents 0–24 h 167 → 131 (group 1) and 128 mg (group 2) *p = 0*.*01*	62 mm at 6 and 24 h movement	48 → 28 (group 1) and 15 mm (group 2) at rest 53 → 28 (group 1) and 35 mm (group 2) at movement *NS p>0*.*05*	35 → 35 (group 1) and 40 mm (group 2) at rest 63 → 60 (group 1) and 58 mm (group 2) at movement *NS p>0*.*05*	-	13/18 → 5/17 and 4/18 0–24 h *p = 0*.*005*	-	-	-
Cappelleri, G. (2011) [98]	Continuous lumbar plexus block levobupivacaine 0.125% 8 mL/h ± Continuous sciatic nerve block levobupivacaine 0.06% 0.1mL/kg	PCA i.v. morphine 0–24 h 4.6 → 2.3 mg *p<0*.*01*	29 mm at 24 h movement	14 → 15 mm at rest *NS p>0*.*05*. 10 → 10 mm at movement *NS p>0*.*05*	21 → 8 mm at rest *NS p>0*.*05*. 29 → 10 mm at movement *p<0*.*05*	-	7/18 → 1/19 0–48 h *p = 0*.*013*	-	-	1/18 → 0/19 0–48 h *NS*
Martinez Navas, A. and M. Echevarria Moreno (2006) [99]	Sciatic nerve block ropivacaine 0.5% 20 mL ± Continuous ropivacaine 0.2% 5 mL/h	S.c. morphine 0–24 h *NS*	52 mm at 24 h movement	17 → 9 mm at rest. 21 → 11 mm at movement *NS p>0*.*05*	25 → 9 mm at rest *NS p>0*.*05*. 52 → 11 mm at movement *p<0*.*05*	-	0/10 → 0/7 Unclear *NS*	-	-	-
Sato, K. (2014) [100]	Sciatic nerve block ropivacaine 0.2% 20 mL± Continuous ropivacaine 0.2% 5 mL/h	PCA i.v. morphine 0–24 h 7.3 → 4 mg *NS p = 0*.*057*	32 mm at 24 h rest	22 → 19 mm at rest *NS p>0*.*05*	32 → 12 mm at rest *p<0*.*05*	23 → 25 *NS*	6/30 → 5/30 0–48 h *NS*	-	-	-
McNamee, D. A. (2002) [101]	Obturator nerve block ropivacaine 0.75% 5 mL Femoral and sciatic nerve block ro-pivacaine 0.75%15 mL each nerve	PCA i.v. morphine 0–24 h 45 → 32 mg *p<0*.*05*	25 mm at 24 h movement	7 → 5 mm at movement *NS p>0*.*05*	25 → 28 mm at movement *NS p>0*.*05*	-	-	-	-	-
Runge, C. (2016) [102]	Obturator nerve block bupivacaine 46 mg, clonidine 0.0375 mg, dexamethasone 2 mg, epinephrine	PCA i.v. morphine 0–24 h 20 → 2 mg *p = 0*.*0007*	40 mm at 6 h rest	20 → 5 mm at rest *p>0*.*05*. 40 → 15 mm at movement *p<0*.*025*	19 → 0 mm at rest. 30 → 18 mm at movement *p<0*.*025*	*NS*	*NS*	-	-	-
Frassanito, L. (2009) [103]	Single lumbar plexus block ropivacaine 0.6% 30 mL. Single sciatic block ropivacaine 0.6% 15 mL ± Continuous lumbar plexus infusion of ropivacaine 0.2% 10 mL/h for 48 h	I.v. tramadol 0–72 h 236 → 185 mg *NS p = 0*.*06*	50 mm at 24h rest	0 → 5 mm at rest (unclear time for registration) *NS p>0*.*05*	50 → 38 mm at rest (unclear time for registration) *NS p>0*.*05*	-	3/22 → 1/22 0–48 h *NS*	-	-	-
Badner, N. H. (1997) [104]	Intraarticular injection ± bupivacaine 0.5% 30 mL (group 1) ± morphine 1 mg (group 2) with epinephrine	PCA i.v. morphine 0–24 h 68 → 58 (group 1) and 55 mg (group 2) *NS p>0*.*05*	63 mm at 6 h rest	63 → 60 (group 1) and 55 mm (group 2) at rest *NS p>0*.*05*	52 → 51 (group 1) and 45 mm (group 2) at rest *NS p>0*.*05*	-	-	-	-	-
Garcia, J. B. (2010) [105]	Intraarticular 10 mg morphine in 20 mL	S.c. morphine 0–24 h 20.6 → 12.2 mg *p = 0*.*0001*	80 mm at 6 h rest	80 → 50 mm at rest *p = 0*.*006*	20 → 20 mm at rest *NS p>0*.*05*	-	7/25 → 7/25 0–24 h *NS*	4/25 → 4/25 0–24 h *NS*	-	-
Guara Sobrinho, H. (2012) [106]	Intraarticular ketamine 0.25 (group 1) / 0.5 (group 2) mg/kg in 20 mL	I.v. morphine 0–24 h 14.5 → 14.9 (group 1) and 14 mg (group 2) *NS p = 0*.*52*	55 mm at 6 h rest	55 → 48 (group 1) and 58 mm (group 2) at rest *NS p = 0*.*68*	32 → 31 (group 1) and 31 mm (group 2) at rest *NS p = 0*.*76*	-	7/20 → 8/19 and 6/17 0–24 h *NS*	4/20 → 1/19 and 4/17 0–24 h *NS*	1/20 → 3/19 and 5/17 0–24 h *NS*	-
Schotanus, M. G. (2015) [107]	Intracapsular LIA ropivacaine 2% 150 mL ± epinephrine 0.01%	-	34 mm at 24 h rest	26 → 28 mm at rest *NS p>0*.*05*	34 → 29 mm at rest *NS p>0*.*05*	-	-	-	-	-
Ali, A. (2015) [108]	Continuous intraarticular infusion of ropivacaine 15 mg/h for 48 h	Oxycodone 0–72 h 25 → 20 mg *NS p = 0*.*06*	40 mm at 24 h rest	21 → 14 mm at rest (8pm) *p = 0*.*05*	40 → 33 mm at rest (day 1 at 12 noon) *p = 0*.*02*	4.1 → 4.1 *NS p = 0*.*8*	*NS*	-	-	-
Gomez-Cardero, P. and E. C. Rodriguez-Merchan (2010) [109]	Continuous intraarticular infusion of ropivacaine 0.2% 5 mL/h, cumulated 300 mL	Patients requiring supplementary analgesia 38 → 14% *p<0*.*05*	57 mm at 24 h rest	-	57 → 38 mm at rest *p<0*.*001*	7.3 → 5.7 *p<0*.*001*	-	-	-	-
Williams, D. (2013) [110]	Continuous intraarticular infusion of bupivacaine 0.5% 2 mL/h for 48 h	PCA i.v. morphine 0–24 h 19.2 → 13.8 mg NS *p = 0*.*58*	31 mm at 6 h rest	31 → 24 mm at rest *NS p = 0*.*0428*	21 → 17 mm at rest *NS p = 0*.*0386*	3.9 → 4.7 *NS p = 0*.*155*	3/25 → 1/24 *NS*	12/25 → 9/24 0–24 h *NS*	-	0/25 → 1/24 0–24 h *NS*
Andersen, K. V. (2013) [111]	Intraoperative LIA ropivacaine 300 mg ± ketorolac 30 mg. Postoperative intraarticular ropivacaine 100 mg and ketorolac 15 mg every 6h	PCA i.v. morphine 0–24 h 25 → 5 mg *p<0*.*0001*	64 mm at 24 h movement	29 → 6 mm at rest (2–6 h mean values) *p = 0*.*0003* 29 → 19 mm at rest *p<0*.*002*	40 → 12 mm at rest (6–24 h mean values) *p = 0*.*0001* 64 → 29 mm at rest *p<0*.*0001*	3 (3–3) → 3 (2–3) median interquartile range *p = 0*.*02*	*NS*	-	-	*NS*
Sean, V. W. (2011) [112]	Periarticular bupivacaine 0.5% 0.5 mL/kg with epinephrine ± Deep tissue triamcinolone acetonide (corticosteroid) 40 mg	PCA i.v. morphine 0–24 h 1.3 → 0.9 mg *p = 0*.*03*	18 mm at 6 h rest	18 → 17 mm at rest *NS p>0*.*05*	14 → 12 mm at rest *NS p>0*.*05*	6.8 → 5.2 *p = 0*.*02*	-	-	-	-
Tsukada, S. (2016) [113]	Periarticular ropivacaine 300 mg with morphine 8 mg, ketoprofen 50 mg, epinephrine ± methylprednisolone 40 mg	Diclofenac consumption on the night of surgery *NS*	26 mm at 24 h rest	3 → 2 mm at rest *NS p = 0*.*8*	26 → 17 mm at rest *NS p = 0*.*057*	-	1/37 → 2/38 night of surgery *NS* p = 0.57	-	-	0/37 → 1/38 night of surgery *NS* p = 0.31
Yue, D. B. (2013) [114]	Periarticular ropivacaine 0.75% 30 mL with epinephrine ± betamethasone 1 mL	PCA i.v. morphine day 1 13.5 → 13 mg *NS p>0*.*05*	82 mm at 24 h movement	43 → 41 mm at rest *NS p>0*.*05*	63 → 62 mm at rest *NS p>0*.*05*. 82 → 83 mm at movement *NS p>0*.*05*	-	-	-	-	-
Axelsson, K. (2005) [115]	Epidural initiated in the PACU: ropivacaine 1.25 (group 1) / 2 (group 2) mg/mL + morphine 0.02 mg/mL, 8 mL/h.	PCA i.v. morphine 0–24 h 45.8 → 21.8 (group 1) and 8.2 mg (group 2) *p<0*.*001 and p<0*.*0001*	59 mm at 24 h movement	33 → 35 (group 1) and 4 mm (group 2) at rest 58 → 46 (group 1) and 9 mm (group 2) at movement p>0.05 (group 1) p<0.01 (group 2)	21 → 16 (group 1) and 9 mm (group 2) at rest *p>0*.*05* 59 → 42 (group 1) and 22 mm (group 2) at movement p>0.05 (group 1) p<0.01 (group 2)	-	3/15 → 4/15 and 2/15 Unclear *NS*	-	1/15 → 3/15 and 1/15 Unclear *NS*	1/15 → 8/15 and 8/15 Unclear *p<0*.*05*
Daabiss, M. A. and A. Kandil (2013) [116]	Epidural bolus bupivacaine 0.5% 1 mL ± magnesium sulphate 50 mg and infusion 10 mg/h (group 1) / ± midazolam 0.05 mg/kg (group 2)	PCEA fentanyl 0–24 h 321 → 220 (group 1) and 256 microgram (group 2) *p<0*.*05* I.m. pethidine 0–24 h 92 → 53 (group 1) and 70 mg (group 2) *p<0*.*05*	5 mm at 6 and 24 h rest	5 → 3 (group 1) and 4 mm (group 2) at rest *NS p>0*.*05*	5 → 5 (group 1) and 5 mm (group 2) at rest *NS p>0*.*05*	-	3/40 → 1/40 and 2/40 0–24 h *NS*	0/40 → 0/40 and 2/40 0.24 h *NS*	-	-
Hendolin, H. (1996) [117]	Group 1: I.m. morphine 0.14 mg/kg 1 h preoperative. Epidural morphine 4 mg at 0 h and 3 mg at 10 h postoperative. Group 2: Epidural morphine 4 mg at 0 h and 3 mg at 10 h postoperative. Group 3: I.m. morphine 0.14 mg/kg 1 h preoperative	PCA i.v. fentanyl 0–20 h 0.82 → 0.46 (group 1), 0.39 (group 2) and 0.79 mg/kg (group 3) *p<0*.*01 (group 1 and 2)*, *p>0*.*05 (group 3)*	48 mm at 6 h rest	48 → 26 (group 1), 27 (group 2) and 25 mm (group 3) at rest *p<0*.*001*	23 → 17 (group 1), 23 mm (group 2) and 35 mm (group 3) at rest *NS p>0*.*05*	-	5/11 → 6/10, 6/10 and 4/10 0–20 h *NS*	*NS*	-	0/11 → 3/10, 5/10 and 2/10 0–20 h *NS*
Abrisham, S. M. (2014) [118]	Transdermal fentanyl patch 4.2 mg/patch	PCA i.v. morphine 0–72 h 40 → 33 mg *p = 0*.*01*	67 mm at 6 h rest	67 → 54 mm at rest *p = 0*.*035*	57 → 37 mm at rest *p = 0*.*002*	-	9/20 → 5/20 Unclear *NS*	-	-	2/20 → 4/20 Unclear *NS*
Sathitkarnmanee, T. (2014) [119]	Transdermal fentanyl patch 50 microgram/h constituted 10–12 h before surgery	PCA i.v. morphine 0–24 h 24.9 → 15.4 mg *p = 0*.*001*	64 mm at 6 h movement	46 → 27 mm at rest (mean score 0–48 h) *p = 0*.*002* 64 → 44 mm at movement (mean score 0–48 h) *p = 0*.*002*	-	-	-	-	-	-
Stiller, C. O. (2007) [120]	I.v. tramadol 100 mg x 4	PCA i.v. morphine 0–24 h 72 → 51 mg *p<0*.*05*	63 mm at 6 h rest	60 → 63 mm at rest *NS p>0*.*05*	-	-	15/32 → 11/31 0–24 h *NS*	13/32 → 9/31 0-24h *NS*	-	-
Aveline, C. (2009) [121]	I.v. nefopam (group 1) / ketamine (group 2) 0.2 mg/kg after induction, 0.12 mg/kg/h during surgery followed by 0.06 mg/kg/h until POD2	PCA i.v. morphine 0–24 h 56.8 → 39.3 (group 1) and 39.2 mg (group 2) *p<0*.*0001*	60 mm at 6 h movement	41 → 38 (group 1) and 34 mm (group 2) at rest 60 → 58 (group 1) and 56 mm (group 2) at movement *NS p>0*.*05*	36 → 37 (group 1) and 23 mm (group 2) at rest *p<0*.*005 (group 2)* 55 → 47 (group 1) and 50 mm (group 2) at movement *NS p>0*.*05*	14.1 → 13 (group 1) and 12 (group 2) *NS*	9/24 → 7/24 and 4/25 0–48 h *NS*	0–48 h *NS*	-	-
Adam, F. (2005) [122]	Ketamine 0.5 mg/kg bolus followed by 0.003 mg/kg/min during surgery and 0.0015 mg/kg/min after surgery	PCA i.v. morphine 0–48 h 69 → 45 mg *p<0*.*02*	33 mm at 24 h rest	28 → 26 mm at rest *NS p>0*.*05*	33 → 29 mm at rest *NS p>0*.*05*	11 → 11 *NS*	3/20 → 2/20 Unclear *NS*	0/20 → 0/20 Unclear *NS*	-	-
Cengiz, P. (2014) [123]	Intraoperative i.v. ketamine 6 microgram/kg/minute until closure	PCA i.v. morphine 0–24 h 85.2 → 47 mg *p<0*.*001*	21 mm at 6 h rest	21 → 9 mm at rest *p<0*.*001*	6 → 2 mm at rest *p<0*.*001*	-	5/30 → 1/30 0–24 h *NS*	-	-	-
Casey, G. (2006) [124]	Oral nimodipine 90 mg 1 h preoperative and 30 mg x 4 postoperative	PCA i.v. morphine 0–24 h 45 → 62 mg *p = 0*.*02*	57 mm at 6h rest	50 → 47 mm at rest 57 → 58 mm at movement *NS p>0*.*05*	34 → 23 mm at rest 51 → 45 mm at movement *NS p>0*.*05*	-	8/20 → 7/20 Unclear *NS*	-	-	-
Chan IA. (2016) [125]	I.v. dexmedetomidine 0.5 microg/kg bolus and 0.5 microg/kg/h infusion during surgery	PCA i.v. morphine 0–24 h 61.2 → 29.2 mg NS *p<0*.*001*	50 mm at 24 h rest	50 → 44 mm at rest *NS p = 0*.*41*	50 → 48 mm at rest *NS p = 0*.*79*	-	7/20 → 1/20 0–24 h p = 0.005	-	-	6/20 → 1/20 0–24 h *p = 0*.*015*
Ho, K. Y. (2010) [126]	Oral duloxetine 60 mg 2 h before surgery and the morning of POD1	PCA i.v. morphine 0–24 h 19.8 → 12.9 mg *p = 0*.*039*	50 mm at 24 h movement	10 → 10 mm at rest 25 → 20 mm at movement *NS p>0*.*05*	10 → 10 mm at rest 50 → 20 mm at movement *NS p>0*.*05*	-	5/24 → 3/23 Unclear *NS*	3/24 → 0/23 Unclear *NS*	3/24 → 2/23 Unclear *NS*	1/24 → 0/23 Unclear *NS*
Lunn, T. H. (2011) [127]	I.v. solumedrol 125 mg just before spinal anesthesia	Patients requiring sufentanil 0–24 h 10 → 2 *p<0*.*05*. Oral oxycodone 0–24 h, 20 → 10 mg *p<0*.*05*	70 mm at 24 h movement	34 → 17 mm at rest 65 → 16 mm at movement *p<0*.*01*	47 → 19 mm at rest 70 → 27 mm at movement *p<0*.*01*	2 → 2 *NS*	4/24 → 2/24 0–24 h *NS*	-	-	-
Frassanito, L. (2015) [128]	I.v. magnesium 40 mg/kg bolus and 10 mg/kg/h during surgery	PCA i.v. morphine 0–24 h, 14.4 → 13.9 mg NS *p = 0*.*4*	30 mm at 24 h rest	0 → 0 (average of rest and movement) *NS p>0*.*05*	30 → 25 (average of rest and movement) *NS p>0*.*05*	-	3/20 → 6/20 0–24 h *NS*	-	-	2/20 → 4/20 0–24 h *NS*

## Discussion

We have reviewed randomized controlled trials regarding postoperative analgesia after TKA, and have demonstrated analgesic effects in meta-analyses for single injection- and continuous FNB, intrathecal morphine, LIA, intraarticular injection of local anaesthetics, NSAIDs/COX-2 inhibitors, and gabapentinoids; and furthermore in stand-alone trials for a number of different interventions, according to the PRISMA checklist (S26 Appendix). By conducting meta-analyses we have enhanced the evidence to the highest level possible with the present trials. While this sounds promising, the quality of evidence throughout the included data is discouragingly low due to uncertain or high risk of bias, low sample size in trials and meta-analysis interventions, heterogeneous results, and low assay sensitivity. These findings are similar to the results in our recent systematic review on pain management after THA [2]. Consequently, we have demonstrated that no optimal strategy for postoperative pain treatment after TKA exist in the literature.

The accepted level of pain varies in the analyzed material. In some trials no basic analgesic regimens were provided and high pain scores were accepted, whereas in other trials acetaminophen, NSAIDs, gabapentin, and even FNB were administered as adjuncts to the intervention. In these trials both intervention- and control groups tended to have lower pain scores. These differences lead to a considerable variance in assay sensitivity amongst trials. The wide variation in trial-setup may be accounted for by cultural or tradition based differences in the approach to analgesic treatment, e.g. the propensity to apply invasive procedures or the general pain threshold.

### Interpretation of meta-analysed interventions

For oral treatments we analyzed two subgroups: NSAIDs/COX-2-inhibitors and gabapentinoids.

The included trials in the NSAID subgroup were characterized by low assay sensitivity, which contributes to a low absolute effect. However, the intervention provided a small but statistically significant effect on pain scores and morphine consumption. The adverse event profile of NSAIDs is controversial and short follow up periods in randomized pain trials in general may be problematic for the detection hereof (17). However, the meta-analyses did not demonstrate an increased risk of adverse effects which is supported by similar results in the review regarding THA [2].

The evidence regarding gabapentinoids was even less convincing, with insignificant results partially due to a low number of included trials.

Four meta-analysed interventions investigated procedure specific local anaesthetic interventions: single FNB, continuous FNB, LIA, and intraarticular injection of local anaesthetics. When reviewing the outcomes, intraarticular injection tended to be inferior compared to the other interventions.

Single FNB performed slightly better in two out of three primary outcomes compared to continuous FNB. The strength of evidence in TSA was generally high for both interventions. Continuous FNB is a more invasive, time consuming and for the patient cumbersome procedure due to the postoperative catheterization.

Single FNB and LIA provided equally satisfying analgesia. Both procedures demonstrated a relevant reduction in morphine consumption, pain scores and PONV. A recent systematic review of trials comparing LIA to FNB after TKA reported a small insignificant difference in analgesic effect favoring LIA [129]. The current evidence does not allow designation of a superior intervention amongst the two, but the well-known risk of motor blockade with FNB may render this method less attractive [130]. It should be noted that different combinations of drugs and dosages were administered for both FNB and LIA. Pinpointing optimal analgesics regimens for FNB and LIA are imperative for designation of a superior intervention.

The meta-analysis of intrathecal morphine demonstrated some analgesic effect on morphine consumption and pain scores, but a rather large increase in morphine related adverse events.

### Strengths and limitations

The large amounts of data in this review were manually typed with more than 12.000 separate boxes in Excel^®^, creating a major potential for typing errors. To minimize this risk data were analyzed and registered by two independent authors with prior data extraction experience, and subsequently compared.

In a considerable number of trials data were presented as medians and range/IQR, likely because of a skewed distribution. Treating data as normally distributed by converting to mean/SD was necessary, but nonetheless a limitation.

About half of the corresponding authors replied our emails regarding bias. This resolved 74 unclear domains and altered the total number of trials with low summarized risk of bias from six to 15. This is still only 13% of all trials. For trials included in meta-analyses this proportion was 7%, which is problematic as the quality of the meta-analyses is partially limited by the quality of the trials. The majority of trials had an unclear summarized risk of bias. We believe that in most of these trials, relatively few and easily attainable measures would be required to improve this risk from unclear to low, especially if authors had access to a standardized postoperative pain trial protocol that took into account the pitfalls leading to high or unclear risk of bias.

Opioid consumption and pain intensity are associated, hence both outcomes require assessment. Whether opioid consumption and pain should be calculated as absolute or relative differences between treatment and control groups, or as the number of patients with a predefined level of pain, is controversial. In this review we chose to report effects as absolute (mean) differences, which may be arguable.

The majority of included interventions each provided acceptable levels of analgesia, however it is probably reasonable to keep postoperative analgesic treatment to a limited number of interventions. Each additional intervention added to the standard postoperative analgesic regimen may increase the risk of adverse effects or events [131]. Regarding invasive procedures we must consider the risk of inducing severe adverse effects and the time consumption by qualified personnel such as doctors. Furthermore, we know little about the effect of combining different analgesic interventions after TKA [132]. Thus, the absolute analgesic effect may decline for each additional analgesic, because different interventions may affect the same analgesic pathways, and because the analgesic potential is probably lower when pain levels are already reduced by other analgesics.

In conclusion, no gold treatment for pain treatment after TKA exists in the literature. The GRADE rated recommendations varied from very low to moderate (except for one high) for the different interventions. High or unclear risk of bias, heterogeneity of trial designs, and the small trial sample sizes, are challenges in designation of a best proven optimal postoperative analgesic regimen for TKA. A way to overcome these challenges may be to establish standard research guidelines regarding postoperative pain management, and focus on conducting high quality upscale trials.

## Supporting information

S1 Appendix Search strategy. (PDF)

S2 Appendix Opioid conversion table used to calculate i.v. morphine equivalents. (PDF)

S3 Appendix Excluded articles. (PDF)

S4 Appendix Detailed information about references related to specific outcomes. (PDF)

S5 Appendix Forest plot displaying mean difference in pain scores 6 hours postoperative at movement for each meta-analyzed intervention. Green squares with horizontal lines represent mean differences and 95% confidence intervals for each trial. Black tiles represent the mean difference of each intervention.
(PDF)

S6 Appendix Forest plot displaying mean difference in pain scores 24 hours postoperative at movement for each meta-analyzed intervention. Green squares with horizontal lines represent mean differences and 95% confidence intervals for each trial. Black tiles represent the mean difference of each intervention.
(PDF)

S7 Appendix Forest plot displaying risk ratio of postoperative nausea and vomiting for each meta-analyzed intervention. Blue squares with horizontal lines represent mean differences and 95% confidence intervals for each trial. Black tiles represent the mean difference of each intervention.
(PDF)

S8 Appendix Forest plot displaying risk ratio of postoperative sedation for each meta-analyzed intervention. Blue squares with horizontal lines represent mean differences and 95% confidence intervals for each trial. Black tiles represent the mean difference of each intervention.
(PDF)

S9 Appendix Forest plot displaying risk ratio of postoperative dizziness for each meta-analyzed intervention. Blue squares with horizontal lines represent mean differences and 95% confidence intervals for each trial. Black tiles represent the mean difference of each intervention.
(PDF)

S10 Appendix Forest plot displaying risk ratio of postoperative pruritus for each meta-analyzed intervention. Blue squares with horizontal lines represent mean differences and 95% confidence intervals for each trial. Black tiles represent the mean difference of each intervention.
(PDF)

S11 Appendix Forest plot displaying mean difference in length of stay for each meta-analyzed intervention. Green squares with horizontal lines represent mean differences and 95% confidence intervals for each trial. Black tiles represent the mean difference of each intervention.
(PDF)

S12 Appendix L’Abbé plots of trials concerning single femoral nerve block. Higher degrees of heterogeneity were demonstrated for pain at 6 and 24 hours rest and 24 hours movement. The size of the ball resembles the number of included patients in that trial and it is standardized across the different plots.
(PDF)

S13 Appendix Trial Sequential Analyses (TSA) for single femoral nerve block for morphine consumption and pain scores at 6 and 24 hours at rest and 24 hours at movement. A priori estimated information sizes (APIS) (333, 730, 321 and 560 patients, respectively) were based on an alpha-value of 0.05 and a beta-value of 0.9. The sensitivity to detect a mean difference for opioid consumption was predefined as 10 mg morphine equivalents 0–24 h postoperative and for pain scores a mean difference of 15 mm (VAS 0–100 mm). The blue line depicts the cumulative Z-score of the meta-analysis. The outer red lines illustrate the sequential z-score threshold for significance. The inner red lines illustrate the area of futility. The burgundy lines represent a stationary Z-score at 1.96 corresponding to p = 0.05. Threshold for significance was reached for morphine consumption and pain at 6 and 24 hours rest. Morphine consumption and pain score at 24 hours rest reached APIS, concluding that the intervention has an effect on these outcomes.
(PDF)

S14 Appendix L’Abbé plots of trials concerning continuous femoral nerve block. Homogeneity was demonstrated for morphine consumption and pain scores. The size of the ball resembles the number of included patients in that trial and it is standardized across the different plots.
(PDF)

S15 Appendix Trial Sequential Analyses (TSA) for continuous femoral nerve block for morphine consumption and pain scores at 6 and 24 hours at rest and 24 hours at movement. A priori estimated information sizes (APIS) (47, 268, 272 and 69 patients, respectively) were based on an alpha-value of 0.05 and a beta-value of 0.9. Threshold for significance and APIS were reached for morphine consumption and all pain scores concluding that continuous femoral nerve block has a positive effect on these outcomes. For further elaboration see S13 Appendix.
(PDF)

S16 Appendix L’Abbé plots of trials concerning intrathecal morphine. Moderate degrees of heterogeneity were demonstrated for morphine consumption and pain scores. The size of the ball resembles the number of included patients in that trial and it is standardized across the different plots.
(PDF)

S17 Appendix Trial Sequential Analyses (TSA) for intrathecal morphine for morphine consumption and pain scores at 6 and 24 hours at rest. A priori estimated information sizes (APIS) (183, 490 and 151 patients, respectively) were based on an alpha-value of 0.05 and a beta-value of 0.9. Morphine consumption reached the threshold for significance but not APIS. Pain score at 24 hours rest reached the boundary for futility and APIS concluding that there is no reason for further investigation of this outcome. For further elaboration see S13 Appendix.
(PDF)

S18 Appendix L’Abbé plots of trials concerning Local Infiltration Analgesia (LIA). Lower degrees of heterogeneity were demonstrated for morphine consumption and pain scores at rest. Moderate degrees were present for pain scores at movement. The size of the ball resembles the number of included patients in that trial and it is standardized across the different plots.
(PDF)

S19 Appendix Trial Sequential Analyses (TSA) for Local Infiltration Analgesia (LIA) for morphine consumption and pain scores at 6 and 24 hours at rest and at movement. A priori estimated information sizes (APIS) (635, 349, 149, 217 and 174 patients, respectively) were based on an alpha-value of 0.05 and a beta-value of 0.9. Threshold for significance and APIS were reached for all end-points, concluding that LIA has a positive effect on these outcomes. For further elaboration see S13 Appendix.
(PDF)

S20 Appendix L’Abbé plots of trials concerning intraarticular injection. Homogeneity was demonstrated for morphine consumption and pain at 6. Pain scores at 24 hours at rest were heterogeneous. The size of the ball resembles the number of included patients in that trial and it is standardized across the different plots.
(PDF)

S21 Appendix Trial Sequential Analyses (TSA) for intraarticular injection for morphine consumption and pain scores at 6 and 24 hours rest. A priori estimated information sizes (APIS) (88, 127 and 394 patients, respectively) were based on an alpha-value of 0.05 and a beta-value of 0.9. Threshold for significance and APIS were reached for morphine consumption and pain at 6 hours rest concluding that intraarticular injection has a positive effect on these outcomes. For further elaboration see S13 Appendix.
(PDF)

S22 Appendix L’Abbé plots of trials concerning NSAIDs/COX-2-inhibitors. Lower degrees of heterogeneity were demonstrated for morphine consumption and moderate degrees of heterogeneity for pain scores. The size of the ball resembles the number of included patients in that trial and it is standardized across the different plots.
(PDF)

S23 Appendix Trial Sequential Analyses (TSA) for NSAIDs/COX-2-inhibitors for morphine consumption and pain scores at 6 and 24 hours rest and 24 hours movement. A priori estimated information sizes (APIS) (115, 270, 32 and 166 patients, respectively) were based on an alpha-value of 0.05 and a beta-value of 0.9. Threshold for significance and APIS were reached for morphine consumption and pain at 6 and 24 hours rest concluding that NSAIDs and COX-2-inhibitors has a positive effect on these outcomes. Morphine consumption and pain at 24 hours rest reached APIS with the first trial. Threshold for futility and APIS were reached for pain at 24 h movement concluding that further testing of this end-point is futile. For further elaboration see S13 Appendix.
(PDF)

S24 Appendix L’Abbé plots of trials concerning gabapentinoids. Moderate degrees of heterogeneity was demonstrated for morphine consumption and pain at 24 hours rest. The size of the ball resembles the number of included patients in that trial and it is standardized across the different plots.
(PDF)

S25 Appendix Trial Sequential Analyses (TSA) for gabapentinoids for morphine consumption and pain scores at 6 hours at rest, 24 hours rest and 24 hours at movement. A priori estimated information sizes (APIS) (905, 132, 104, 147 and 108 patients, respectively) were based on an alpha-value of 0.05 and a beta-value of 0.9. Threshold for significance and APIS were reached for pain at 6 and 24 hours at movement concluding that gabapentinoids have a positive effect on these outcomes. Threshold for futility and APIS were reached for pain at 6 and 24 h at rest concluding that further testing of these end-points is futile. For further elaboration see S13 Appendix.
(PDF)

S26 Appendix PRISMA checklist. (DOC)
