# Supplementary material for: Postoperative pain treatment after total knee arthroplasty: A systematic review
Source: PLoS One. 2017 Mar 8;12(3):e0173107. doi: 10.1371/journal.pone.0173107 (PMC5342240; doi:10.1371/journal.pone.0173107)
Supplement: S1 Appendix — (PDF) [file pone.0173107.s001.pdf]

Search strategies for total knee arthroplasty

Total number of hits: 13578
Number of duplicates removed: 4952
Number of references for final list: 8626

MEDLINE (5126 hits)

1. [Title/Abstract]: pain or analgesia or anaesthesia or anesthesia or treatment or analgesics
2. [Title/Abstract]: knee arthroplasty or knee replacement or tka or knee surgery
3. [Title/Abstract]: postoperative or post or operative or following or perioperative or peri
4. #1 and #2 and #3
5. All fields: Post operative pain knee arthroplasty
6. limit #4 to clinical trials
7. #5 or #6

This search includes the following Mesh Terms: pain, analgesia, anesthesia, therapeutics, knee, arthroplasty, and postoperative period.

EMBASE (Ovid SP) (5806 hits)

1. [Title/Abstract]: pain or analgesia or anaesthesia or anesthesia or treatment or analgesics
2. [Title/Abstract]: knee arthroplasty or knee replacement or tka or knee surgery
3. [Title/Abstract]: postoperative or post or operative or following or perioperative or peri
4. #1 and #2 and #3
5. [Keyword]: pain or analgesia
6. [Keyword]: knee arthroplasty or knee replacement
7. #5 and #6
8. #4 or #7

This search includes the following Keywords: pain, analgesia, knee arthroplasty, and knee replacement

Cochrane Central Register of Controlled Trials (CENTRAL) in the Cochrane Library (2646 hits)

1. [Title/Abstract/Keyword] pain or analgesia or anaesthesia or anesthesia or treatment or analgesics
2. [Title/Abstract/Keyword]: knee arthroplasty or knee replacement or tka or knee surgery
3. [Title/Abstract/Keyword]: postoperative or post or operative or following or perioperative or peri
4. #1 and #2 and #3
5. [All fields]: Post operative pain knee arthroplasty
6. MeSH descriptor: [Pain] explode all trees AND MeSH descriptor: [Analgesia] explode all trees AND MeSH descriptor: [Arthroplasty, Replacement, Knee] explode all trees
7. #4 or #5 or #6
8. limit #7 to clinical trials

This search includes the following Mesh Terms: pain, analgesia, and Arthroplasty, Replacement Knee.
