# Supplementary material for: Postoperative pain treatment after total knee arthroplasty: A systematic review
Source: PLoS One. 2017 Mar 8;12(3):e0173107. doi: 10.1371/journal.pone.0173107 (PMC5342240; doi:10.1371/journal.pone.0173107)
Supplement: S2 Appendix — (PDF) [file pone.0173107.s002.pdf]

1mg morphine oral	0.33 mg morphine i.v. *
1mg fentanyl i.v.	100 mg morphine i.v.*
1mg oxycodone i.v.	1.33 mg morphine i.v. *
1mg oxycodone oral	0.5 mg morphine i.v. *
1mg tramadol oral	0.07 mg morphine i.v. *
1mg ketobemidone i.v.	1 mg morphine i.v. *
1mg ketobemidone oral	0.67 mg morphine i.v. *
1mg sufentanil i.v.	1000 mg morphine i.v. *
1mg Hydromorphine i.v.	6.67 mg morphine i.v. **
1mg meperidine i.v.	0.13 mg morphine i.v. **

* Akut smerte (Acute pain) application, Ph.D. Ole Mathiesen, Rigshospitalet Copenhagen

** http://www.globalrph.com/narcotic.cgi
