# Supplementary material for: Postoperative pain treatment after total knee arthroplasty: A systematic review
Source: PLoS One. 2017 Mar 8;12(3):e0173107. doi: 10.1371/journal.pone.0173107 (PMC5342240; doi:10.1371/journal.pone.0173107)
Supplement: S3 Appendix — (PDF) [file pone.0173107.s003.pdf]

Excluded studies and reason for exclusion: **1**: Not full reported trials, **2**: Included other surgery than TKA, **3**: Partial or bilateral TKA, revision TKA or fracture surgery, **4**: no relevant pain related outcome reported, **5**: Not controlled or randomized, **6**: Other reasons	
Adis Medical Writers (2014). "Manage pain before, during and after total knee arthroplasty using a multimodal approach to analgesia." Drugs and Therapy Perspectives 30(9): 321-324.	1: Summary clinical trial
Aguirre, J., et al. (2009). "The sciatic nerve should be blocked to optimize postoperative analgesia after knee arthroplasty." Eur J Anaesthesiol 26(5): 439-440.	1: Correspondance
Ahdieh, H., et al. (2004). "Efficacy of oxymorphone extended release in postsurgical pain: a randomized clinical trial in knee arthroplasty." J Clin Pharmacol 44(7): 767-776.	6: Only patients with moderate to severe pain were included
Albrecht, E., et al. (2014). "Single-injection or continuous femoral nerve block for total knee arthroplasty?" Clin Orthop Relat Res 472(5): 1384-1393.	5: Intervention and control groups received different baseline concentration of bupivacaine
AlOweidi, A. S., et al. (2011). "Intravenous dexmedetomidine or propofol adjuvant to spinal anesthesia in total knee replacement surgery." Jordan Medical Journal 45(2): 174-183.	6: Did not assess postoperative pain, only duration of spinal anasthesia
Andersen, K. V., et al. (2012). "Comparison between ropivacaine local infiltration analgesia with ketorolac or placebo for total knee replacement surgery." Scandinavian Journal of Pain 3 (3): 196-197.	1: Summary clinical trial
Andersen, L. O., et al. (2010). "Analgesic efficacy of subcutaneous local anaesthetic wound infiltration in bilateral knee arthroplasty: a randomised, placebo-controlled, double-blind trial." Acta Anaesthesiol Scand 54(5): 543-548.	6: Bilateralt TKA with active LIA in one knee and placebo in the other knee. The study can not control a systemic effect
Andersen, L. O., et al. (2008). "High-volume infiltration analgesia in total knee arthroplasty: a randomized, double-blind, placebo-controlled trial." Acta Anaesthesiol Scand 52(10): 1331-1335.	6: Bilateralt TKA with active LIA in one knee and placebo in the other knee. The study can not control a systemic effect
Anderson, S. K. and B. A. al Shaikh (1991) Diclofenac in combination with opiate infusion after joint replacement surgery. Anaesthesia and Intensive Care 19, 535-538	2: Included THA
Badner, N. H., et al. (1991). "Low-dose bupivacaine does not improve postoperative epidural fentanyl analgesia in orthopedic patients." Anesth Analg 72(3): 337-341.	4: Did not report morphine consumption or 6 or 24 h pain scores
Bagry, H., et al. (2008). "Effect of a continuous peripheral nerve block on the inflammatory response in knee arthroplasty." Reg Anesth Pain Med 33(1): 17-23.	6: Postoperative opioid consumption not recorded for the control group. Opioid consumption was not a primary endpoint
Banssillon, P. V. (1988). "Femoral nerve block for postoperative analgesia after open knee surgery." Can J Anaesth 35(4): 439-440.	1: Letter to editor
Bayazit, E. G., et al. (2013). "Effect of epidural levobupivacaine and levobupivacaine with fentanyl on stress response and postoperative analgesia after total knee replacement." Int J Clin Pharmacol Ther 51(8): 652-659.	6: Basic regimen not controlable, since the intervention was administered as rescue as well
Beaupre, L. A., et al. (2012). "Impact of a preemptive multimodal analgesia plus femoral nerve blockade protocol on rehabilitation, hospital length of stay, and postoperative analgesia after primary total knee arthroplasty: a controlled clinical pilot study." ScientificWorldJournal 2012: 273821.	5: Not randomized. Pre-emptive multimodal regimen +/- femoral nerve block. Further some patients received a single-shot anterior sciatic nerve block but the distribution of these patients is unclear
Berge, R., et al. (2012). "Femoral nerve block after total knee arthroplasty: Comparison of single dose versus continuous infusion. Analgesia and functional recovery." European Journal of Anaesthesiology 29: 129-130.	1: Summary clinical trial
Bergeron, S. G., et al. (2009). "Functional outcome of femoral versus obturator nerve block after total knee arthroplasty." Clin Orthop Relat Res 467(6): 1458-1462.	6: Long term follow-up (6 and 12 months) of Kardash et al. 2007
Bhandari, M. (2004) Does perioperative oral rofecoxib therapy improve functional recovery after knee replacement surgery? CMAJ : Canadian Medical Association journal 170, 1540 DOI: 10.1503/cmaj.1040499	1: Comment on Buvanendran et al 2003
Buvanendran, A., et al. (2003). "Effects of perioperative administration of a selective cyclooxygenase 2 inhibitor on pain management and recovery of function after knee replacement: a randomized controlled trial." Jama 290(18): 2411-2418.	5: Uncontrollable postoperative regimen
Cañada, P. (1997) Post-surgical intra-articular analgesia in knee arthroplasty. Revista Española de Anestesiología y Reanimación 44, 209	1: Conference abstract
Capdevila, X., et al. (1999). "Effects of perioperative analgesic technique on the surgical outcome and duration of rehabilitation after major knee surgery." Anesthesiology 91(1): 8-15.	3: Included arthrolysis
Carvalho, R., et al. (2012). "Effect of a single shot sciatic nerve block combined with a continuous femoral block on pain scores after knee arthroplasty. A randomized controlled trial." British Journal of Anaesthesia 108: ii23-ii24.	1: Conference abstract
Chelly, J. E. (2013). "Is the continuous saphenous block the right technique for postoperative pain management after total knee replacement?" Reg Anesth Pain Med 38(5): 461.	1: Letter to editor
Chelly, J. E., et al. (2001). "Continuous femoral blocks improve recovery and outcome of patients undergoing total knee arthroplasty." J Arthroplasty 16(4): 436-445.	5: Cohort study on continuous femoral infusion
Chen, Y., et al. (2009). "Efficacy and safety of an intra-operative intra-articular magnesium/ropivacaine injection for pain control following total knee arthroplasty." J Int Med Res 37(6): 1733-1741.	6: Retracted study

Excluded studies and reason for exclusion: **1**: Not full reported trials, **2**: Included other surgery than TKA, **3**: Partial or bilateral TKA, revision TKA or fracture surgery, **4**: no relevant pain related outcome reported, **5**: Not controlled or randomized, **6**: Other reasons		
Chen, Y. (2012). **"Erratum to:** Efficacy and safety of an intra-operative intra-articular magnesium/ropivacaine injection for pain control following total knee arthroplasty (J Int Med Res (2009)), 37, (1733-1741))." Journal of International Medical Research 40(5): 2031.	6:	Retracted study
Cheville, A., et al. (2001). "A randomized trial of controlled-release oxycodone during inpatient rehabilitation following unilateral total knee arthroplasty." J Bone Joint Surg Am 83-a(4): 572-576.	6:	Rehabilitation study commenced within seven days postoperative after TKA
Chinachoti, A. T., et al. (2012). "A randomized, double-blinded, placebo-controlled trial of periarticular infiltration of 0.25% bupivacaine for postoperative pain control after total knee." British Journal of Anaesthesia 108: ii396-ii397.	1:	Conference abstract
Christensen, C. P., et al. (2009). "Effect of periarticular corticosteroid injections during total knee arthroplasty. A double-blind randomized trial." J Bone Joint Surg Am 91(11): 2550-2555.	4:	Did not report morphine consumption or 6 or 24 h pain scores
Clarke, H., et al. (2014). "Perioperative gabapentin reduces 24 H opioid consumption and improves in-hospital rehabilitation but not post-discharge outcomes following total knee arthroplasty." Pain Research and Management 19 (3): e68.	1:	Conference abstract
Cook, P., et al. (2003). "Comparing the effects of femoral nerve block versus femoral and sciatic nerve block on pain and opiate consumption after total knee arthroplasty." J Arthroplasty 18(5): 583-586.	5:	Not randomized. Femoral +/- sciatic block determined for each participant by anesthetists' preference
Cooper, D. W. and G. Turner (1993). "Patient-controlled extradural analgesia to compare bupivacaine, fentanyl and bupivacaine with fentanyl in the treatment of postoperative pain." Br J Anaesth 70(5): 503-507.	2:	Did not include TKA
Dahl, J. B., et al. (1988). "Continuous blockade of the lumbar plexus after knee surgery--postoperative analgesia and bupivacaine plasma concentrations. A controlled clinical trial." Anaesthesia 43(12): 1015-1018.	2:	Did not include TKA
De La Paz-Estrada, C. and M. F. Perez (2008). "Comparative study of tramadol versus placebo in knee surgery under regional anesthesia. [Spanish]." Revista Mexicana de Anestesiologia 31(3): 179-183.	2:	Included other surgical procedures
Dereli, N., et al. (2011). "Intraarticular levobupivacaine injection for postoperative pain control in total knee replacement: With or without morphine?" Regional Anesthesia and Pain Medicine 2): E268.	1:	Conference abstract
Eggers, K. A., et al. (1999). "Effect of oral and i.v. tenoxicam in postoperative pain after total knee replacement." Br J Anaesth 83(6): 876-881.	4:	Did not report morphine consumption or 6 or 24 h pain scores
Essving, P., et al. (2009). "Reduced hospital stay, morphine consumption, and pain intensity with local infiltration analgesia after unicompartmental knee arthroplasty." Acta Orthop 80(2): 213-219.	3:	Only unicompartmental knee arthroplasties
Feng, Y., et al. (2008). "Effects of a selective cyclooxygenase-2 inhibitor on postoperative inflammatory reaction and pain after total knee replacement." J Pain 9(1): 45-52.	3:	Bilateral TKA
Ferrante, F. M., et al. (1993). "Regression of sensory anesthesia during continuous epidural infusions of bupivacaine and opioid for total knee replacement." Anesth Analg 77(6): 1179-1184.	5:	The intervention was used as PCEA and was not administered in a fixed dose. Did not assess postoperative pain scores or opioid consumption
Forster, J. G., et al. (2008). "Epinephrine 4 microg/mL added to a low-dose mixture of ropivacaine and fentanyl for lumbar epidural analgesia after total knee arthroplasty." Anesth Analg 106(1): 301-304, table of contents.	6:	Not controllable regimen. Pain scores were observed at specific times of the day and not at specific time intervals from the operation. Rescue medication: top-up epidural or oxycodone
Forster, J. G. and P. H. Rosenberg (2004). "Small dose of clonidine mixed with low-dose ropivacaine and fentanyl for epidural analgesia after total knee arthroplasty." Br J Anaesth 93(5): 670-677.	6:	Not a proper controllable regimen. Study medication was administered as PCA
Fu, P. L., et al. (2010). "Efficacy of a multimodal analgesia protocol in total knee arthroplasty: a randomized, controlled trial." J Int Med Res 38(4): 1404-1412.	6:	Multimodal analgesic protocol. Too many different drugs to control the intervention
Fujii, Y. and M. Nakayama (2005). "Effects of dexamethasone in preventing postoperative emetic symptoms after total knee replacement surgery: a prospective, randomized, double-blind, vehicle-controlled trial in adult Japanese patients." Clin Ther 27(6): 740-745.	4:	Did not report morphine consumption or 6 or 24 h pain scores
Garcia, J. B., et al. (2009). "Analgesic efficacy of intra-articular morphine high dose in patients submitted to total knee arthroplasty." European Journal of Pain 13: S192.	1:	Conference abstract
Gatti, A., et al. (2002). "Technique of postoperative anesthesia and analgesia in knee surgery. [Italian]." Impegno Ospedaliero, Sezione Scientifica 23(6): 47-51.	2:	Only anterior cruciate ligament reconstruction
Gimbel, J. and H. Ahdieh (2004). "The efficacy and safety of oral immediate-release oxymorphone for postsurgical pain." Anesth Analg 99(5): 1472-1477; table of contents.	2:	Included THA

Excluded studies and reason for exclusion: **1**: Not full reported trials, **2**: Included other surgery than TKA, **3**: Partial or bilateral TKA, revision TKA or fracture surgery, **4**: no relevant pain related outcome reported, **5**: Not controlled or randomized, **6**: Other reasons	
Gómez-Muñoz, C., et al. (1997) Effect of the '3 in 1' block pre- or post-operatively in postoperative pain following total knee arthroplasty. Revista Española de Anestesiología y Reanimación 44, 208-209	1: Conference abstract
Gong, L., et al. (2013). "Effects of combined application of muscle relaxants and celecoxib administration after total knee arthroplasty (TKA) on early recovery: a randomized, double-blind, controlled study." J Arthroplasty 28(8): 1301-1305.	4: Did not report morphine consumption or 6 or 24 h pain scores
Goodchild, C. S., et al. (2001). "Antinociceptive properties of neurosteroids IV: pilot study demonstrating the analgesic effects of alphadolone administered orally to humans." Br J Anaesth 86(4): 528-534.	6: Only 6 h postoperative study
Goyal, N., et al. (2013) Intra-articular infusion with bupivacaine decreased pain and opioid consumption after total knee arthroplasty. Journal of bone and joint surgery. American volume 95, 940 DOI: http://dx.doi.org/10.2106/JBJS.9510.ebo195	1: Summary clinical trial
Grevstad, U., et al. (2014). "Effect of Adductor Canal Block Versus Femoral Nerve Block on Quadriceps Strength, Mobilization, and Pain After Total Knee Arthroplasty: A Randomized, Blinded Study." Reg Anesth Pain Med.	6: Included patients on 1st or 2nd postoperative day if VAS greater than 60 mm
Grider, J. S., et al. (2011). "Comparison of single shot versus continuous femoral local anesthetic nerve block in patients undergoing knee arthroplasty." Regional Anesthesia and Pain Medicine 36 (5).	1: Conference abstract (see Goytizolo 2011)
Guan, D. w., et al. (2012). "Analgesic effect and security of tramadol/acetaminophen during perioperative total knee arthroplasty." Chinese Journal of Tissue Engineering Research 16(26): 4781-4785.	5: Uncontrollable postoperative regimen
Han, C. D., et al. (2007). "Intra-synovial ropivacaine and morphine for pain relief after total knee arthroplasty: a prospective, randomized, double blind study." Yonsei Med J 48(2): 295-300.	5: Uncontrollable postoperative regimen
Hanson, N. A., et al. (2014). "Continuous ultrasound-guided adductor canal block for total knee arthroplasty: a randomized, double-blind trial." Anesth Analg 118(6): 1370-1377.	5: Uncontrollable postoperative regimen
Hartrick, C. T., et al. (2006). "Evaluation of a single-dose, extended-release epidural morphine formulation for pain after knee arthroplasty." J Bone Joint Surg Am 88(2): 273-281.	6: Postoperative regimens were different: the study group received hydromorphone whereas the placebo group received morphine.
Hartrick, C. T., et al. (2011). "Capsaicin instillation for postoperative pain following total knee arthroplasty: a preliminary report of a randomized, double-blind, parallel-group, placebo-controlled, multicentre trial." Clin Drug Investig 31(12): 877-882.	6: Capsacin is not a medical intervention. Too few patients included for analyses (seven in each group). Preliminary study.
Hadzic, A., et al. (2016). "Liposome Bupivacaine Femoral Nerve Block for Postsurgical Analgesia after Total Knee Arthroplasty." Anesthesiology 124(6): 1372-1383.	4: No relevant primary endpoints
Himmelseher, S., et al. (2001). "Small-dose S(+)-ketamine reduces postoperative pain when applied with ropivacaine in epidural anesthesia for total knee arthroplasty." Anesth Analg 92(5): 1290-1295.	5: Uncontrollable postoperative regimen
Hinarejos, P., et al. (2016). "Local infiltration analgesia adds no clinical benefit in pain control to peripheral nerve blocks after total knee arthroplasty." Knee Surg Sports Traumatol Arthrosc.	6: Uncontrollable basic regimen and postoperative opioids
Hindle, E. and B. Shippey (2011). "Fascia iliaca compartment blockade plus intrathecal opiate improves six hour pain scores following primary total knee arthroplasty compared to intrathecal opiate alone." Regional Anesthesia and Pain Medicine 2): E256.	1: Conference abstract (see Dereli N 2011).
Hing, C. B. and J. B. Stiehl (2013). "Pain control following total knee replacement surgery." Knee 20(5): 299.	1: Editorial.
Ho, K. Y., et al. (2010). "Efficacy of duloxetine in reducing postoperative pain and opioid consumption after knee replacement surgery." Anaesthesia and Intensive Care 38 (3): 582-583.	1: Same data as Ho 2010 "Efficacy of duloxetine in reducing postoperative pain and opioid consumption after knee replacement surgery"
Holtman, A. P. (2008). "Postoperative pain relief after total-knee replacement: Peripheral nerve block analgesia just as effective as epidural analgesia. [Dutch]." Nederlands Tijdschrift voor Geneeskunde 152(32): 1797.	1: Letter
Huang, Y. S., et al. (2007). "Epidural clonidine for postoperative pain after total knee arthroplasty: a dose-response study." Anesth Analg 104(5): 1230-1235, tables of contents.	6: Study medication used as PCA as well. Not controllable regimen
Ikeuchi, M., et al. (2014). "Effects of dexamethasone on local infiltration analgesia in total knee arthroplasty: a randomized controlled trial." Knee Surg Sports Traumatol Arthrosc 22(7): 1638-1643.	5: Uncontrollable postoperative regimen
Ilfeld, B. M., et al. (2008). "Ambulatory continuous femoral nerve blocks decrease time to discharge readiness after tricompartment total knee arthroplasty: a randomized, triple-masked, placebo-controlled study." Anesthesiology 108(4): 703-713.	6: Randomization at postoperative day 1

Excluded studies and reason for exclusion: **1**: Not full reported trials, **2**: Included other surgery than TKA, **3**: Partial or bilateral TKA, revision TKA or fracture surgery, **4**: no relevant pain related outcome reported, **5**: Not controlled or randomized, **6**: Other reasons	
Ilfeld, B. M., et al. (2010). "A multicenter, randomized, triple-masked, placebo-controlled trial of the effect of ambulatory continuous femoral nerve blocks on discharge-readiness following total knee arthroplasty in patients on general orthopaedic wards." Pain 150(3): 477-484.	6: Randomization at postoperative day 1
Jaeger, P., et al. (2012). "Effect of adductor-canal-blockade on established, severe post-operative pain after total knee arthroplasty: a randomised study." Acta Anaesthesiol Scand 56(8): 1013-1019.	6: Only 0-6 hour postoperative study
Jaeger, P., et al. (2014). "Adductor canal block for postoperative pain treatment after revision knee arthroplasty: a blinded, randomized, placebo-controlled study." PLoS One 9(11): e111951.	2: Included only revision TKA
Jagla, C., et al. (2014). "Peripheral opioid receptor blockade increases postoperative morphine demands-A randomized, double-blind, placebo-controlled trial." Pain 155(10): 2056-2062.	6: The study investigated whether periferal opioid-receptor blocade increased postoperative morphine consumption. It is a pain increasing study
Jain, A., et al. (2012). "Analgesic efficacy of low-dose intrathecal neostigmine in combination with fentanyl and bupivacaine for total knee replacement surgery." J Anaesthesiol Clin Pharmacol 28(4): 486-490.	4: Did not report morphine consumption or 6 or 24 h pain scores
Jain, P., et al. (2012). "Evaluation of efficacy of oral pregabalin in reducing postoperative pain in patients undergoing total knee arthroplasty." Indian J Orthop 46(6): 646-652.	6: Two different kinds of rescue medication. Only average pain scores were reported. Primary end points were not assessable
Jianda, X., et al. (2016). "Impact of Preemptive Analgesia on inflammatory responses and Rehabilitation after Primary Total Knee Arthroplasty: A Controlled Clinical Study." Sci Rep 6: 30354.	6: Multimodal analgesic protocol. Too many different drugs to control the intervention
Kadic, L., et al. (2009). "Continuous femoral nerve block after total knee arthroplasty?" Acta Anaesthesiol Scand 53(7): 914-920.	4: Did not report morphine consumption or 6 or 24 h pain scores
Kadic, L., et al. (2012). "The effect of addition of pregabalin and s-ketamine to local infiltration analgesia on the knee function outcome after total knee arthroplasty." Acta Anaesthesiol Belg 63(3): 111-114.	5: Not controlled
Kampe, S., et al. (2003). "Continuous epidural infusion of ropivacaine with sufentanil 1.5 microg x mL(-1) for postoperative analgesia after total knee replacement." Can J Anaesth 50(6): 617-618.	1: Letter to editor
Kandikatu, S., et al. (2006) Acute post operative pain management in total knee arthroplasty; a comparative study between PCA and PCA with local nerve blocks. The Journal of Bone and Joint Surgery (Proceedings) 88-b, 255-225a	1: Abstract
Kelley, T. C., et al. (2013). "Efficacy of multimodal perioperative analgesia protocol with periarticular medication injection in total knee arthroplasty: a randomized, double-blinded study." J Arthroplasty 28(8): 1274-1277.	5: Uncontrollable postoperative regimen
Klasen, J. A., et al. (1999). "Intraarticular, epidural, and intravenous analgesia after total knee arthroplasty." Acta Anaesthesiol Scand 43(10): 1021-1026.	5: Not controlled. Open label study
Koh, I. J., et al. (2013). "Preemptive low-dose dexamethasone reduces postoperative emesis and pain after TKA: a randomized controlled study." Clin Orthop Relat Res 471(9): 3010-3020.	5: Uncontrollable postoperative regimen
Koh, I. J., et al. (2012). "Does periarticular injection have additional pain relieving effects during contemporary multimodal pain control protocols for TKA?: A randomised, controlled study." Knee 19(4): 253-259.	5: Uncontrollable postoperative regimen
Krenzel, B. A., et al. (2009). "Posterior capsular injections of ropivacaine during total knee arthroplasty: a randomized, double-blind, placebo-controlled study." J Arthroplasty 24(6 Suppl): 138-143.	5: Uncontrollable postoperative regimen
Kuhne, J., et al. (2005). "Perioperative analgesia for knee arthroplasty [2]." British Journal of Anaesthesia 94(3): 393-395.	1: Letter to editor
Kwon, S. K., et al. (2014). "Periarticular injection with corticosteroid has an additional pain management effect in total knee arthroplasty." Yonsei Med J 55(2): 493-498.	3: Follow up and bilateral TKA
Laffey, J. G. and N. Flynn (2001). "Low-dose spinal morphine for postoperative analgesia following knee arthroplasty." Br J Anaesth 86(1): 152-153.	1: Letter
Lang, S. A. (1999). "Postoperative analgesia following total knee arthroplasty: a study comparing spinal anesthesia and combined sciatic femoral 3-in-1 block." Reg Anesth Pain Med 24(1): 97.	1: Letter to the editor
Lau, H. P., et al. (1998). "Regional nerve block for total knee arthroplasty." J Formos Med Assoc 97(6): 428-430.	5: Not randomized
Lauretti, G. R., et al. (2000). "Postoperative analgesia by intraarticular and epidural neostigmine following knee surgery." J Clin Anesth 12(6): 444-448.	2: TKA and arthroscopy/meniscus repair
Lauretti, G. R., et al. (1999). "Study of three different doses of epidural neostigmine coadministered with lidocaine for postoperative analgesia." Anesthesiology 90(6): 1534-1538.	2: TKA and arthroscopy/meniscus repair

Excluded studies and reason for exclusion: **1**: Not full reported trials, **2**: Included other surgery than TKA, **3**: Partial or bilateral TKA, revision TKA or fracture surgery, **4**: no relevant pain related outcome reported, **5**: Not controlled or randomized, **6**: Other reasons	
Lauretti, G. R., et al. (1999). "Transdermal nitroglycerine enhances spinal sufentanil postoperative analgesia following orthopedic surgery." Anesthesiology 90(3): 734-739.	2: Arthroscopy/meniscus repair
Lauretti, G. R., et al. (2001). "The effect of transdermal nitroglycerin on spinal S(+)-ketamine antinociception following orthopedic surgery." J Clin Anesth 13(8): 576-581.	2: Other surgery than TKA
Lauretti, G. R., et al. (2005). "The combination of epidural clonidine and S(+)-ketamine did not enhance analgesic efficacy beyond that for each individual drug in adult orthopedic surgery." J Clin Anesth 17(2): 79-84.	2: TKA and arthroscopy/meniscus repair
Lee, J. J., et al. (2012). "Effect of continuous psoas compartment block and intravenous patient controlled analgesia on postoperative pain control after total knee arthroplasty." Korean J Anesthesiol 62(1): 47-51.	5: Two different interventions
Lombardi, A. V., Jr., et al. (2004). "Soft tissue and intra-articular injection of bupivacaine, epinephrine, and morphine has a beneficial effect after total knee arthroplasty." Clin Orthop Relat Res(428): 125-130.	6: Retrospective review
Long, W. T., et al. (2006). "Postoperative pain management following total knee arthroplasty: a randomized comparison of continuous epidural versus femoral nerve infusion." J Knee Surg 19(2): 137-143.	5: Two different interventions: Continous epidural catheter vs. Femoral nerve catheter
Lorenzini, C., et al. (2002). "Efficacy of ropivacaine compared with ropivacaine plus sufentanil for postoperative analgesia after major knee surgery." Anaesthesia 57(5): 424-428.	2: TKA and cruciate ligament reconstruction
Lunn, T. H., et al. (2010). "Effect of preoperative methylprednisolone on pain and recovery after total knee arthroplasty: A randomized, double-blind, placebo-controlled trial." Regional Anesthesia and Pain Medicine 35 (5): E45.	1: Conference abstract
Macalou, D., et al. (2004). "Postoperative analgesia after total knee replacement: the effect of an obturator nerve block added to the femoral 3-in-1 nerve block." Anesth Analg 99(1): 251-254.	5: Uncontrollable postoperative regimen
Madei, W., et al. (2001). "Reducing post-operative analgesic requirements by pre-medication with dextromethorphan. [German]." Anasthesiologie und Intensivmedizin 42(12): 942-945.	2: Only cruciate ligament reconstruction
Man, Z. T., et al. (2010). "Local injection of bupivacaine following total knee arthroplasty: Analgesia effect and safety evaluation. [Chinese]." Journal of Clinical Rehabilitative Tissue Engineering Research 14(26): 4774-4777.	4: Included bilateral TKA and the study was only 0-12 h postoperative
Mangar, D., et al. (2014). "Knee strength retention and analgesia with continuous perineural fentanyl infusion after total knee replacement: randomized controlled trial." J Anesth 28(2): 214-221.	5: Not properly controlled. Different PCA in groups
Mejia-Terrazas, G. E., et al. (2007). "Postoperative analgesia for total knee arthroplasty; a comparative study. [Spanish]." Revista Mexicana de Anestesiologia 30(4): 197-200.	5: Three different interventions. PCA vs sciatic block vs epidural catheder
Meunier, A., et al. (2007). "Effects of celecoxib on blood loss, pain, and recovery of function after total knee replacement: a randomized placebo-controlled trial." Acta Orthop 78(5): 661-667.	4: Did not report morphine consumption or 6 or 24 h pain scores
Militello, M. G., et al. (2010). "Continuous femoral nerve block using levobupivacaine 0.18%: Effects on the rehabilitation following total knee arthroplasty with subvastus approach." Regional Anesthesia and Pain Medicine 35 (5).	1: Conference abstract
Minkowitz, H., et al. (2010). "A phase 2 multicenter, randomized, placebo-controlled study to evaluate the clinical efficacy, safety, and tolerability of sublingual sufentanil nanotaba, in patients following elective unilateral knee replacement surgery." Regional Anesthesia and Pain Medicine 35 (5).	1: Conference abstract
Mollov, J., et al. (2012). "The effect of a peri-articular injection mixture of ropivacaine, epinephrine, ketorolac, and morphine in addition to an established multimodal perioperative analgesic regimen for total knee arthroplasty, on postoperative pain and rehabilitation." Regional Anesthesia and Pain Medicine 37 (6).	1: Conference abstract
Montazeri, K., et al. (2007) Pre-emptive gabapentin significantly reduces postoperative pain and morphine demand following lower extremity orthopaedic surgery. Singapore medical journal 48, 748-751	2: Knee arthroscopy
Morin, A. M., et al. (2005). "Postoperative analgesia and functional recovery after total-knee replacement: comparison of a continuous posterior lumbar plexus (psoas compartment) block, a continuous femoral nerve block, and the combination of a continuous femoral and sciatic nerve block." Reg Anesth Pain Med 30(5): 434-445.	5: Uncontrollable postoperative regimen
Motamed, C., et al. (2000). "Preemptive intravenous morphine-6-glucuronide is ineffective for postoperative pain relief." Anesthesiology 92(2): 355-360.	2: Open knee surgery: total knee prosthesis, tibia osteotomy or ligamentoplasty

Excluded studies and reason for exclusion: **1**: Not full reported trials, **2**: Included other surgery than TKA, **3**: Partial or bilateral TKA, revision TKA or fracture surgery, **4**: no relevant pain related outcome reported, **5**: Not controlled or randomized, **6**: Other reasons	
Moustafa, M. A. and R. S. Saleh (2012). "Nalbuphine added to intrathecal morphine in total knee arthroplasty; effect on postoperative analgesic requirements and morphine related side effects." Alexandria Journal of Medicine 48(2): 175-178.	4: Did not report morphine consumption or 6 or 24 h pain scores
Mulford, J. S., et al. (2015). "Short-term outcomes of local infiltration anaesthetic in total knee arthroplasty: a randomized controlled double-blinded controlled trial." ANZ J Surg.	4: Did not report morphine consumption or 6 or 24 h pain scores
Munteanu, A. M., et al. (2016). "Is there any analgesic benefit from preoperative vs. postoperative administration of etoricoxib in total knee arthroplasty under spinal anaesthesia?: A randomised double-blind placebo-controlled trial." Eur J Anaesthesiol.	6: Differences in initiation of basic analgesic regimen
Nafissi, A., et al. (2011). "Lidoderm effectiveness in reducing pain in post-operative unilateral knee replacements patients." Journal of Pain 1): P53.	1: Conference abstract
Nechleba, J., et al. (2005). "Continuous intra-articular infusion of bupivacaine for postoperative pain following total knee arthroplasty." J Knee Surg 18(3): 197-202.	6: Differences in basic analgesic regimen between groups. No relevant endpoints
Nielsen, B. S. and I. Kjeldal (1995) Intra-articular morphine in knee arthroplasty for postoperative analgesia; a randomized study. Acta Anaesthesiol Scand 39, 147.	1: Conference abstract
Niruthisard, S., et al. (2011). "Comparison of analgesic efficacy among pregabalin, celecoxib, pregabalin with celecoxib and placebo after total knee arthroplasty under intrathecal morphine." Regional Anesthesia and Pain Medicine 2): E273.	1: Conference abstract
Omais, M., et al. (2002). "Epidural morphine and neostigmine for postoperative analgesia after orthopedic surgery." Anesth Analg 95(6): 1698-1701, table of contents.	2: Included knee arthroscopy and meniscus repair
Orbach-Zinger, S., et al. (2009). "Transdermal nitroglycerin as an adjuvant to patient-controlled morphine analgesia after total knee arthroplasty." Pain Res Manag 14(2): 109-112.	6: Study stopped prematurely due to two myocardial infarcts in the study group
Osawa, A., et al. (2014). "Efficacy of periarticular multimodal drug injection in total knee arthroplasty for pain management and rehabilitation." Osteoarthritis and Cartilage 22: S415-S416.	6: Differences in basic analgesic regimen between groups
Park, C. K. and C. K. Cho (2010). "Effect of ketorolac administered by a continuous femoral infusion with 0.125% bupivacaine after total knee replacement." Regional Anesthesia and Pain Medicine 35 (5): E154.	1: Conference abstract
Perrin, S. B. and A. N. Purcell (2009). "Intraoperative ketamine may influence persistent pain following knee arthroplasty under combined general and spinal anaesthesia: a pilot study." Anaesth Intensive Care 37(2): 248-253.	6: Differences in basic analgesic regimen between groups
Pham Dang, C., et al. (2005). "The value of adding sciatic block to continuous femoral block for analgesia after total knee replacement." Reg Anesth Pain Med 30(2): 128-133.	5: Uncontrollable postoperative regimen; number of femoral nerve block boluses not registered
Rahimi, M., et al. (2011). "Effect of additioning of triamcinolon to cocktail of fentanil epinephrinated bupivacaine in periarticular injection for post operative pain and quality improvement after total knee arthroplasty." Journal of Isfahan Medical School 29(144).	5: Not properly controlled
Rais, K., et al. (2009). "Multimodal regional and systemic approach for postoperative analgesia in total knee arthroplasty." European Journal of Anaesthesiology 26: 123.	1: Conference abstract
Raj, P. P., et al. (1987). "Comparison of continuous epidural infusion of a local anesthetic and administration of systemic narcotics in the management of pain after total knee replacement surgery." Anesth Analg 66(5): 401-406.	5: Not controlled. Systemic analgesics vs. Continous epidural
Rajeev, S., et al. (2007). "Combined continuous "3-in-1" and sciatic nerve blocks provide improved postoperative analgesia with no correlation to catheter tip location after unilateral total knee arthroplasty." J Arthroplasty 22(8): 1181-1186.	5: Uncontrollable postoperative regimen with differences amongst groups
Ramamoorthy, K. G. (2012). "Local infiltration analgesia following total knee arthroplasty." Indian J Anaesth 56(2): 208-209.	1: Letter
Rasmussen, G. L., et al. (2002). "Intravenous parecoxib sodium foracute pain after orthopedic knee surgery." Am J Orthop (Belle Mead NJ) 31(6): 336-343.	6: Did only include patients with postoperative VAS > 45mm and did not initiate the intervention before discontinuation of PCA
Rauf, J., et al. (2013). "Does saphenous nerve block improve analgesia after total knee replacement when used in combination with local infiltration analgesia a prospective randomised double blinded controlled trial." Regional Anesthesia and Pain Medicine 1): E142.	1: Conference abstract
Reay, B. A., et al. (1989) Low-dose intrathecal diamorphine analgesia following major orthopaedic surgery. British Journal of Anaesthesia 62, 248-252	2: Included TKA
Reeves, M. and M. W. Skinner (2009). "Continuous intra-articular infusion of ropivacaine after unilateral total knee arthroplasty." Anaesth Intensive Care 37(6): 918-922.	5: Uncontrollable postoperative regimen with differences amongst groups

Excluded studies and reason for exclusion: **1**: Not full reported trials, **2**: Included other surgery than TKA, **3**: Partial or bilateral TKA, revision TKA or fracture surgery, **4**: no relevant pain related outcome reported, **5**: Not controlled or randomized, **6**: Other reasons	
Reuben, S. S., et al. (2008). "A prospective randomized trial on the role of perioperative celecoxib administration for total knee arthroplasty: improving clinical outcomes." Anesth Analg 106(4): 1258-1264, table of contents.	6: Retracted study
Reuben, S. S., et al. (2002). "Evaluation of the safety and efficacy of the perioperative administration of rofecoxib for total knee arthroplasty." J Arthroplasty 17(1): 26-31.	6: Retracted study
Reynolds, L. W., et al. (2003). "The COX-2 specific inhibitor, valdecoxib, is an effective, opioid-sparing analgesic in patients undergoing total knee arthroplasty." J Pain Symptom Manage 25(2): 133-141.	3: Included revision TKA
Ritter, M. A., et al. (1999). "Intra-articular morphine and/or bupivacaine after total knee replacement." J Bone Joint Surg Br 81(2): 301-303.	6: Differences in basic analgesic regimen between groups
Romberg, R., et al. (2007). "A randomized, double-blind, placebo-controlled pilot study of IV morphine-6-glucuronide for postoperative pain relief after knee replacement surgery." Clin J Pain 23(3): 197-203.	6: Tests the effect of morphine-6-glucuronide on postoperative morphine consumption. The intervention and PCA is practically the same
Safa, B., et al. (2011). "A prospective, randomized trial, comparing analgesic efficacy and postoperative functional recovery of either single shot sciatic nerve block or posterior capsule infiltration combined with femoral block for total knee arthroplasty." Regional Anesthesia and Pain Medicine 36 (5).	1: Conference abstract
Sanders, J. C., et al. (2009). "Intrathecal baclofen for postoperative analgesia after total knee arthroplasty." J Clin Anesth 21(7): 486-492.	1: Included revision TKA
Santiveri Papiol, X., et al. (2009). "[Epidural analgesia versus femoral or femoral-sciatic nerve block after total knee replacement: comparison of efficacy and safety]." Rev Esp Anestesiol Reanim 56(1): 16-20.	5: Observational study
Sarridou, D. G., et al. (2016). "Parecoxib Possesses Anxiolytic Properties in Patients Undergoing Total Knee Arthroplasty: A Prospective, Randomized, Double-Blind, Placebo-Controlled, Clinical Study." Pain Ther 5(1): 55-62.	6: Same data as Sarridou, D. G., et al. (2015). "Intravenous parecoxib and continuous femoral block for postoperative analgesia after total knee arthroplasty. A randomized, double-blind, prospective trial." Pain Physician 18(3): 267-276.
Savage, P., et al. (2009). "Efficacy and safety of a single intraoperative administration of highly purified capsaicin formulation for management of postoperative pain associated with total knee arthroplasty: A randomized phase 3 trial." Journal of Pain 1): S53.	1: Conference abstract
Sawhney, M., et al. (2016). "Pain After Unilateral Total Knee Arthroplasty: A Prospective Randomized Controlled Trial Examining the Analgesic Effectiveness of a Combined Adductor Canal Peripheral Nerve Block with Periarticular Infiltration Versus Adductor Canal Nerve Block Alone Versus Periarticular Infiltration Alone." Anesth Analg 122(6): 2040-2046.	6: Different rescues analgesic, not controllable
Sculco, T. P. (2012). "Local infiltration analgesia reduced postoperative morphine consumption and provided better pain relief than intrathecal morphine after total knee arthroplasty." J Bone Joint Surg Am 94(16): 1511-1513.	5: Compares local infiltration analgesia against intrathecal morphine
Serrano, A., et al. (2011). "Analgesic efficacy of associating a sciatic block to a femoral block in the posotperative period of total knee arthroplasty." European Journal of Anaesthesiology 28: 119-120.	1: Abstract
Sharma, B., et al. (2016). "Effect of addition of dexmedetomidine to ropivacaine 0.2% for femoral nerve block in patients undergoing unilateral total knee replacement: A randomised double-blind study." Indian J Anaesth 60(6): 403-408.	6: Different rescues analgesic, not controllable
Shen, B., et al. (2009). "[Effects of perioperative administration of celecoxib on pain management and recovery of function after total knee replacement]." Zhonghua Wai Ke Za Zhi 47(2): 116-119.	5: Administers the intervention: celecoxib to both to active and control group. Not controlled
Sherif, A. A., et al. (2014). "Evaluation of the feasibility of dexamesthasone adde to bupivacaine for continuous femoral nerve block for postoperative analgesia and rehabilitation after reconstructive knee surgery." Anesth Analg 1): S275.	1: Conference abstract
Sherif, A. A. and H. E. Elsersy (2016). "Dexamethasone as adjuvant for femoral nerve block following knee arthroplasty: a randomized, controlled study." Acta Anaesthesiol Scand 60(7): 977-987.	6: Different rescues analgesic, not controllable
Shum, C. F., et al. (2009). "Continuous femoral nerve block in total knee arthroplasty: immediate and two-year outcomes." J Arthroplasty 24(2): 204-209.	6: Reported same data as Seet et al. 2006
Sinatra, R. S., et al. (2005). "Efficacy and safety of single and repeated administration of 1 gram intravenous acetaminophen injection (paracetamol) for pain management after major orthopedic surgery." Anesthesiology 102(4): 822-831.	2: Included THA
Singelyn, F. J., et al. (1998). "Effects of intravenous patient-controlled analgesia with morphine, continuous epidural analgesia, and continuous three-in-one block on postoperative pain and knee rehabilitation after unilateral total knee arthroplasty." Anesth Analg 87(1): 88-92.	5: Three different interventions: PCA morphine vs. 3-1 block vs. Continuous epidural analgesia

Excluded studies and reason for exclusion: **1**: Not full reported trials, **2**: Included other surgery than TKA, **3**: Partial or bilateral TKA, revision TKA or fracture surgery, **4**: no relevant pain related outcome reported, **5**: Not controlled or randomized, **6**: Other reasons	
Sites, B. D., et al. (2003). "Intrathecal clonidine added to a bupivacaine-morphine spinal anesthetic improves postoperative analgesia for total knee arthroplasty." Anesth Analg 96(4): 1083-1088, table of contents.	3: Includes bilateral TKA
Smith, T. W., et al. (2009). "Efficacy and safety of morphine-6-glucuronide (M6G) for postoperative pain relief: a randomized, double-blind study." Eur J Pain 13(3): 293-299.	6: Tests the effect of morphine-6-glucuronide on postoperative morphine consumption. The intervention and PCA is practically the same
Sobrinho, H. G., et al. (2011). "Analgesic efficacy of the intra-articular administration of s(+)-ketamine in patients undergoing total knee arthroplasty." European Journal of Pain Supplements 5 (1): 270.	6: Same data as Guara sobrinho
Soto Mesa, D., et al. (2012). "[Control of postoperative pain in knee arthroplasty: single dose femoral nerve block versus continuous femoral block]." Rev Esp Anestesiol Reanim 59(4): 204-209.	5: Different catheters for each group. No recordable analgesic consumption.
Strebel, S., et al. (2004). "Small-dose intrathecal clonidine and isobaric bupivacaine for orthopedic surgery: a dose-response study." Anesth Analg 99(4): 1231-1238, table of contents.	2: Included THA
Sundarathiti, P., et al. (2012). "Comparison of continuous femoral nerve block (CFNB/SA) and continuous femoral nerve block with mini-dose spinal morphine (CFNB/SAMO) for postoperative analgesia after Total Knee Arthroplasty (TKA)." Regional Anesthesia and Pain Medicine 1): E233.	1: Conference abstract
Szczukowski, M. J., Jr., et al. (2004). "Femoral nerve block for total knee arthroplasty patients: a method to control postoperative pain." J Arthroplasty 19(6): 720-725.	6: The corresponding author was contacted about endpoints. He did not want the study included as he did not have further data
Vercauteren, M. P., et al. (1998). "Postoperative intrathecal patient-controlled analgesia with bupivacaine, sufentanil or a mixture of both." Anaesthesia 53(10): 1022-1027.	2: Included THA
Wadhwa, A., et al. (2001). "Large-dose oral dextromethorphan as an adjunct to patient-controlled analgesia with morphine after knee surgery." Anesth Analg 92(2): 448-454.	2: Included THA
Weber, A., et al. (2001). "Epinephrine does not prolong the analgesia of 20 mL ropivacaine 0.5% or 0.2% in a femoral three-in-one block." Anesth Analg 93(5): 1327-1331.	6: Differences in basic analgesic regimen between groups.
Wegener, J., et al. (2010). "Long-term effects of sciatic nerve block in addition to femoral nerve block on functional outcome after total knee arthroplasty (TKA)." Regional Anesthesia and Pain Medicine 35 (5): E190-E191.	1: Conference abstract
Wegener, J. T., et al. (2011). "Value of single-injection or continuous sciatic nerve block in addition to a continuous femoral nerve block in patients undergoing total knee arthroplasty: a prospective, randomized, controlled trial." Reg Anesth Pain Med 36(5): 481-488.	6: Differences in basic analgesic regimen between groups
Weir, P. S. and J. P. Fee (1998). "Double-blind comparison of extradural block with three bupivacaine-ketamine mixtures in knee arthroplasty." Br J Anaesth 80(3): 299-301.	4: When the patients reported a VAS above 30 mm registrations stopped. No further registrations were assessed after this point
Wolke, B., et al. (2000) [The influence of peri- and post-operative peridural anaesthesia and analgesia on the result of early rehabilitation after endo-prosthetic knee replacement in RA.]. Aktuelle Rheumatologie 25, 170	1: Conference abstract
Wu, J. W. and Y. C. Wong (2014). "Elective unilateral total knee replacement using continuous femoral nerve blockade versus conventional patient-controlled analgesia: perioperative patient management based on a multidisciplinary pathway." Hong Kong Med J 20(1): 45-51.	5: Two different interventions: Continous FNB vs. PCA morphine
Xie, Z., et al. (2012). "Three-in-one nerve block with different concentrations of bupivacaine in total knee arthroplasty: randomized, placebo-controlled, double-blind trial." J Arthroplasty 27(5): 673-678.e671.	4: Did not report morphine consumption or 6 or 24 h pain scores
Yilmaz, R., et al. (2009). "Knee arthroplasty and femoral block. [Turkish]." Anestezi Dergisi 17(2): 78-85.	5: Not randomized. Different doses of levobupivacaine in the two groups
Yu, H. P., et al. (2010). "[Effect of continuous femoral nerve block in analgesia and the early rehabilitation after total knee replacement]." Zhongguo Gu Shang 23(11): 825-827.	5: Two different interventions
Zaric, D., et al. (2007). "Combined femoral-sciatic catheters for postoperative pain treatment after total knee replacement [24]." Anesth Analg 105(1): 288-289.	1: Letter to the editor
Znojek-Tymborowska, J., et al. (2013). "Relevance of infiltration analgesia in pain relief after total knee arthroplasty." Acta Ortop Bras 21(5): 262-265.	5: Not randomized
Zugliani, A. H., et al. (2007). "[Control of postoperative pain following total knee arthroplasty: is it necessary to associate sciatic nerve block to femoral nerve block?]." Rev Bras Anestesiol 57(5): 514-524.	4: Did not report morphine consumption or 6 or 24 h pain scores
