# Supplementary material for: Postoperative pain treatment after total knee arthroplasty: A systematic review
Source: PLoS One. 2017 Mar 8;12(3):e0173107. doi: 10.1371/journal.pone.0173107 (PMC5342240; doi:10.1371/journal.pone.0173107)
Supplement: S4 Appendix — (PDF) [file pone.0173107.s004.pdf]

Appendix 4: Trials reporting specific outcomes

VAS 0-10: [16-20, 28, 29, 35, 37, 40, 41, 45, 47, 49, 50, 54-56, 58-64, 67, 69, 70, 72, 73, 75-77, 80, 81, 86, 88, 92, 94, 98, 103, 106, 109, 110, 112, 114-117, 123, 125, 128]
VAS 0-100: [23, 25, 27, 30-34, 36, 38, 39, 43, 46, 48, 51-53, 65, 66, 68, 78, 79, 82, 85, 89, 91, 93, 96, 99-101, 104, 107, 108, 111, 113, 118, 120-122, 124, 127]
(V)NRS 0-10: [21, 22, 26, 42, 44, 57, 71, 84, 87, 95, 97, 102, 105, 119, 126]
WOMAC 0-10: [83]
Visual Pain Scale 0-3: [24, 74]

Pain score 6 hour at rest: [16-18, 21, 23-27, 31, 33, 35, 37-40, 42-44, 46, 47, 49-57, 59, 60, 63-65, 68-71, 73-75, 77-81, 84, 85, 89, 91-94, 96-100, 102-108, 110-127]
Pain score 24 hour at rest: [16-18, 21, 22, 24-28, 30, 31, 33, 35-40, 42-44, 46, 47, 49-56, 59, 60, 63-66, 68-71, 73-75, 77, 79-82, 84, 85, 87-89, 91-100, 102-113, 115-119, 121-127]
Pain score 6 hour at movement: [18, 23, 24, 26, 35, 37, 41, 42, 46, 48, 51, 53, 56, 60, 63, 64, 70, 81, 84, 85, 89, 92, 97-99, 101, 102, 111, 119, 121, 124, 126, 127]
Pain score 24 hour at movement: [16-18, 22, 24, 26, 28, 30, 35, 37, 41, 42, 45, 46, 48, 51-53, 56, 60, 63, 64, 70, 73, 79, 81, 83-85, 89, 92, 97-99, 101, 102, 111, 115, 121, 124, 126, 127]

Vomiting, nausea, or both: [16-19, 21-26, 29, 30, 32, 34-37, 39-47, 49-56, 58-60, 62, 64-67, 69, 72-76, 78, 80-92, 96-100, 102-106, 108, 110, 111, 113, 115-128]
Sedation: [16, 22, 34, 35, 37, 41, 42, 44, 46, 51, 72, 75, 81, 82, 86, 87, 105, 109, 110, 116, 117, 120, 121, 126]
Dizziness: [18, 21, 23, 26, 42, 47, 60, 62, 76, 78, 82, 84, 86, 106, 115, 126]
Pruritus: [16-18, 22, 26, 29, 34, 35, 39-47, 49-51, 55, 62, 64, 75, 76, 80-84, 86, 90, 98, 108, 110, 111, 113, 115, 117, 118, 125, 126, 128]

Length of stay: [17, 22, 24, 27, 28, 35, 37, 38, 40, 48, 51, 53, 55, 59-62, 70, 80, 85-88, 91, 95, 100, 102, 107-112, 121, 122, 127]
Predefined discharge criteria: [27, 28, 37, 38, 51, 53, 60, 61, 70, 85, 88, 102, 107, 111, 121]

Trials with:
Low assay sensitivity for pain scores: [21, 25, 42, 49, 58, 62, 72, 75, 77, 87, 91, 93, 96, 98, 101, 112, 113, 116, 123]
Low assay sensitivity for morphine consumption: [34, 49, 62, 67, 70, 77, 79, 91, 98, 100, 106, 114, 128]
A basic analgesic regimen: [16, 21, 22, 26, 32, 33, 35, 37-41, 46, 49-52, 54, 56-58, 60-64, 70, 71, 77, 79, 82-89, 91-93, 97-103, 107-115, 120, 122-128]
