# Supplementary figures and images for: Postoperative pain treatment after total knee arthroplasty: A systematic review
Source: PLoS One. 2017 Mar 8;12(3):e0173107. doi: 10.1371/journal.pone.0173107 (PMC5342240; doi:10.1371/journal.pone.0173107)

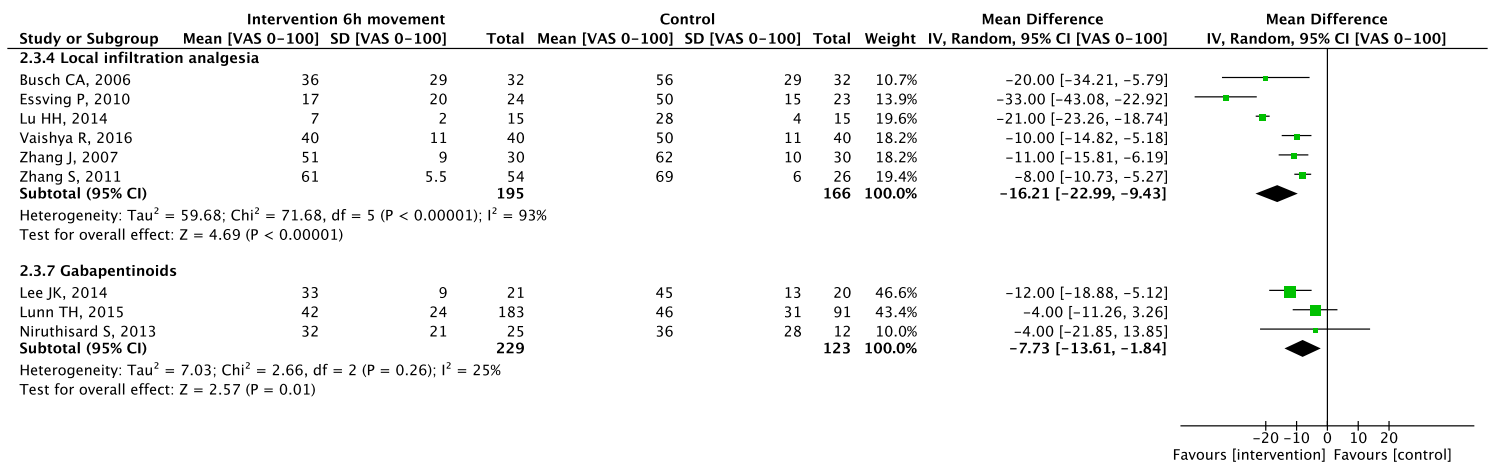

Supplement: S5 Appendix — Green squares with horizontal lines represent mean differences and 95% confidence intervals for each trial. Black tiles represent the mean difference of each intervention. (PDF) [file pone.0173107.s005.pdf]

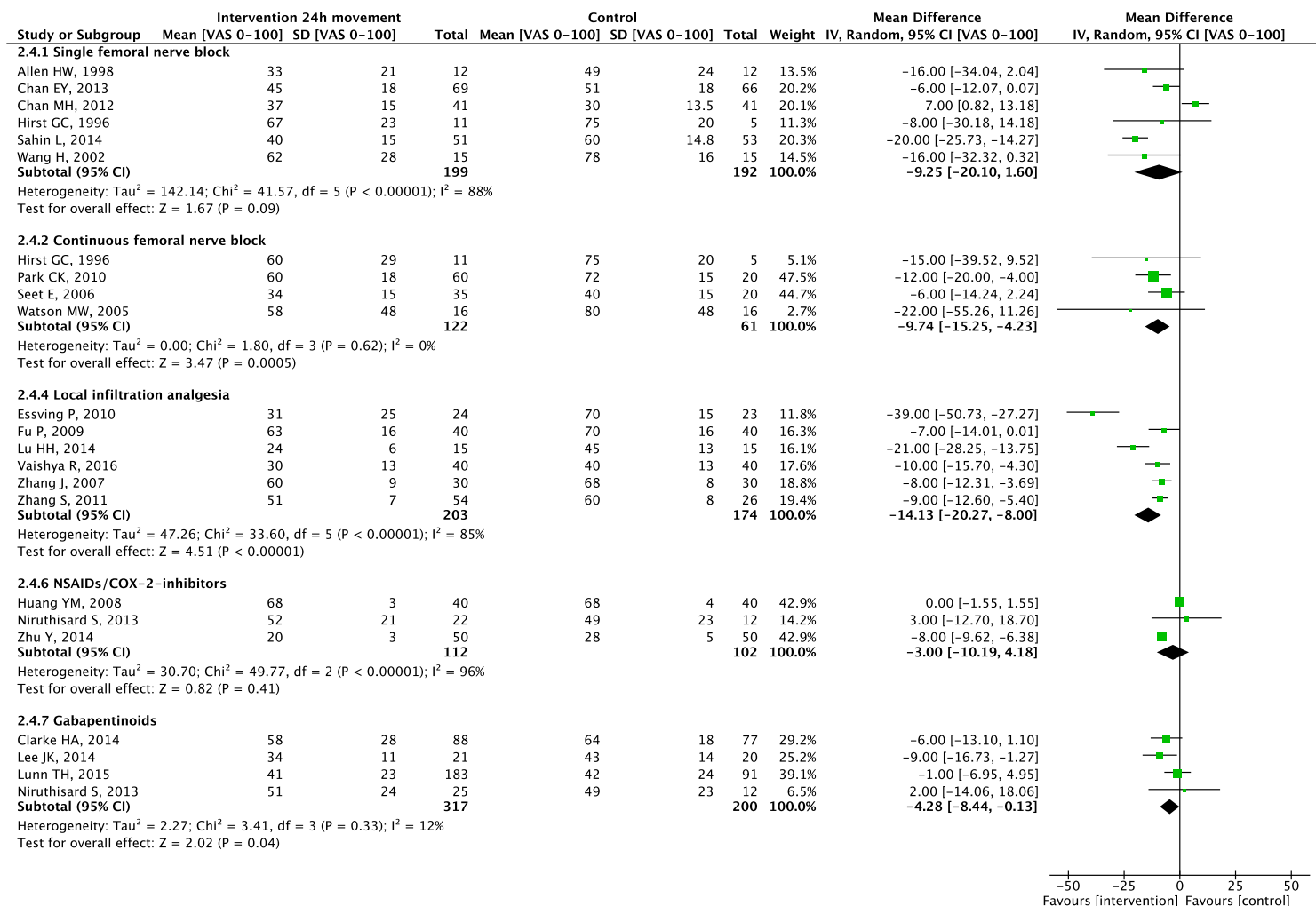

Supplement: S6 Appendix — Green squares with horizontal lines represent mean differences and 95% confidence intervals for each trial. Black tiles represent the mean difference of each intervention. (PDF) [file pone.0173107.s006.pdf]

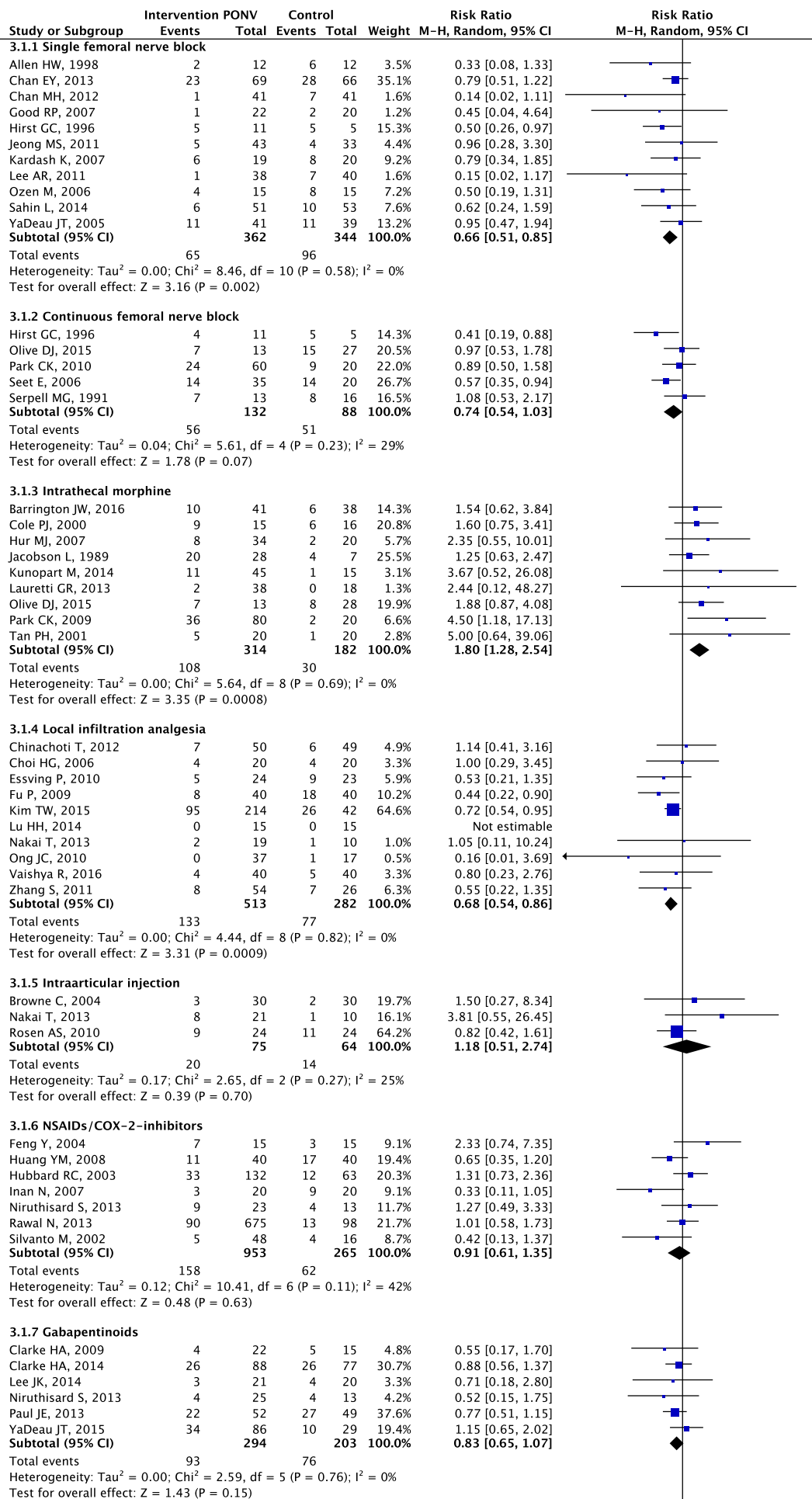

Supplement: S7 Appendix — Blue squares with horizontal lines represent mean differences and 95% confidence intervals for each trial. Black tiles represent the mean difference of each intervention. (PDF) [file pone.0173107.s007.pdf]

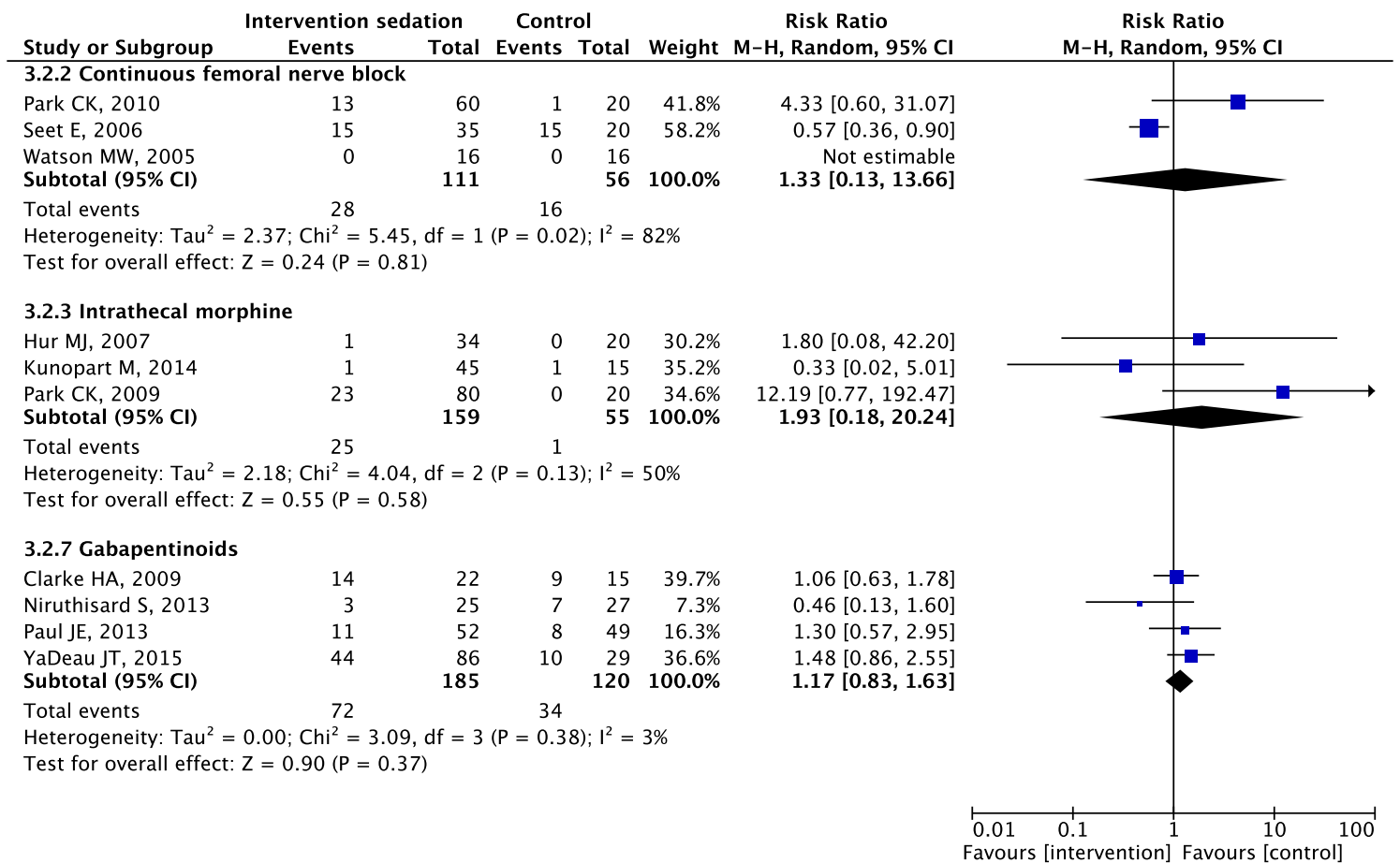

Supplement: S8 Appendix — Blue squares with horizontal lines represent mean differences and 95% confidence intervals for each trial. Black tiles represent the mean difference of each intervention. (PDF) [file pone.0173107.s008.pdf]

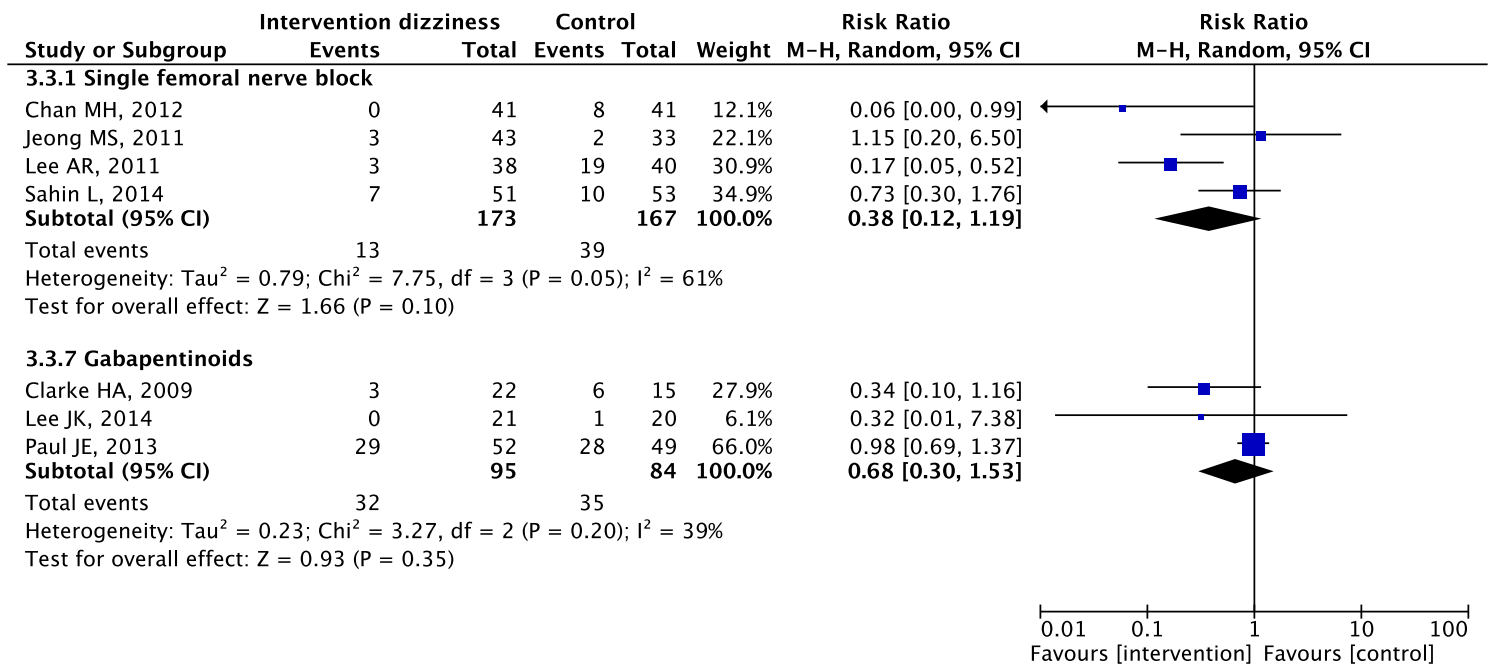

Supplement: S9 Appendix — Blue squares with horizontal lines represent mean differences and 95% confidence intervals for each trial. Black tiles represent the mean difference of each intervention. (PDF) [file pone.0173107.s009.pdf]

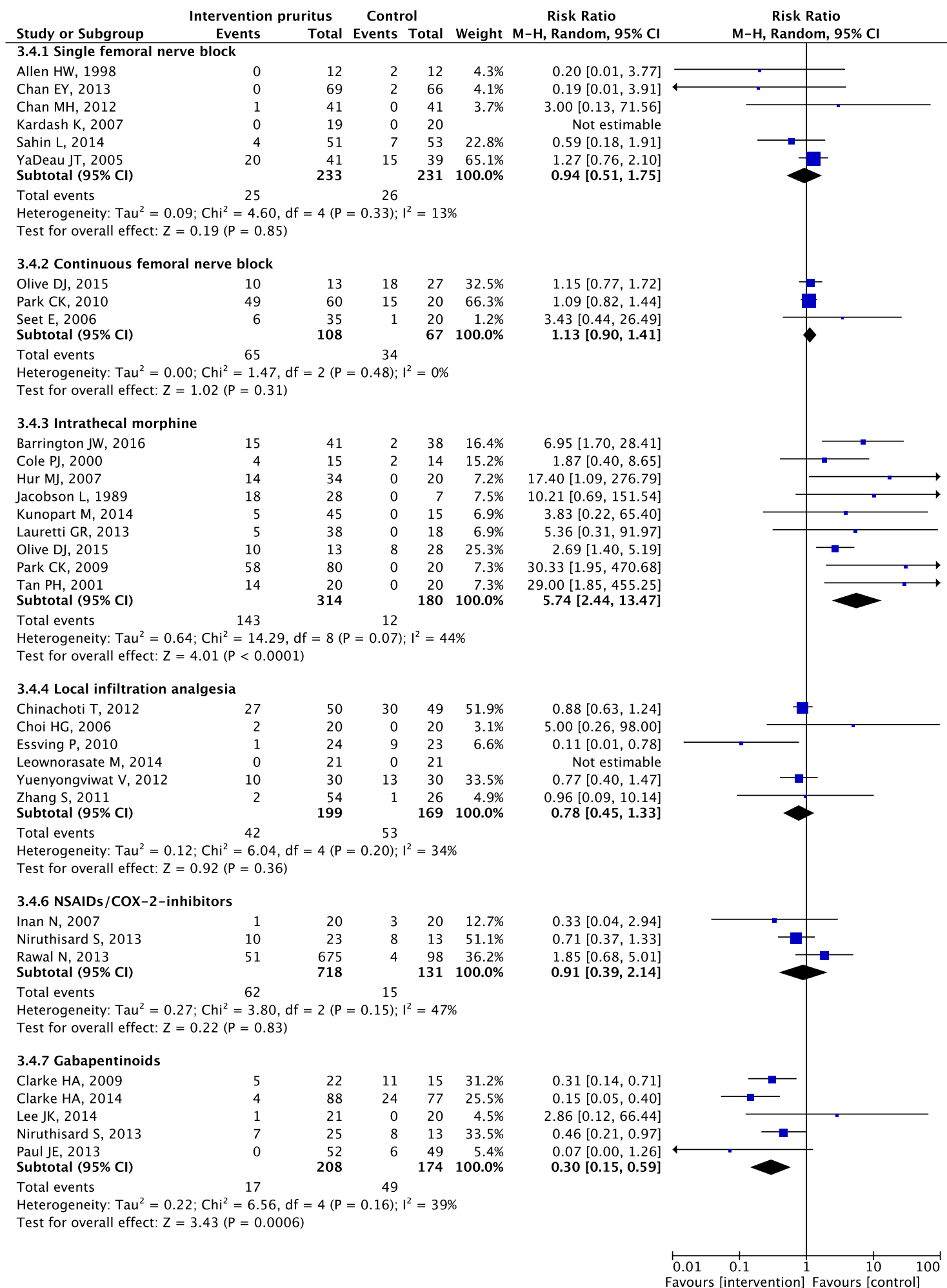

Supplement: S10 Appendix — Blue squares with horizontal lines represent mean differences and 95% confidence intervals for each trial. Black tiles represent the mean difference of each intervention. (PDF) [file pone.0173107.s010.pdf]

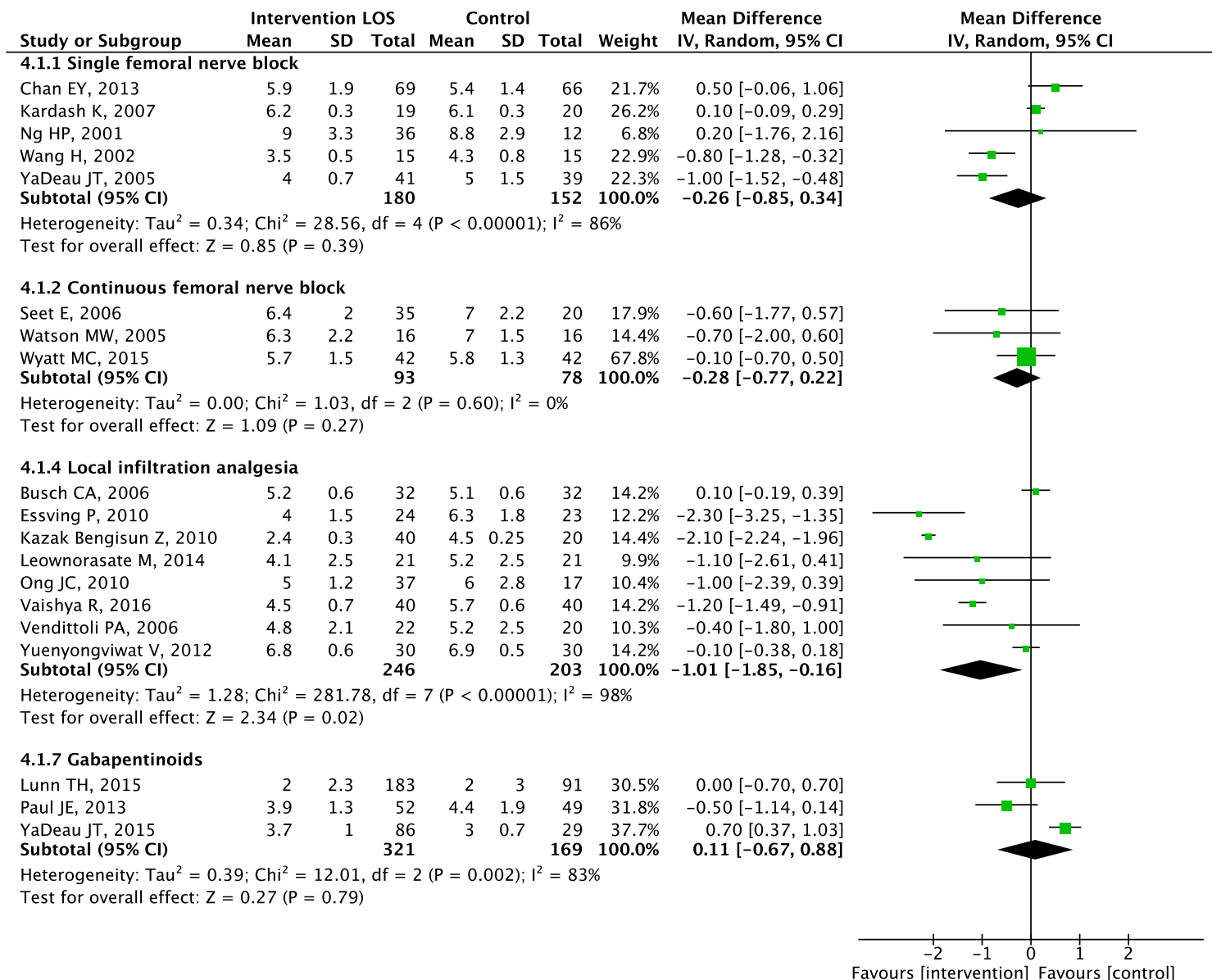

Supplement: S11 Appendix — Green squares with horizontal lines represent mean differences and 95% confidence intervals for each trial. Black tiles represent the mean difference of each intervention. (PDF) [file pone.0173107.s011.pdf]

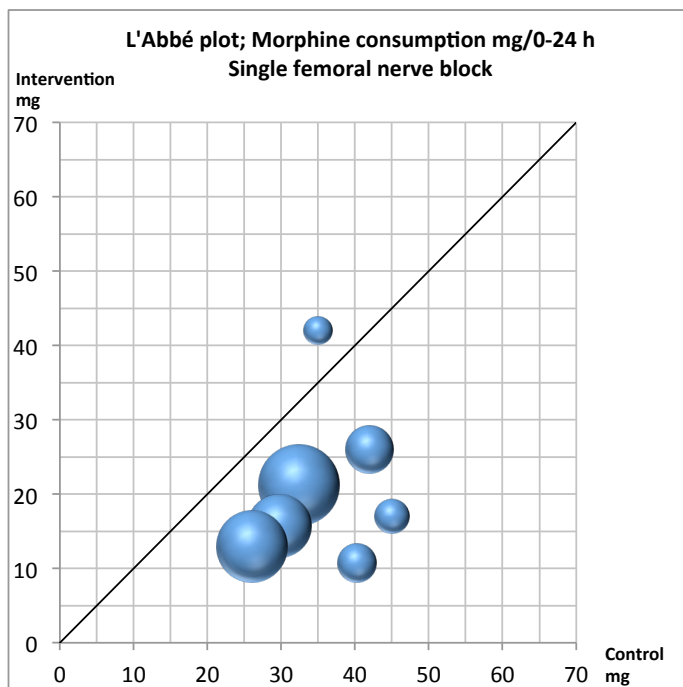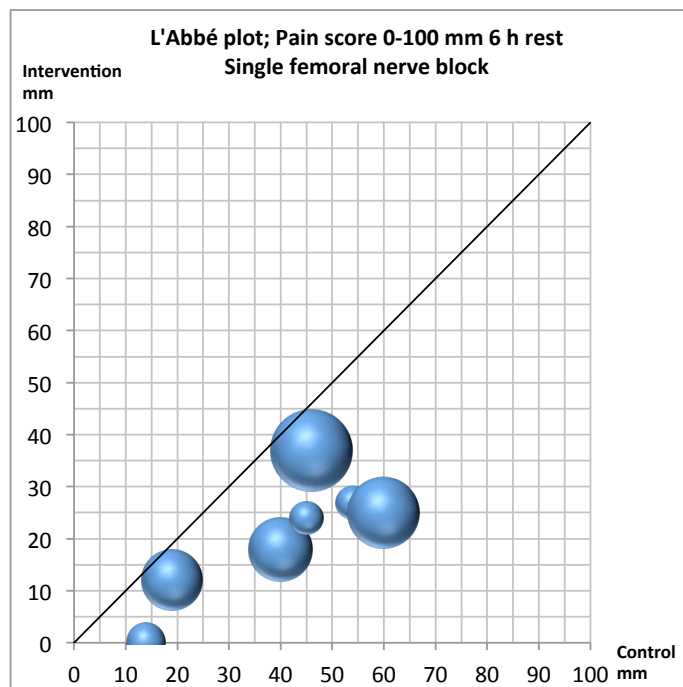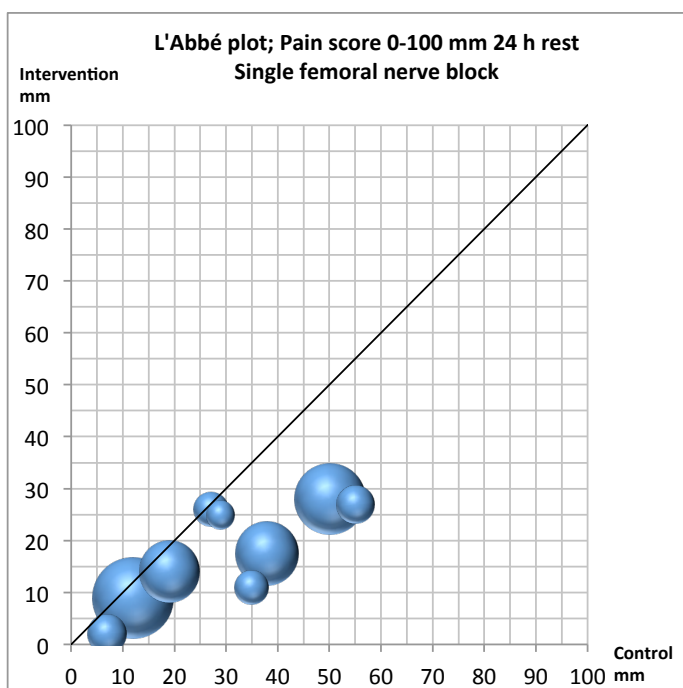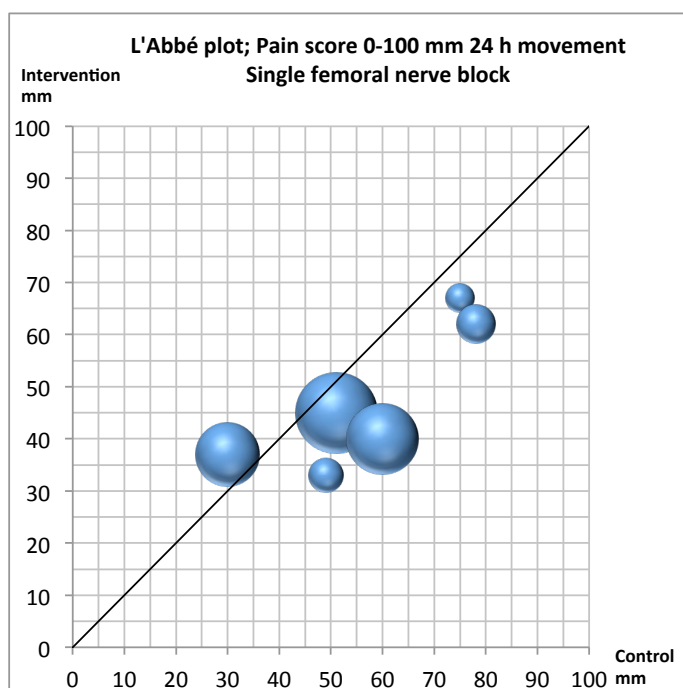

Supplement: S12 Appendix — Higher degrees of heterogeneity were demonstrated for pain at 6 and 24 hours rest and 24 hours movement. The size of the ball resembles the number of included patients in that trial and it is standardized across the different plots. (PDF) [file pone.0173107.s012.pdf]

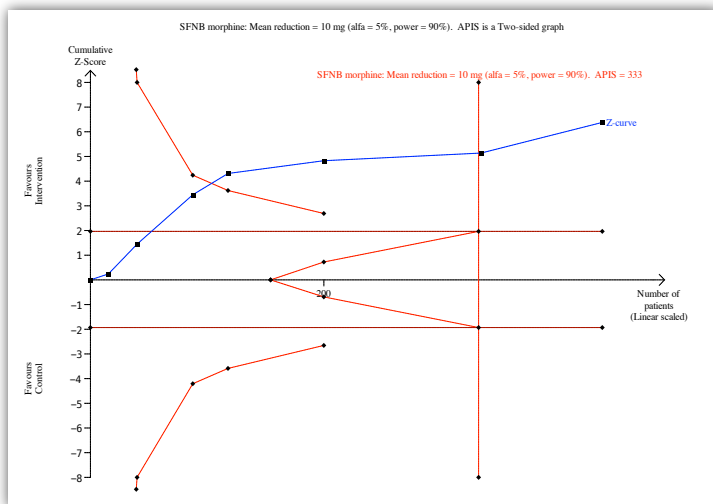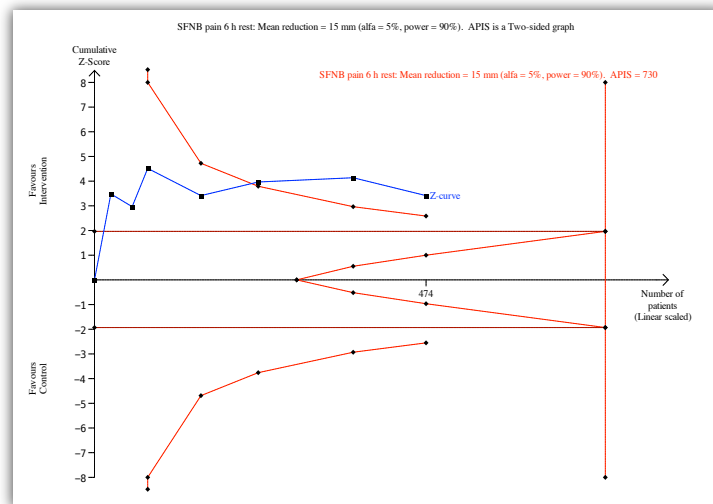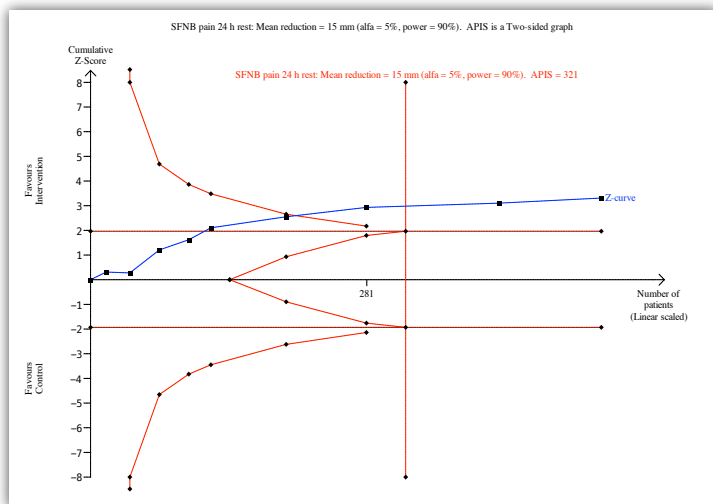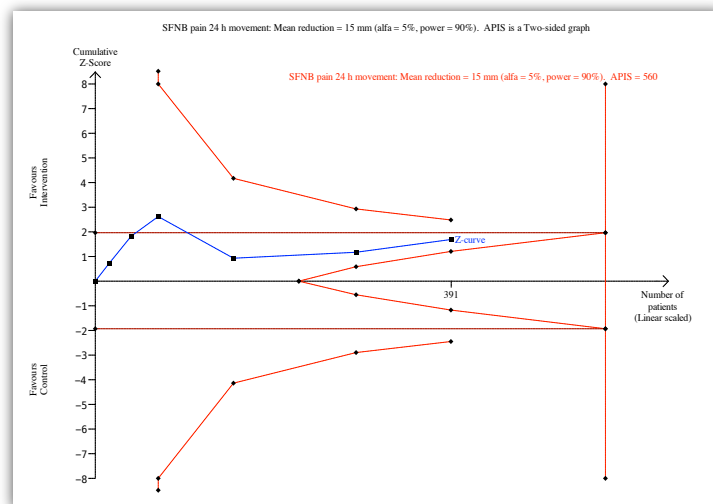

Supplement: S13 Appendix — A priori estimated information sizes (APIS) (333, 730, 321 and 560 patients, respectively) were based on an alpha-value of 0.05 and a beta-value of 0.9. The sensitivity to detect a mean difference for opioid consumption was predefined as 10 mg morphine equivalents 0–24 h postoperative and for pain scores a mean difference of 15 mm (VAS 0–100 mm). The blue line depicts the cumulative Z-score of the meta-analysis. The outer red lines illustrate the sequential z-score threshold for significance. The inner red lines illustrate the area of futility. The burgundy lines represent a stationary Z-score at 1.96 corresponding to p = 0.05. Threshold for significance was reached for morphine consumption and pain at 6 and 24 hours rest. Morphine consumption and pain score at 24 hours rest reached APIS, concluding that the intervention has an effect on these outcomes. (PDF) [file pone.0173107.s013.pdf]

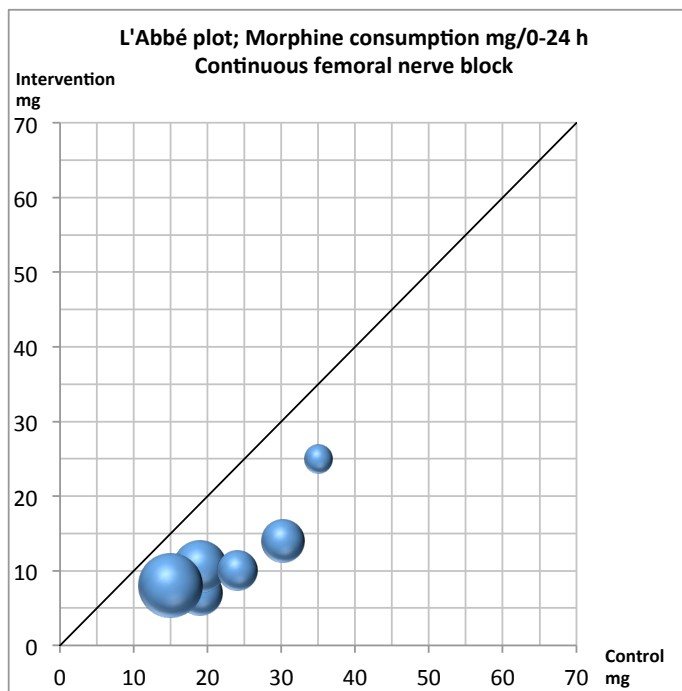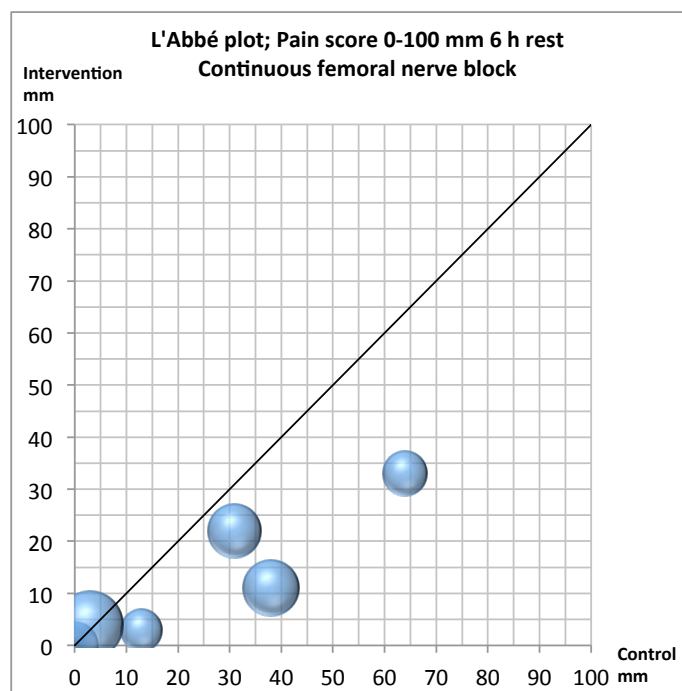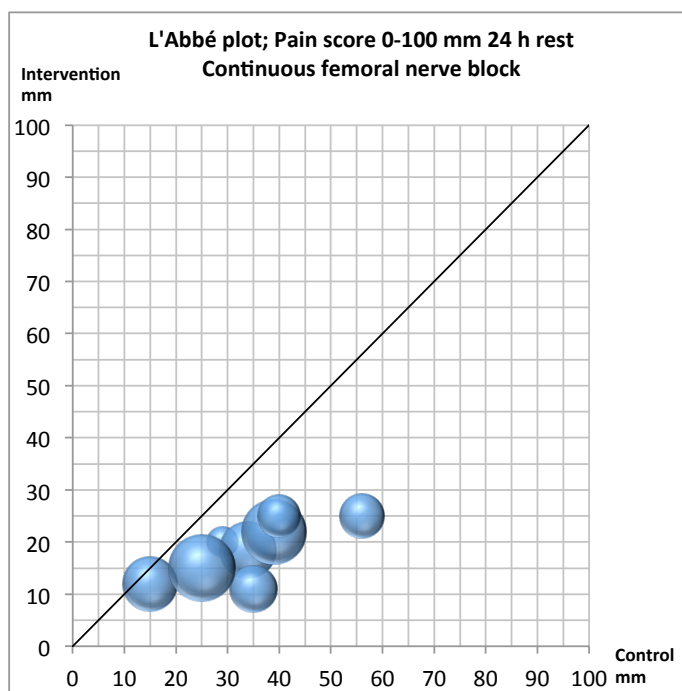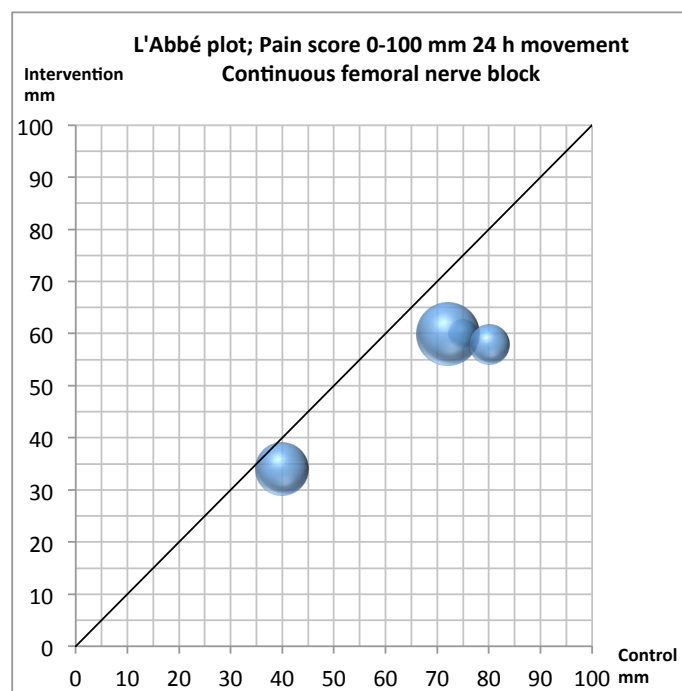

Supplement: S14 Appendix — Homogeneity was demonstrated for morphine consumption and pain scores. The size of the ball resembles the number of included patients in that trial and it is standardized across the different plots. (PDF) [file pone.0173107.s014.pdf]

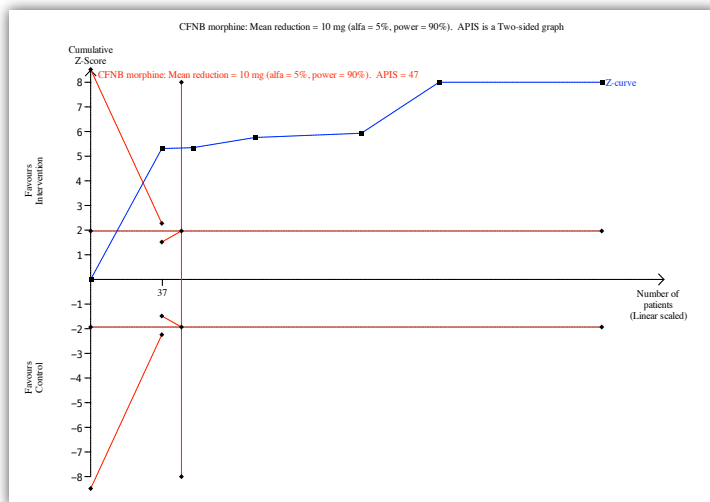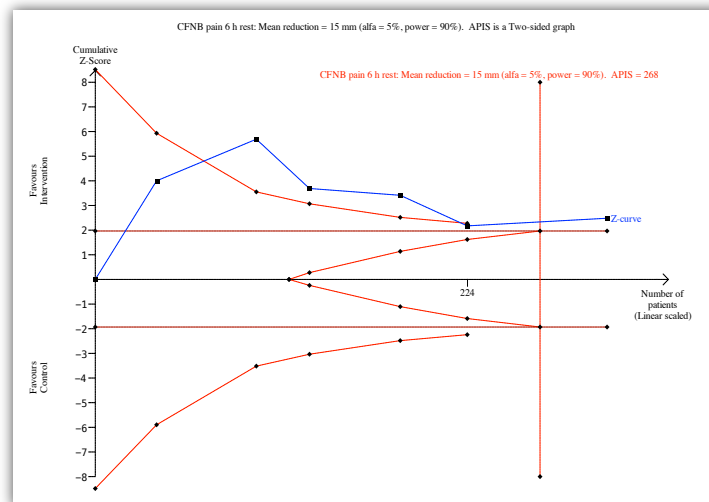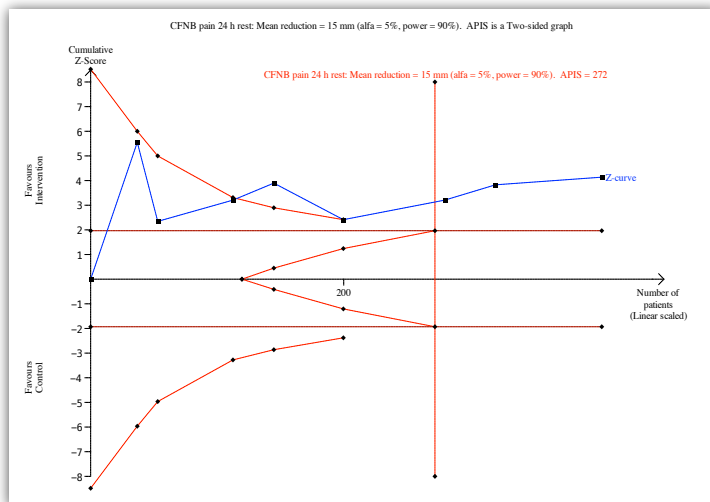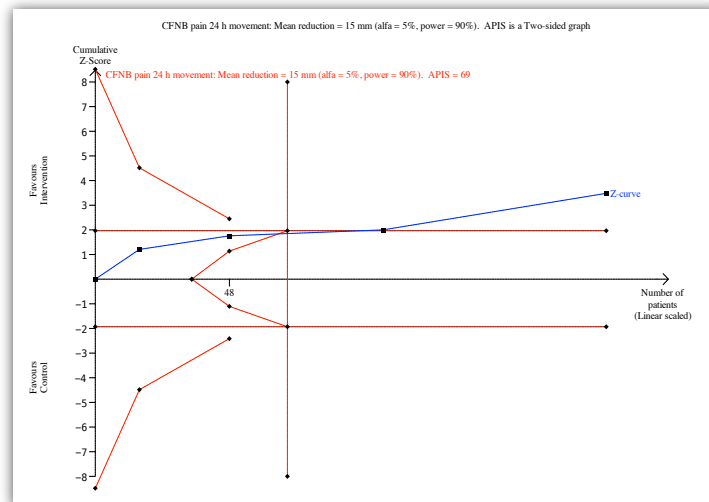

Supplement: S15 Appendix — A priori estimated information sizes (APIS) (47, 268, 272 and 69 patients, respectively) were based on an alpha-value of 0.05 and a beta-value of 0.9. Threshold for significance and APIS were reached for morphine consumption and all pain scores concluding that continuous femoral nerve block has a positive effect on these outcomes. For further elaboration see S13 Appendix. (PDF) [file pone.0173107.s015.pdf]

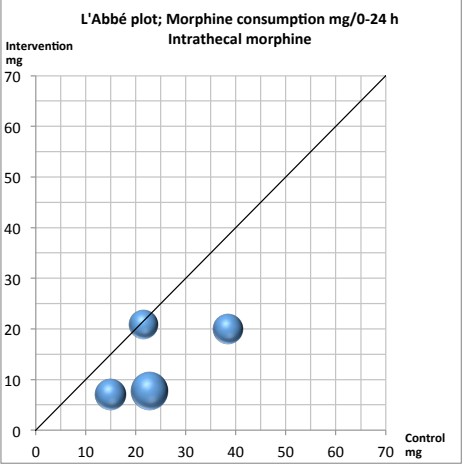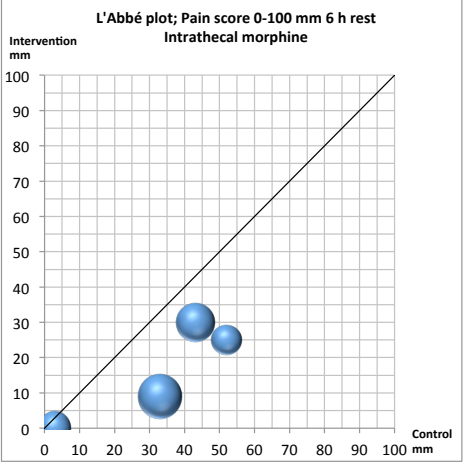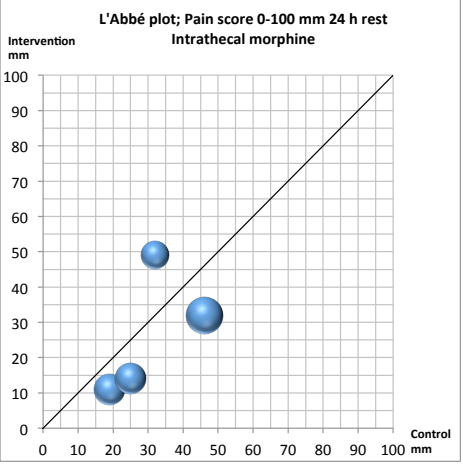

Supplement: S16 Appendix — Moderate degrees of heterogeneity were demonstrated for morphine consumption and pain scores. The size of the ball resembles the number of included patients in that trial and it is standardized across the different plots. (PDF) [file pone.0173107.s016.pdf]

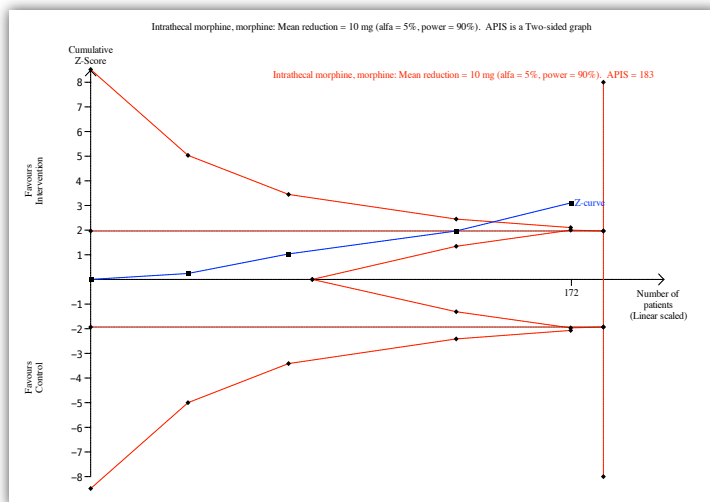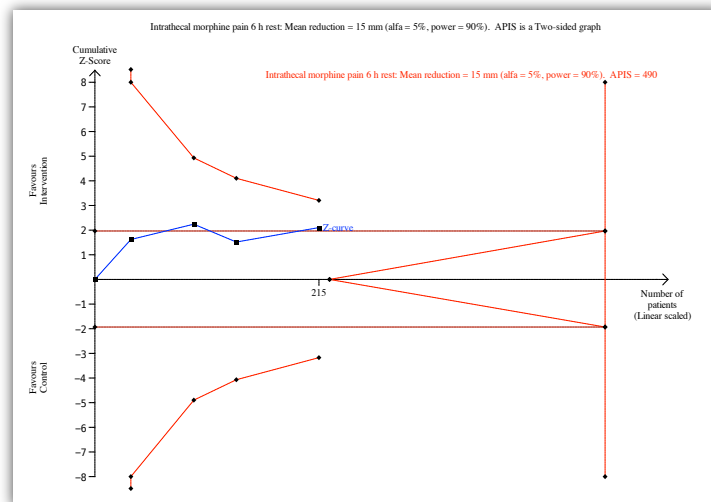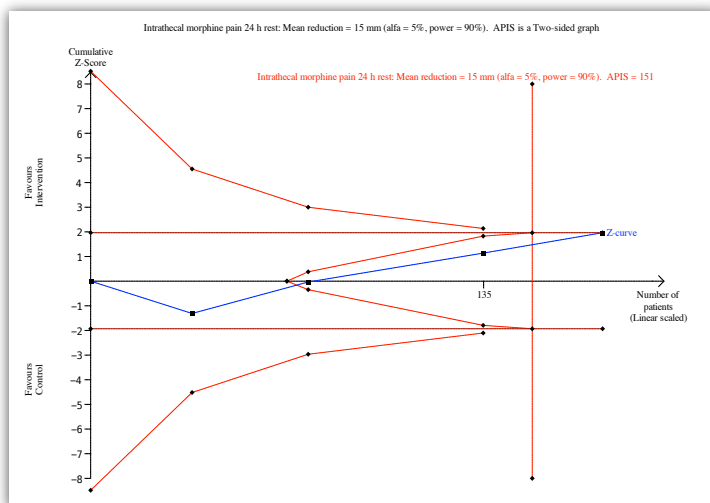

Supplement: S17 Appendix — A priori estimated information sizes (APIS) (183, 490 and 151 patients, respectively) were based on an alpha-value of 0.05 and a beta-value of 0.9. Morphine consumption reached the threshold for significance but not APIS. Pain score at 24 hours rest reached the boundary for futility and APIS concluding that there is no reason for further investigation of this outcome. For further elaboration see S13 Appendix. (PDF) [file pone.0173107.s017.pdf]

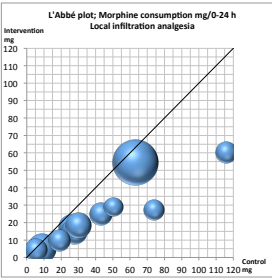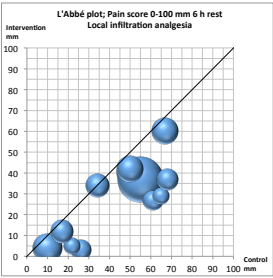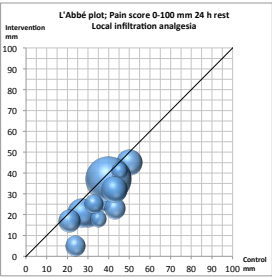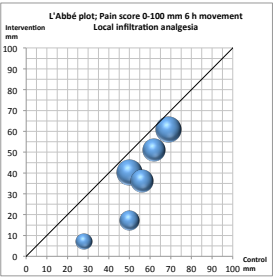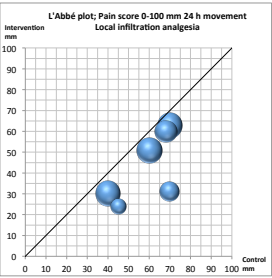

Supplement: S18 Appendix — Lower degrees of heterogeneity were demonstrated for morphine consumption and pain scores at rest. Moderate degrees were present for pain scores at movement. The size of the ball resembles the number of included patients in that trial and it is standardized across the different plots. (PDF) [file pone.0173107.s018.pdf]

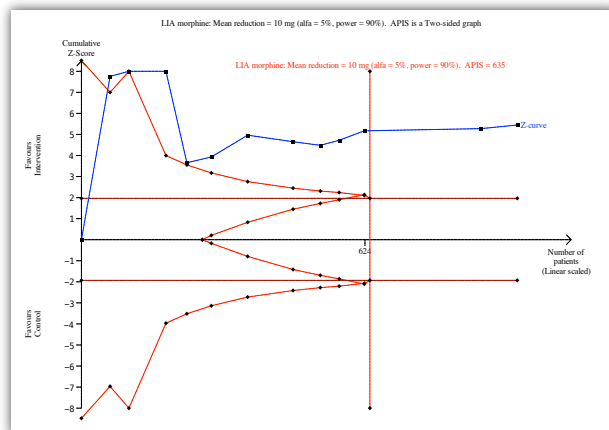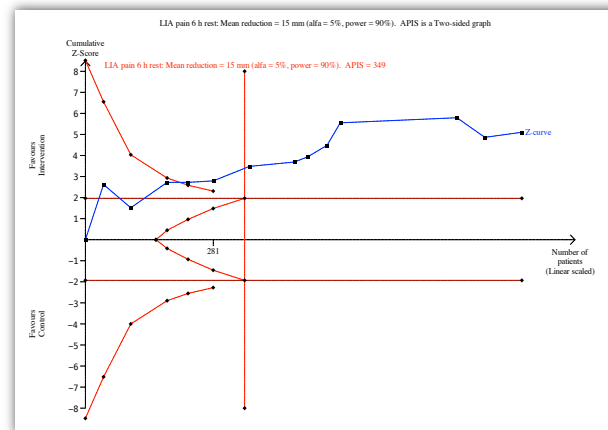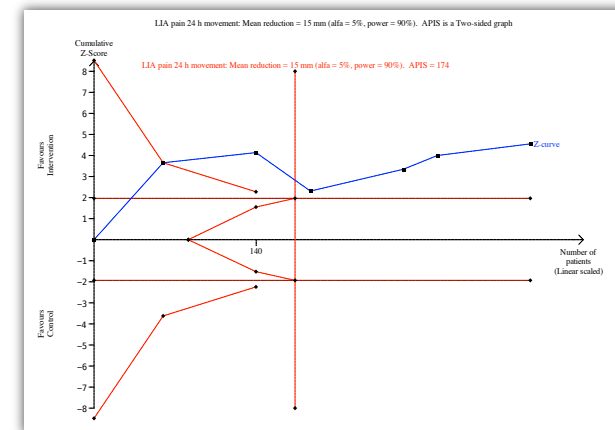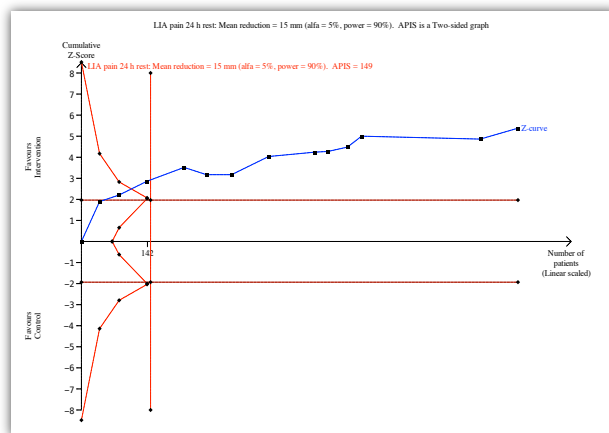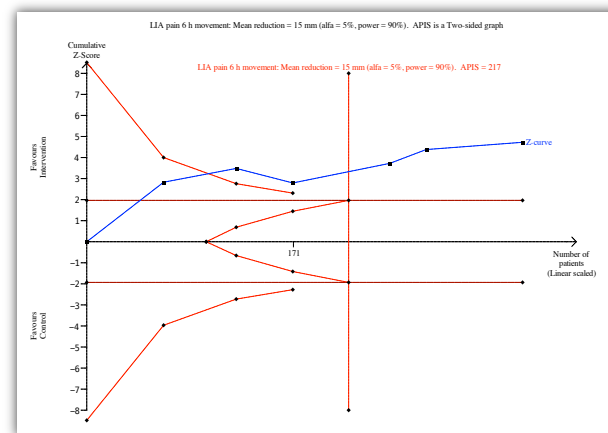

Supplement: S19 Appendix — A priori estimated information sizes (APIS) (635, 349, 149, 217 and 174 patients, respectively) were based on an alpha-value of 0.05 and a beta-value of 0.9. Threshold for significance and APIS were reached for all end-points, concluding that LIA has a positive effect on these outcomes. For further elaboration see S13 Appendix. (PDF) [file pone.0173107.s019.pdf]

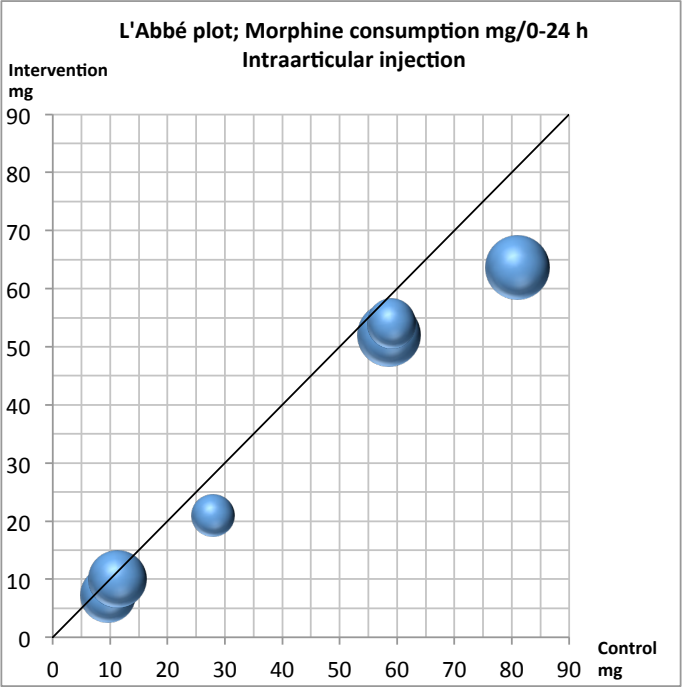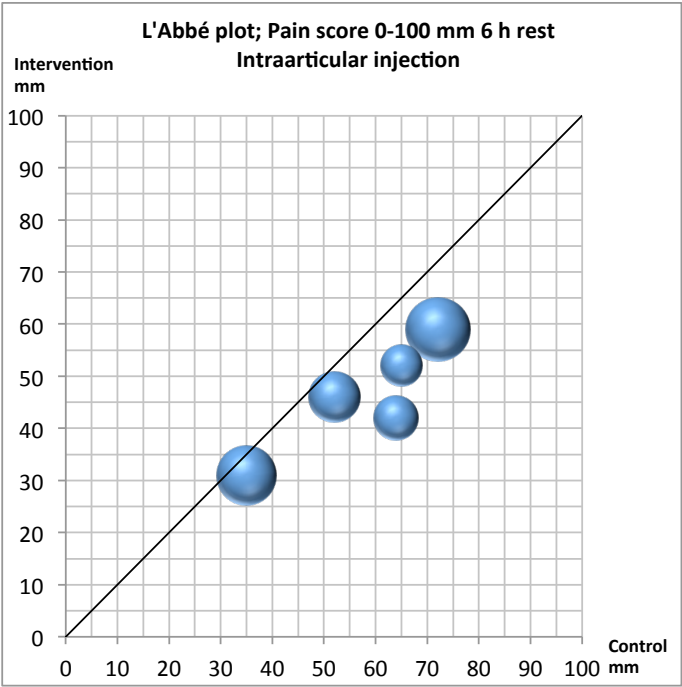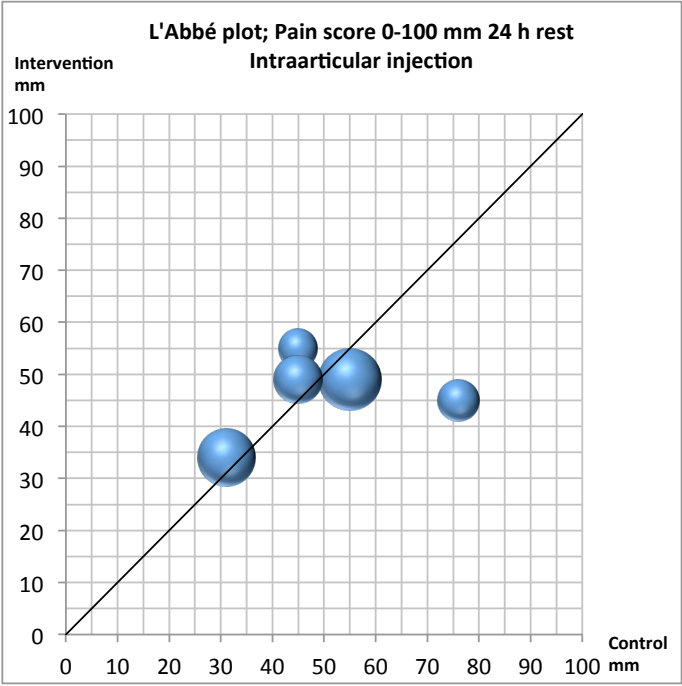

Supplement: S20 Appendix — Homogeneity was demonstrated for morphine consumption and pain at 6. Pain scores at 24 hours at rest were heterogeneous. The size of the ball resembles the number of included patients in that trial and it is standardized across the different plots. (PDF) [file pone.0173107.s020.pdf]

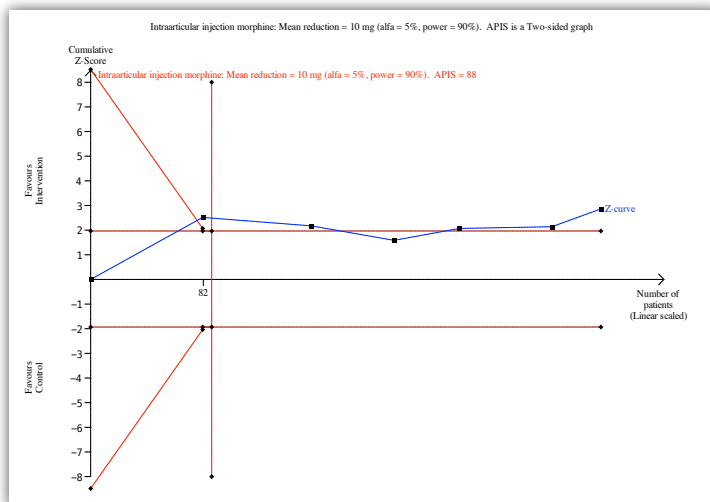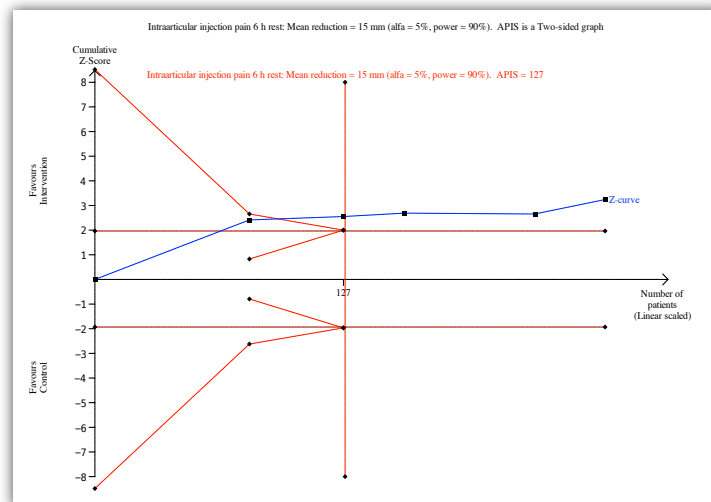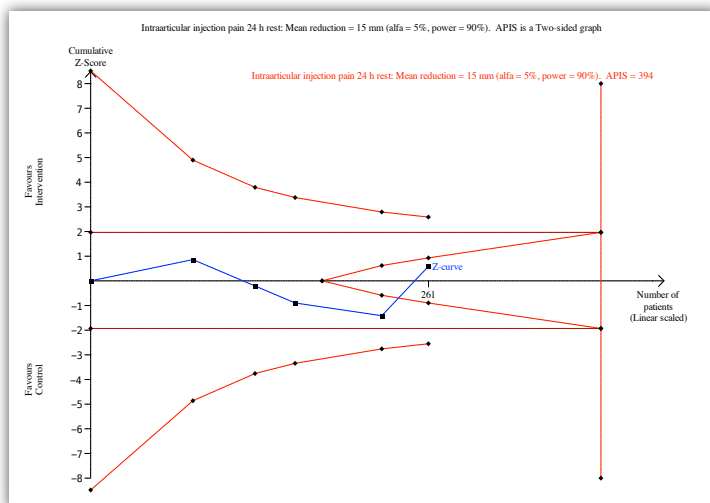

Supplement: S21 Appendix — A priori estimated information sizes (APIS) (88, 127 and 394 patients, respectively) were based on an alpha-value of 0.05 and a beta-value of 0.9. Threshold for significance and APIS were reached for morphine consumption and pain at 6 hours rest concluding that intraarticular injection has a positive effect on these outcomes. For further elaboration see S13 Appendix. (PDF) [file pone.0173107.s021.pdf]

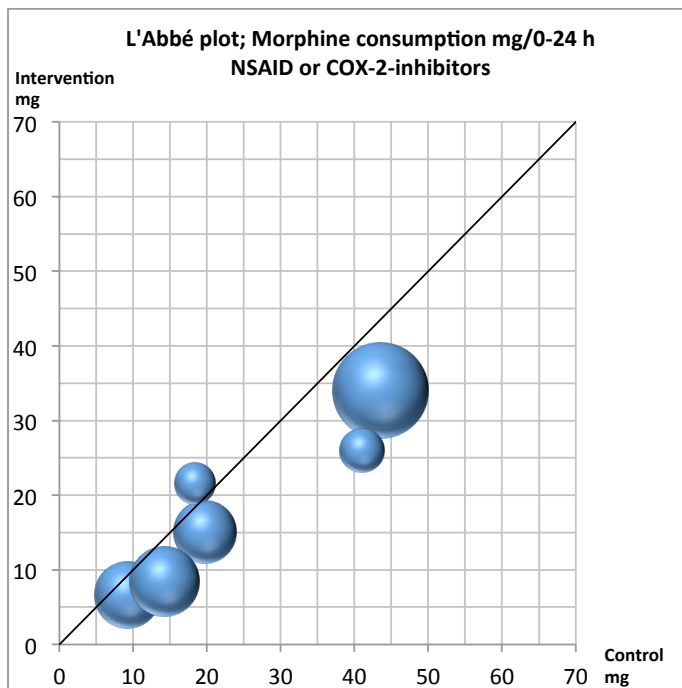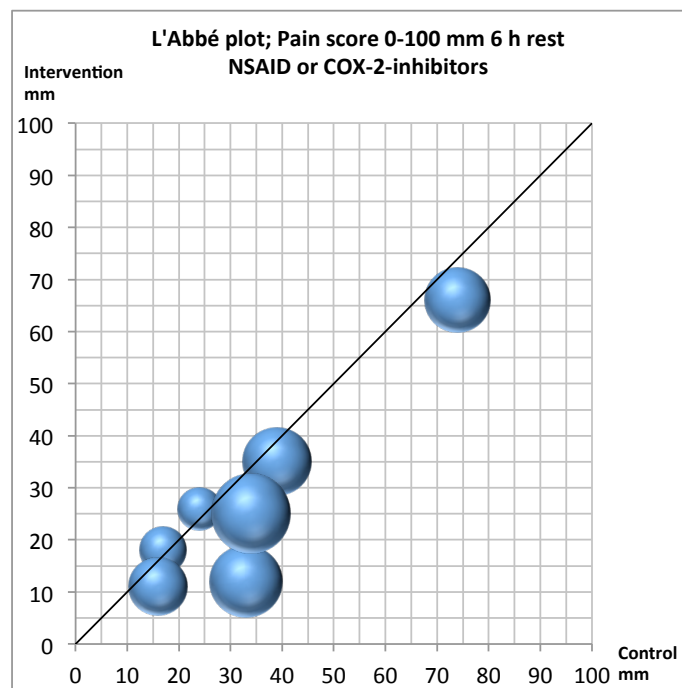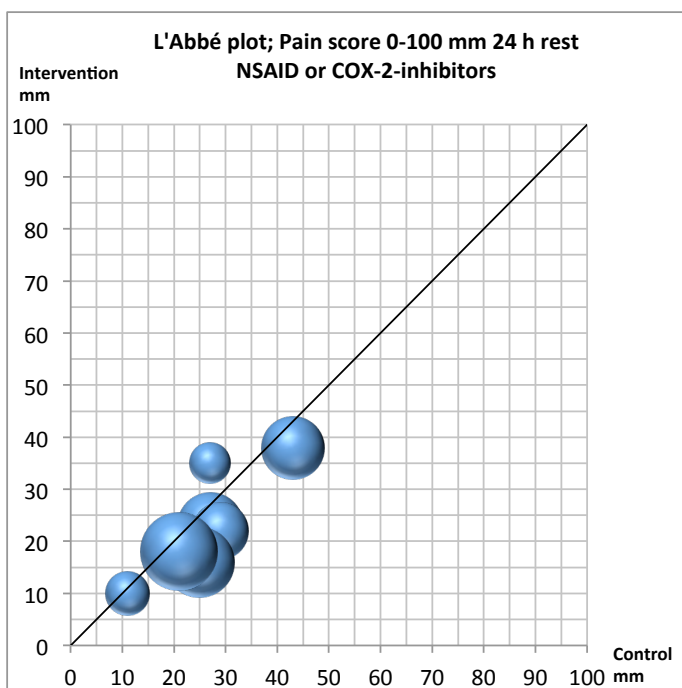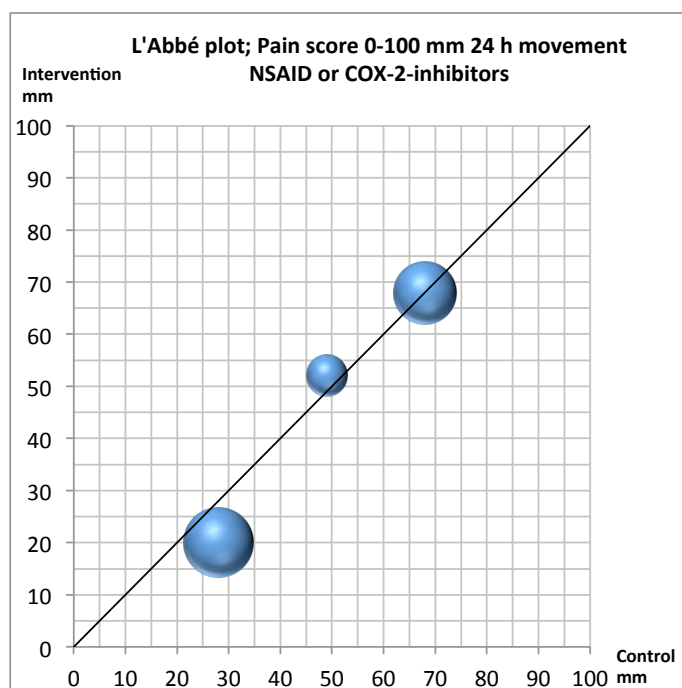

Supplement: S22 Appendix — Lower degrees of heterogeneity were demonstrated for morphine consumption and moderate degrees of heterogeneity for pain scores. The size of the ball resembles the number of included patients in that trial and it is standardized across the different plots. (PDF) [file pone.0173107.s022.pdf]

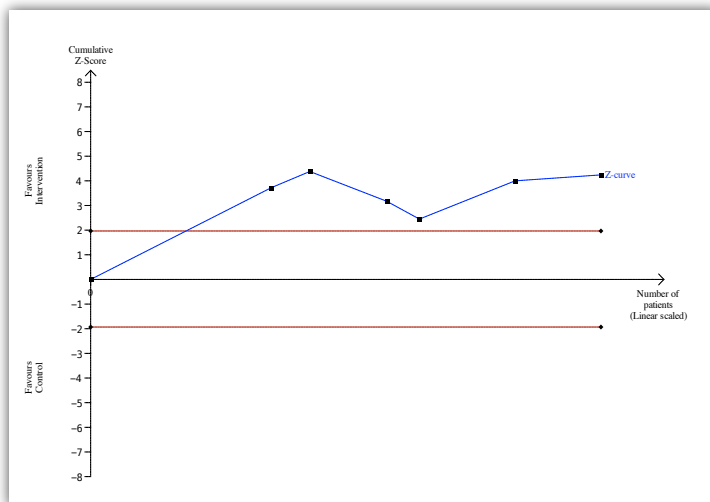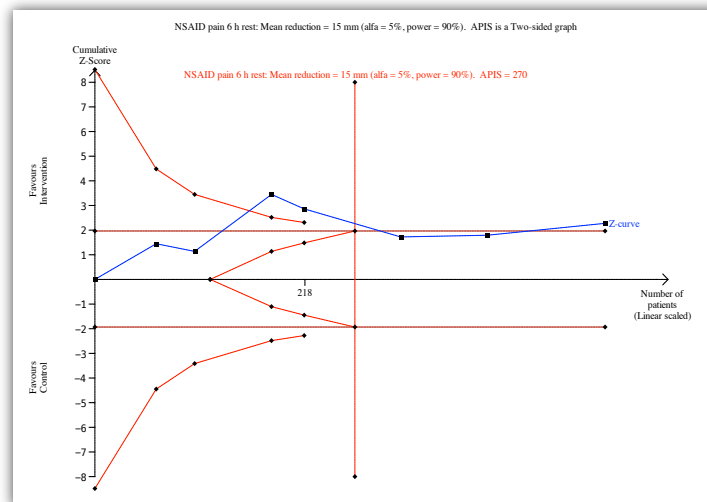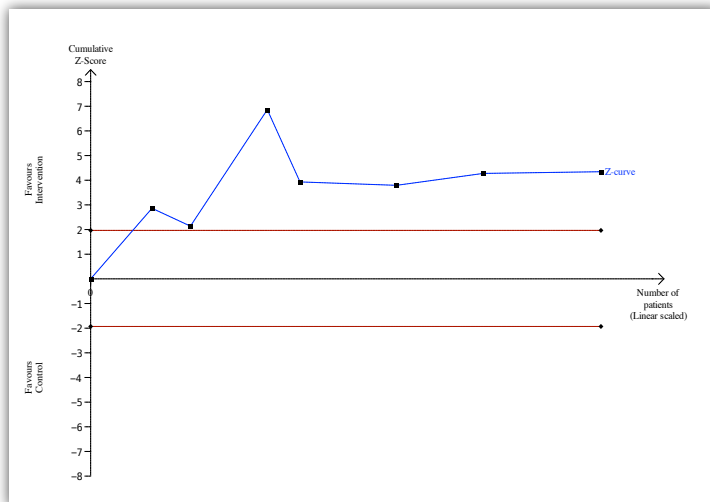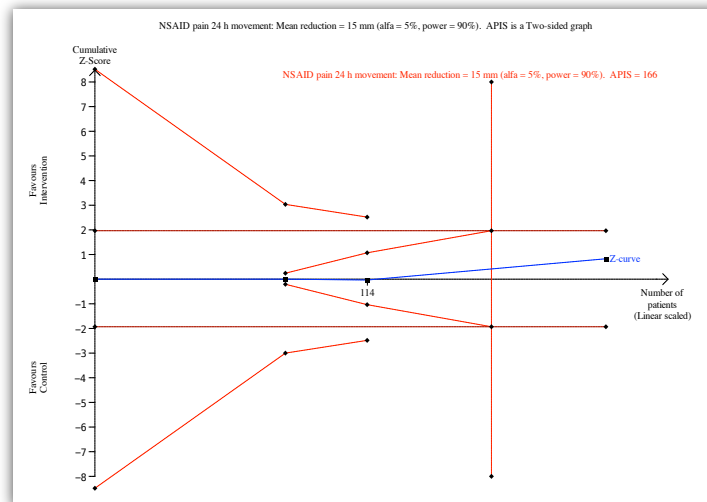

Supplement: S23 Appendix — A priori estimated information sizes (APIS) (115, 270, 32 and 166 patients, respectively) were based on an alpha-value of 0.05 and a beta-value of 0.9. Threshold for significance and APIS were reached for morphine consumption and pain at 6 and 24 hours rest concluding that NSAIDs and COX-2-inhibitors has a positive effect on these outcomes. Morphine consumption and pain at 24 hours rest reached APIS with the first trial. Threshold for futility and APIS were reached for pain at 24 h movement concluding that further testing of this end-point is futile. For further elaboration see S13 Appendix. (PDF) [file pone.0173107.s023.pdf]

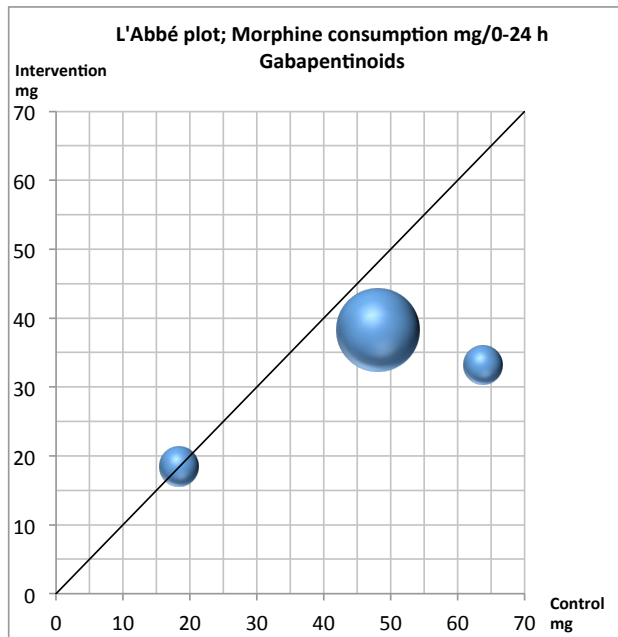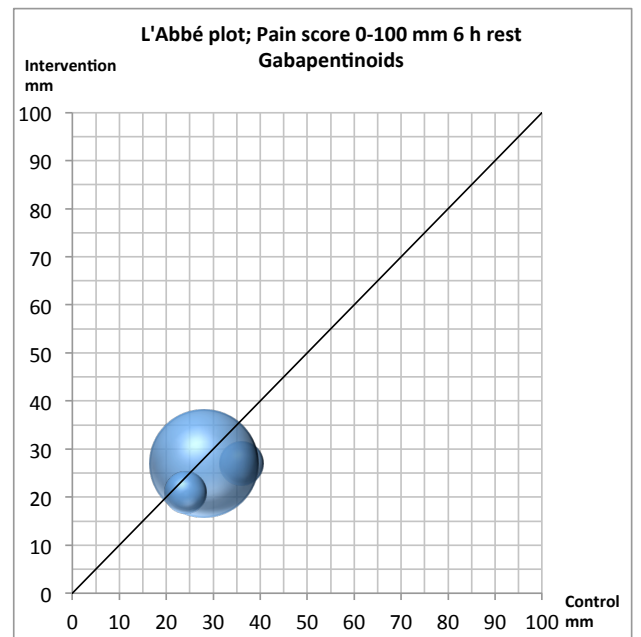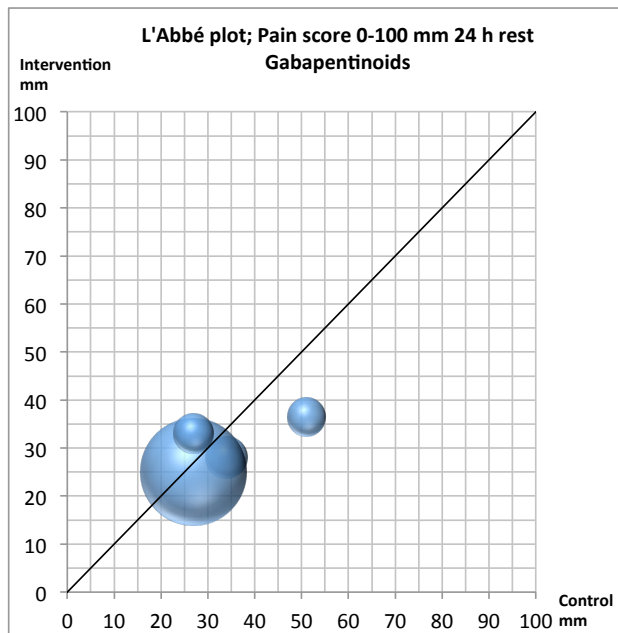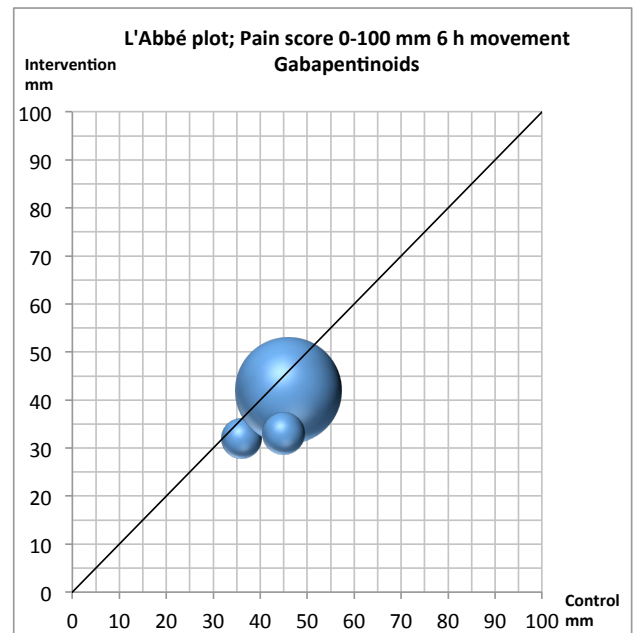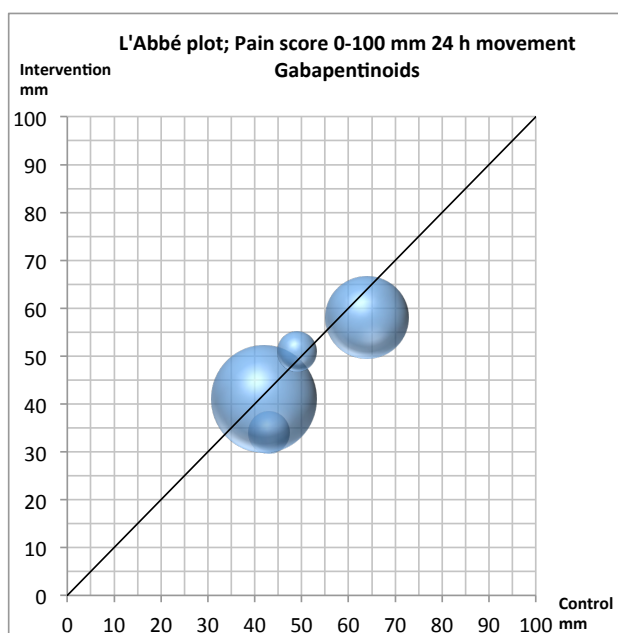

Supplement: S24 Appendix — Moderate degrees of heterogeneity was demonstrated for morphine consumption and pain at 24 hours rest. The size of the ball resembles the number of included patients in that trial and it is standardized across the different plots. (PDF) [file pone.0173107.s024.pdf]

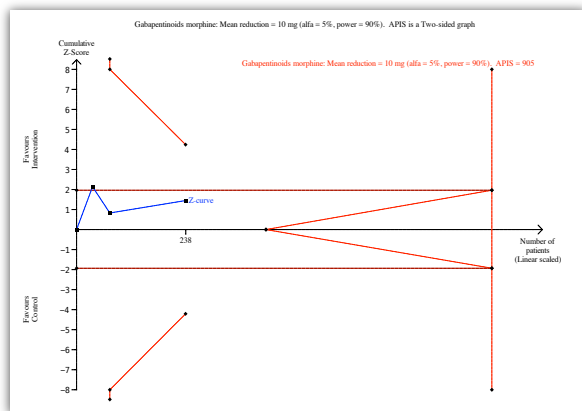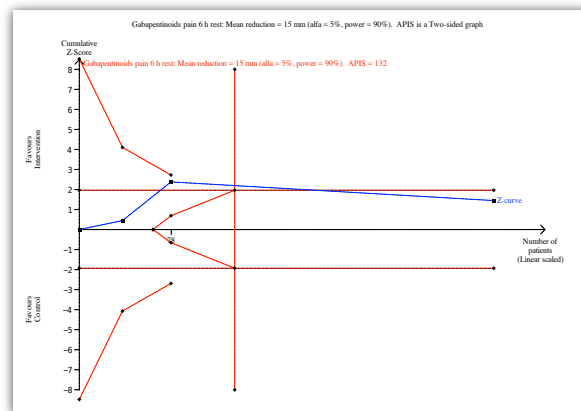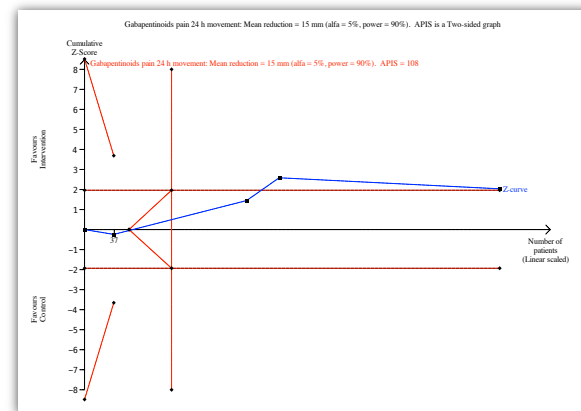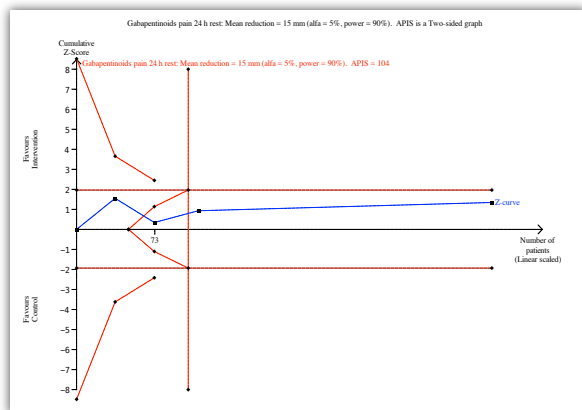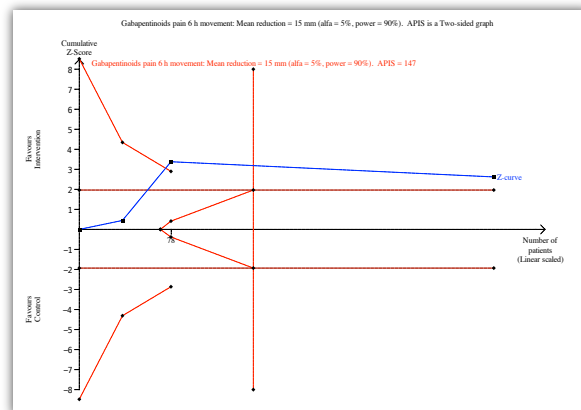

Supplement: S25 Appendix — A priori estimated information sizes (APIS) (905, 132, 104, 147 and 108 patients, respectively) were based on an alpha-value of 0.05 and a beta-value of 0.9. Threshold for significance and APIS were reached for pain at 6 and 24 hours at movement concluding that gabapentinoids have a positive effect on these outcomes. Threshold for futility and APIS were reached for pain at 6 and 24 h at rest concluding that further testing of these end-points is futile. For further elaboration see S13 Appendix. (PDF) [file pone.0173107.s025.pdf]
